# Diastereoselectivity of the Addition of Propargylic Magnesium Reagents to Fluorinated Aromatic Sulfinyl Imines

**DOI:** 10.1021/acs.orglett.1c01076

**Published:** 2021-04-21

**Authors:** Alberto Llobat, Jorge Escorihuela, Santos Fustero, Mercedes Medio-Simón

**Affiliations:** † Departamento de Química Orgánica, Universitat de València, Av. Vicent Andrés Estellés s/n, 46100 Burjassot, València, Spain

## Abstract

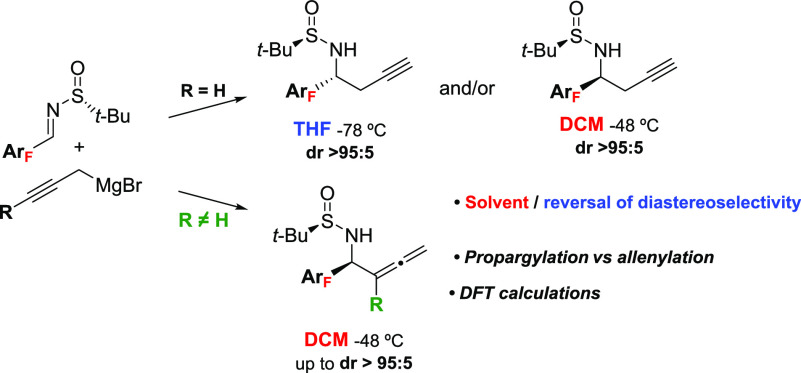

The addition of propargylmagnesium bromide to fluorinated aromatic sulfinyl imines gave homopropargyl amines with total regio- and diastereoselection. Complete reversal of diastereoselectivity can be achieved in some cases using coordinating (THF) or noncoordinating (DCM) solvents. Substituted propargylic magnesium reagents have been also tested toward fluorinated aryl sulfinyl imines affording chiral homoallenyl amines with good yields and selectivity control. DFT calculations helped to rationalize the origin of the experimental regio- and diastereoselectivities observed in each case.

Enantiomerically pure amines are interesting chiral building blocks that can be used in the synthesis of pharmaceutical drugs and in organometallic catalysis.^1^ The stereoselective 1,2-addition of organometallics to imines represents one of the most direct approaches for the synthesis of chiral amines,^2^ which is closely associated with the use of chiral *N*-sulfinyl imines due to their efficiency and availability. Among *N*-sulfinyl imines, *N*-*tert*-butylsulfinyl imines,^3^ extensively developed by Ellman, play an important role in this field due to their high chiral induction ability. The propargylation/allenylation of imines represents an interesting reaction leading to homopropargyl or homoallenyl amines, which requires both regio- and stereocontrol.^4^ Boron, tin, copper, silver, zinc, and indium reagents are usually employed to perform these synthetic reactions;^5^ however, magnesium reagents can also be efficient to afford homopropargylamines.^6^ Although the diastereoselective 1,2-addition of organometallic compounds to sulfinyl imines is a well-established procedure occurring with good yields and high diastereoselection,^7^ the outlook is highly dependent on the reaction conditions. Solvent effects on stereoselectivity, including enantio- and diastereoselectivity, are well documented in the literature, and several examples of dual stereocontrol have been reported.^8,9^ However, these studies are generally limited to showing the change in stereoselectivity in the presence of different solvents, bypassing a rationalization of the stereocontrol.

Continuing with our interest in organofluorine chemistry, which has important applications in pharmaceutical chemistry, agrochemistry and materials science,^10^ we noticed that the introduction of the trifluoromethyl group has received continuous attention,^11^ while aryl fluorinated groups such as tetrafluoro- and, in particular, pentafluoro-benzene derivatives have been disregarded, in spite of their interesting reactivity mainly associated with the possibility to perform nucleophilic substitution reactions.^12^ In a recent study, we found that the addition of propargylmagnesium bromide to alkylfluorinated sulfinyl imines was completely regioselective affording the corresponding homopropargylic amines without detection of allenic derivatives.^6^ For sulfinyl imines bearing a fluoroalkyl group (i.e., CF_3_) elevated diastereoselectivity (dr >95:5) was observed in THF, while a poor diastereoselection was obtained in dichloromethane (DCM) (dr 44:56) (Scheme 1). With these precedents in mind, we report our findings in the diastereoselective propargylation reaction of aryl fluorinated sulfinyl imines **1**, under different reaction conditions, paying attention to the solvent effect. Rationalization of the results is supported by theoretical calculations, which help to clarify in which way the diastereoselectivity is achieved.

**Scheme 1:**
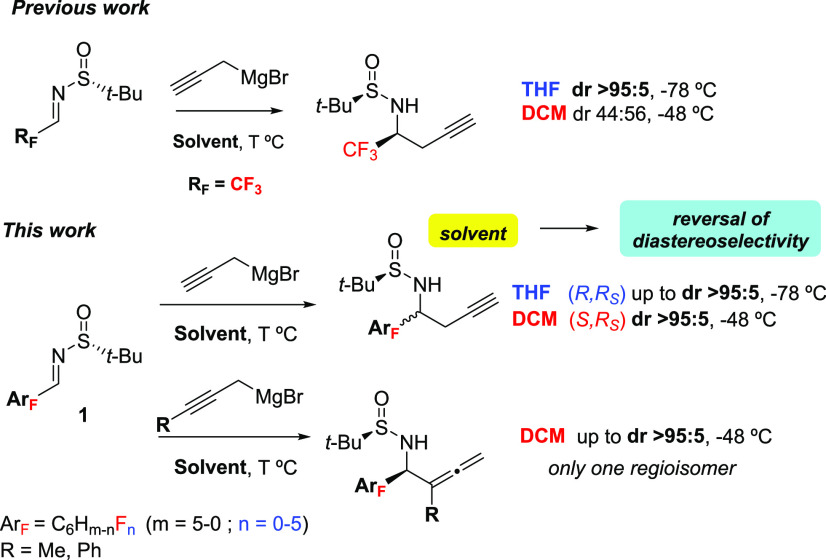
Previous Work Relevant to This Report

Our study began with the reaction of pentafluoroaryl sulfinyl imine **1a** with propargylmagnesium bromide (**2a**) as model substrates, using representative examples of coordinating (THF, DME, Me-THF, Et_2_O) and noncoordinating solvents (DCM, toluene) at −48 °C. The results of addition to (*R*)-*tert*-butylsulfinyl imine (**1a**) are summarized in Table 1. Among coordinating solvents (Table 1, entries 1–5), THF was the optimal solvent in terms of conversion. Despite DME affording product (*R*,*R*_S_)-**3a** with higher diastereoselectivity than THF (20:80 vs 33:67), the freezing point of DME hampers working below −58 °C, and therefore, the possibility of increasing diastereoselectivity. Interestingly, upon lowering the temperature to −78 °C in THF, the diastereoselectivity of **3a** was increased up to >95:5. When the reaction was performed in noncoordinating solvents, such as toluene, the opposite diastereomer, (*S*,*R*_S_)-**3′a**, was obtained with good yield and moderate diastereoselectivity (12:88) (Table 1, entry 6) unlike previously reported for fluoroalkyl substituted imines (i.e., CF_3_, dr 44:56).^6^ Noteworthy, when the addition of propargylmagnesium **2a** to imine **1a** was conducted in DCM, homopropargyl amine **3′a** was attained in good yield and high diastereoselectivity (dr >5:95) (Table 1, entry 7). The addition of a Lewis acid had no beneficial effect on the diastereoselectivity but resulted in lower yield, probably due to the reactivity of the Grignard reagent with BF_3_·Et_2_O (Table 1, entries 8–12). These results show that a complete reversal of diastereoselectivity can be achieved using coordinating or noncoordinating solvents, that is, THF at −78 °C or DCM at −48 °C. The major diastereomer was obtained for each solvent and was distinguishable by ^19^F NMR. Although the influence of solvents in diastereoselectivity in 1,2-addition of propargylic reagents to sulfinyl imines is well documented, a total reversion of the diastereoselection associated with a change in the solvent is unusual and never related with the presence or absence of fluorinated groups in the imine.^6,9^

**Table 1:**
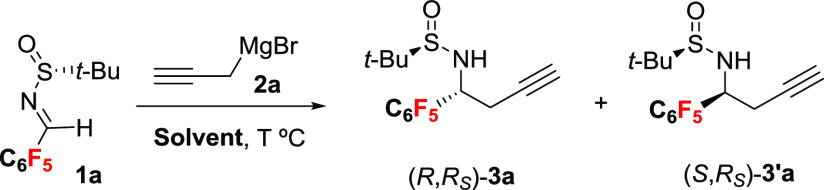
Optimization Study on the Diastereoselective 1,2-Addition of Propargyl Magnesium Bromide to Aromatic Sulfinyl Imine **1a**

entry	solvent	additive	*T* (°C)	yield^b^ (%)	dr^c^
1	THF		–48	**3a**, 99	67:33
2	Et_2_O		–48	**3a**, 65	67:33
3	DME		–48	**3a**, 70	80:20
4	Me-THF		–78	**3a**, 99	67:33
5	THF		–78	**3a**, 67	>95:5
6	toluene		–48	**3′a**, 78	12:88
7	DCM		–48	**3′a**, 80	>5:95
8	DCM	BF_3_·OEt_2_	–48	**3′a**, 41	33:67
9	toluene	BF_3_·OEt_2_	–48	**3′a**, 41	12:88
10	THF	BF_3_·OEt_2_	–48	**3a**, 99	67:33
11	Et_2_O	BF_3_·OEt_2_	–48	**3a**, 14	67:33
12	DME	BF_3_·OEt_2_	–48	**3a**, 84	80:20

a Reaction conditions: propargylmagnesium bromide (1.5 equiv), solvent (0.1 M), 18 h.

b Isolated yield after column chromatography.

c Determined by ^19^F NMR; dr refers to **3a**/**3′a** ratio.

With the optimized reaction conditions in hand, we decided to test the propargylation reaction in THF at −78 °C and DCM at −48 °C for different aromatic sulfinyl imines **1** in which the number of fluorine atoms in the benzene ring was modulated. When the reaction was tested with sulfinyl imine **1b** having a C_6_HF_4_ substituent, the results were similar to those obtained for sulfinyl imine **1a**. However, when the reactions were carried out with sulfinyl imines bearing less than four fluorine atoms, the diastereoselectivity in THF showed a progressive erosion. Conversely, the high diastereoselectivity for sulfinyl imines **1a**–**f** was preserved in DCM as solvent. The absolute configuration was assigned based on X-ray analysis of crystals of compounds **3b** and **3′b**, which revealed the formation of (*R*,*R*_S_)-**3b** in THF and (*S*,*R*_S_)-**3′b** in DCM. These results suggest the existence of a correlation between the presence or absence of fluorine atoms in the sulfinyl imine and the type of solvent for the propargylation reaction.

The above results can be understood on the basis of the generally accepted models proposed to rationalize the facial selectivity in the addition of organometallics to imines, where coordination of N and O atoms to the metal plays a crucial role.^12^ In this scenario, the presence of fluorine atoms affects the basicity of the coordinating atoms of the sulfinyl imines, which may in turn affect the facial control of the selectivity. A natural bond orbital (NBO)^13^ analysis of charges of the different atoms in the sulfinyl imines revealed that charges on N, O, and C atoms of sulfinyl imines can be correlated with the number of fluorine atoms in the benzene ring (Table S1, Supporting Information). Similar charges were found for the pairs formed by sulfinyl imines **1f** and **1e**, **1d** and **1c**, and **1b** and **1a**, respectively, following similar trends as experimentally shown in Table 2. Therefore, the O basicity in imines **1b** and **1a** having four and five fluorine atoms in the benzene ring would be relatively poor, and thus, a coordinating solvent can compete with the O of the sulfinyl imine for the coordination to Mg. Alternatively, with a noncoordinating solvent, the O basicity would favor the coordination to Mg.

**Table 2:**
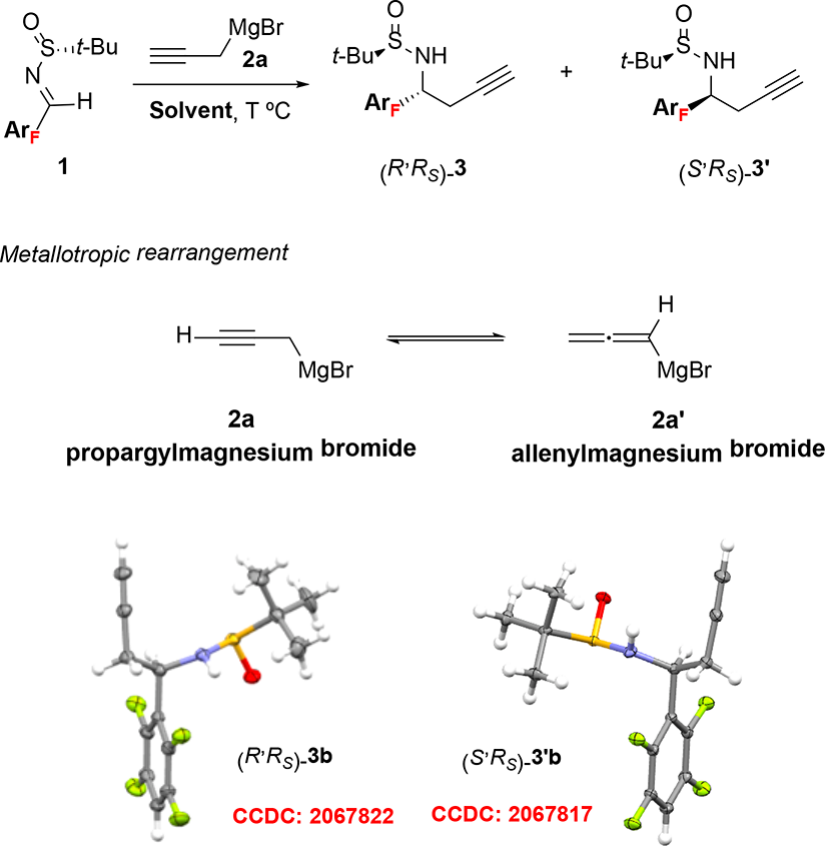
Diastereoselective 1,2-Addition of Propargyl Magnesium Bromide to Aromatic Sulfinyl Imines **1**

entry	Ar_F_	solvent	*T* (°C)	yield^b^ (%)	dr^c^
1	C_6_F_5_	THF	–78	**3a**, 67	>95:5
2	C_6_F_5_	DCM	–48	**3′a**, 80	>5:95
3	2,3,5,6-C_6_HF_4_	THF	–78	**3b**,^d^ 61	>95:5
4	2,3,5,6-C_6_HF_4_	DCM	–48	**3′b**,^d^ 68	>5:95
5	2,4,6-C_6_H_2_F_3_	THF	–78	**3c**, 84	67:33
6	2,4,6-C_6_H_2_F_3_	DCM	–48	**3′c**, 89	>5:95
7	2,6-C_6_H_3_F_2_	THF	–78	**3d**, 51	67:33
8	2,6-C_6_H_3_F_2_	DCM	–48	**3′d**, 70	>5:95
9	2-C_6_H_4_F	THF	–78	**3e**, 72	58:42
10	2-C_6_H_4_F	DCM	–48	**3′e**, 86	>5:95
11	C_6_H_5_	THF	–78	**3f**, 76	55:45
12	C_6_H_5_	DCM	–48	**3′f**, 80	>5:95

a Reaction conditions: **2a** (1.5 equiv), solvent (0.1 M), 18 h.

b Isolated yield after column chromatography.

c Determined by ^19^F NMR; dr refers to **3**/**3**′ ratio.

d X-ray analysis (see, Supporting Information for more details).

In order rationalize the observed diastereoselectivity, a computational study at wB97XD/6-311G(2d,2p) level of theory was carried out with Gaussian 16.^14^ The alkylation reaction of sulfinyl imines has been studied with MeMgBr but theoretical studies on propargylation have not been reported.^15^ The reaction of the starting propargyl bromide with magnesium gives the corresponding propargyl reagent **2a**, which after metallotropic rearrangement is converted into the allenyl magnesium reagent **2a′** (Table 2).^4^ DFT calculations showed that the allenyl intermediate is 4.6 kcal/mol more stable than the propargyl magnesium **2a**. Thus, **2a**′ will be the reactive species that by addition to the sulfinyl imine **1a** will afford homopropargyl amines **3a** or **3′a** through a S_E_2′ process. In the case of the reaction in noncoordinating solvents, the coordination of organomagnesium reagent **2a′** to sulfinyl imine **1a** is exergonic and N, S, O, and Mg are nearly coplanar (N–S–O–Mg dihedral angle 2.2°). A six-membered TS ring with the coordination of the magnesium atom to both the nitrogen and oxygen atoms of the imine facilitates the nucleophilic attack at the less hindered *Si* face for imines with the *R*_S_ configuration, as described for indium-promoted propargylation of chiral sulfinyl imines.^5f^ The calculated energy barrier of the TS for the attack from the *Si* or *Re* face is 4.8 and 8.3 kcal/mol, respectively. The difference in energy between the two transition states leading to the *S* and *R* products is 6.4 kcal/mol, which suggests that the *S*-product is mainly formed, in agreement with the observed diastereoselectivity (>5:95). Meanwhile, in the presence of a coordinating solvent (THF), the coordination of the magnesium atom to both the nitrogen of the imine and oxygen of a THF molecule is favored. In this scenario, the energy barrier of the TS for the attack from the *Re* face is 1.4 kcal/mol, whereas a higher barrier (12.1 kcal/mol) was found for the *Si* face attack. Therefore, the *R*-product is mainly formed in complete agreement with the experimental formation of the (*R*,*R*_S_)-diastereomer in THF. The plausible TS structures are shown in Figure 1. The distances between the imine carbon and the CH_2_ of the allenyl magnesium reagent are 2.28 Å (TS_*Si*-DCM_), 2.30 Å (TS_*Re*-DCM_), 2.32 Å (TS_*Si*-THF_), and 2.23 Å (TS_*Re*-THF_). The shortest distance corresponds to the TS with the lowest barrier (TS_*Re*-THF_) in accordance with Hammond’s postulate.

**Figure 1:**
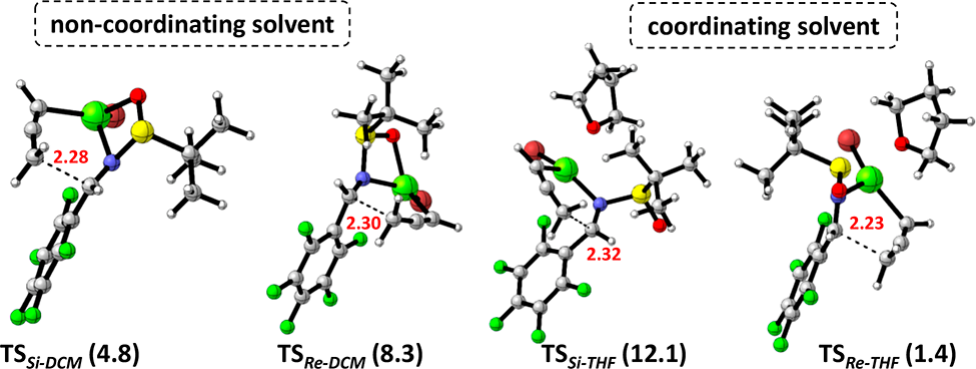
Optimized transition state structures at wB97XD/6-311G(2d,2p) for coordinating and noncoordinating solvents for **2a′** and **1a**. C···C bond forming distances are displayed in red. Relative activation energies are given in brackets in kcal/mol.

Next, we extended the study to disclose the behavior of representative examples of substituted propargylic Grignard reagents in the addition to aryl fluorinated sulfinyl imines **1** (Scheme 2) The addition of organomagnesium **2b** to imine **1a** resulted in total regioselectivity yielding allene **4ab** as the only regioisomer. The result is significant since, for this type of addition, the obtainment as single regioisomers of homoallenyl amines substituted at the α position in the allenic moiety is not frequent.^16^ High diastereoselectivity (>95:5) for the chiral homoallenyl amines was attained in DCM at −48 °C; however, diastereoselection was moderate in THF at −78 °C (dr 20:80). The major diastereomer attained was different for each solvent. For the rest of the sulfinyl imines of the series, high diastereoselectivities were also reached for the corresponding homoallenyl amines when noncoordinating DCM was used as solvent at −48 °C, with the sole exception of sulfinyl imine **1e**, having monofluorophenyl as substituent. Regarding the diastereoselectivity, organomagnesium **2c** behaved similarly to **2b** but in general yields were higher. In contrast, THF had a deleterious effect in the diastereoselection in all cases.

**Scheme 2:**
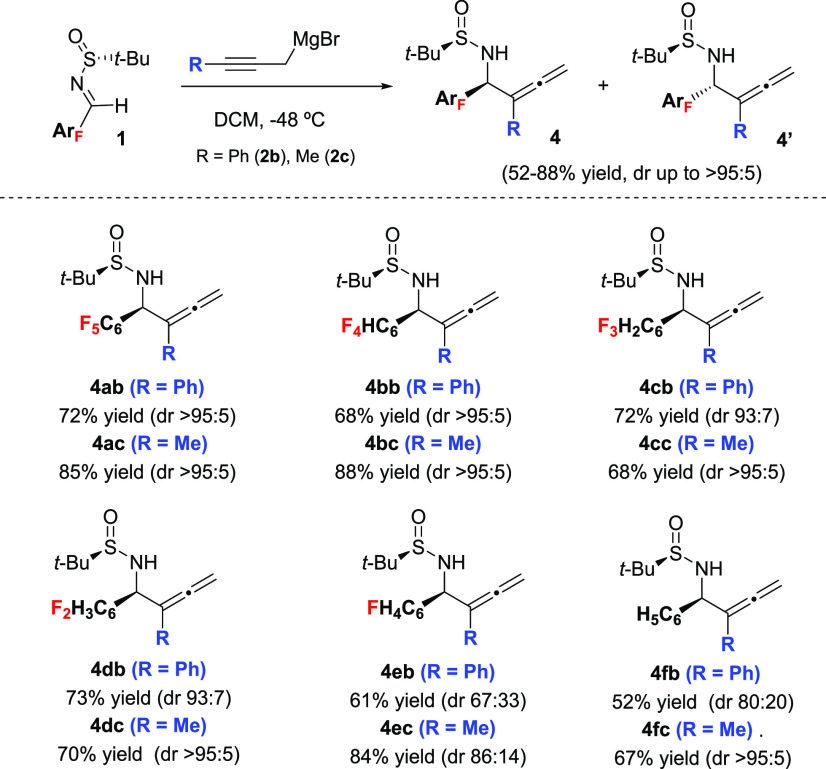
1,2-Addition of Substituted-Propargyl Magnesium Bromides to Fluorophenyl Sulfinyl Imines

The regioselectivity with substituted propargylic magnesium reagents can be ascribed to differences in the rate of equilibration of the corresponding propargylic and allenylic magnesium reagents.^17^ DFT calculations show a barrier of 6.4 and 5.3 kcal/mol for the transition states connecting the propargylic and allenylic magnesium species for **2b** and **2c**, respectively. These results suggest that isomerization is not fast and the regioselectivity is governed by the addition of propargylic magnesium reagent.^18^ Consistent with this mechanism,^4^ allenylic magnesium reagents afford propargylic products, and propargylic magnesium reagents provide allenylic products (Figure 2). DFT calculations in DCM show that the relative energy for the addition of the propargylic magnesium reagent is lower than that of the allenylic magnesium (1.5 vs 4.0 kcal/mol). This points out that the propargylic magnesium addition is a more favorable process compared to the allenylic magnesium addition, indicating that the homoallenyl amine is the only product, which is consistent with experimental results, where homopropargylic amines are not detected in the reaction media. The calculated energy for the TS also accounts for the findings in the diastereoselectivity of the allenylation reaction.

**Figure 2:**
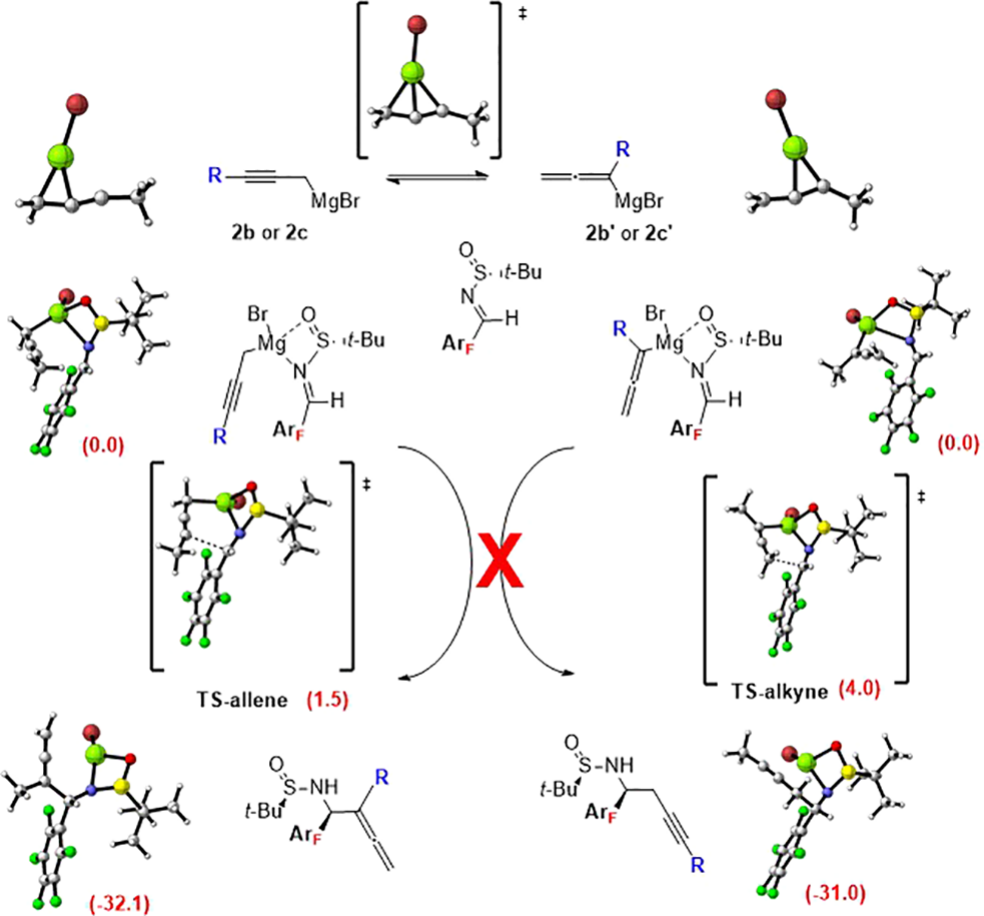
Proposed pathways for propargylation and allenylation of imine **1a** and optimized structures (for **1a** and **2c**) at wB97XD/6-311G(2d,2p) in DCM. Relative activation energies are given in brackets in kcal/mol.

In conclusion, we have disclosed that propargylation or allenylation of aryl fluorinated sulfinyl imines **1** can be performed in a regio- and diastereoselective way through S_E_^2^′ reaction of propargyl or substituted propargylic magnesium reagents, respectively. A marked dependence of the diastereoselectivity on the solvent and the basicity of the sulfinyl imine was observed. Coordinating solvents and high diastereoselectivities were compatible only with the less basic sulfinyl imines of the series meanwhile noncoordinating solvent allows good diastereoselection in all cases. Substituted propargylic magnesium reagents showed different behavior affording homoallenyl amines **4** as single regioisomers in noncoordinating solvents. DFT calculations helped to rationalize the experimental findings and to elucidate the mechanism supporting that coordination of N and O atoms (from the sulfinyl group or from the solvent) to the metal plays a crucial role in determining the diastereoselectivity of the propargylation/allenylation reaction. Further studies to extend its scope and complete its limitations are in progress.

## Supporting Information Available

Experimental details, NMR data, DFT calculations data, and X-ray crystal structures (PDF)

## Accession Codes

CCDC 2067817 and 2067822 contain the supplementary crystallographic data for this paper. These data can be obtained free of charge via www.ccdc.cam.ac.uk/data_request/cif, or by emailing data_request@ccdc.cam.ac.uk, or by contacting The Cambridge Crystallographic Data Centre, 12 Union Road, Cambridge CB2 1EZ, UK; fax: +44 1223 336033.
