# Supplementary material for: Diastereoselectivity of the Addition of Propargylic Magnesium Reagents to Fluorinated Aromatic Sulfinyl Imines
Source: Org Lett. 2021 Apr 21;23(9):3691–5. doi: 10.1021/acs.orglett.1c01076 (PMC9490847; doi:10.1021/acs.orglett.1c01076)

Supporting Information for

On the diastereoselectivity of the addition of propargylic magnesium reagents to fluorinated aromatic sulfinyl imines

Alberto Llobat, Jorge Escorihuela, Santos Fustero and Mercedes Medio-Simón*

Departamento de Química Orgánica, Universitat de València, Av. Vicent Andrés Estellés s/n, 46100 Burjassot, València, Spain.

Email: Mercedes.Medio@uv.es

Table of Contents.

	Page
I. General methods.	S3
II. General procedure for the condensation of *N*-*tert*-butanesulfinyl aldimines **1**.	S3
III. General procedure for the diastereoselective propargylation of sulfinyl imines.	S5
IV. General procedure for the propargylation reaction in DCM.	S9
V. X-ray structure of compound **3b**.	S14
VI. X-ray structure of compound **3'b**.	S20
VII. Computational methods.	S26
VIII. Natural bond orbital (NBO) analysis of charges of the different atoms in sulfinyl imines.	S26
IX. Cartesian coordinates of optimized structures.	S33
X. References.	S47
XI. ^{1}H, ^{13}C and ^{19}F NMR spectra of new compounds.	S48

I. General Methods.

Reactions were carried out under nitrogen atmosphere unless otherwise indicated. As a heat source oil baths were used. CH_2Cl_2 (DCM) was used without further purification. The reactions were monitored with the aid of TLC on 0.25 mm pre-coated silica-gel plates. Visualization was carried out with UV light and aqueous ceric ammonium molybdate solution or potassium permanganate stain. Flash column chromatography was performed with the indicated solvents on silica gel 60 (particle size: 0.040–0.063 mm). ^{1}H, ^{13}C and ^{19}F NMR spectra were recorded on a 300 MHz Bruker Avance III 300 spectrometer. Chemical shifts are given in ppm (δ), referenced to the residual proton resonances of the solvents. Coupling constants (*J*) are given in Hertz (Hz). The letters m, s, d, t, and q stand for multiplet, singlet, doublet, triplet, and quartet, respectively. The letters br indicate that the signal is broad. DEPT experiments were performed to assign CH, CH_2 and CH_3. A QTOF mass analyzer system has been used for HRMS measurements. Melting points were measured on a Büchi B−540 apparatus and are uncorrected. Optical rotations were measured on a Jasco P−1020 polarimeter at 25 °C.

II. General procedure for the condensation of *N-tert*-butanesulfinyl aldimines 1.

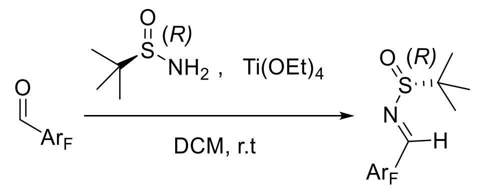

1a-f

The corresponding aldehyde (5 mmol) was dissolved in DCM (0.1 M) at room temperature in a round-bottomed flask. Titanium tetroxide (IV) (20 mmol) and (*R*)-*tert*-butylsulfinamide (6 mmol) were added and the mixture was stirred at room temperature overnight. Once the reaction was complete (TLC analysis), an aqueous saturated solution of $NaHCO_3$ was added and the mixture was filtered on Celyte® in order to remove the titanium salts. Finally, the filtered organic phase is dried over anhydrous Na_2SO_4, concentrated under reduced pressure and the crude mixture was purified by column chromatography using deactivated silica gel (*n*-hexane:EtOAc).

(*R*,*E*)-2-Methyl-*N*-((perfluorophenyl)methylene)propane-2-sulfinamide (1a). According to general procedure from 1.00 g (5.1 mmol) of 2,3,4,5,6-pentafluorobenzaldehyde, compound **1a** was obtained as a yellow solid after column chromatography on silica gel using *n*-hexane:EtOAc (4:1) as eluent (1.28 g, 84% yield). Mp: 96–98 °C; $[\alpha]^{25}_D$ = –55.1 (c 1.0, $CHCl_3$); ^{1}H NMR (300 MHz, $CDCl_3$): δ (ppm) 8.71 (s, 1H), 1.27 (s, 9H); ^{19}F NMR (282 MHz, $CDCl_3$): δ (ppm) -139.90 – -140.05 (m, 2F), -147.20 – -147.38 (m, 1F), -160.75 – -160.96 (m, 2F); ^{13}C {^{1}H} NMR (75 MHz, $CDCl_3$): δ (ppm) 151.2, 148.1–144.3 (m, 2C–F), 145.3–141.5 (m, 1C–F), 139.8–135.9 (m, 2C–F), 109.7–109.4 (m, 1C), 58.5, 22.5. HRMS (ESI) m/z: [M + H$^+$] Calcd for $C_{11}H_{11}F_5NOS$ 300.0403; Found 300.0409.

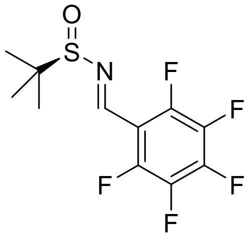

(*R*,*E*)-2-Methyl-*N*-(2,3,5,6-tetrafluorobenzylidene)propane-2-sulfinamide (1b). According to general procedure from 500 mg (2.81 mmol) of 2,3,5,6-tetrafluorobenzaldehyde, compound **1b** was obtained as a white solid after column chromatography on silica gel using *n*-hexane:EtOAc (4:1) as eluent (636 mg, 80% yield). Mp: 74–76 °C; $[\alpha]^{25}_D$ = –50.6 (c 1.0, $CHCl_3$); ^{1}H NMR (300 MHz, $CDCl_3$): δ (ppm) 8.69 (s, 1H), 7.24–7.13 (m, 1H), 1.20 (s, 9H); ^{19}F NMR (282 MHz, $CDCl_3$): δ (ppm) -138.03 – -138.17 (m, 2F), -141.02 – -141.16 (m, 2F); ^{13}C {^{1}H} NMR (75 MHz, $CDCl_3$): δ (ppm) 151.9, 147.9–144.19 (m, 2C–F), 147.3–143.7 (m, 2C–F), 114.4 (t, *J* = 10.8 Hz), 109.1 (t, *J* = 22.6 Hz), 58.4, 22.4. HRMS (ESI) m/z: [M + H$^+$] Calcd for $C_{11}H_{12}F_4NOS$ 282.0579; Found 282.0570.

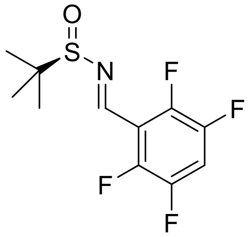

(*R*,*E*)-2-Methyl-*N*-(2,4,6-trifluorobenzylidene)propane-2-sulfinamide (1c). According to general procedure, from 500 mg (3.12 mmol) of 2,4,6-trifluorobenzaldehyde, compound **1c** was obtained as a colorless oil after column chromatography on silica gel using *n*-hexane:EtOAc (4:1) as eluent (612 mg, 72% yield); $[\alpha]^{25}_D$ = –64.2 (c 1.0, $CHCl_3$); ^{1}H NMR (300 MHz, $CDCl_3$): δ (ppm) 8.68 (s, 1H), 6.76–6.68 (m, 2H), 1.22 (s, 9H); ^{19}F NMR (282 MHz, $CDCl_3$): δ (ppm) -99.93 (t, *J* = 9.7 Hz, 1F), -106.89 (d, *J* = 9.7 Hz, 2F); ^{13}C {^{1}H} NMR (75 MHz, $CDCl_3$): δ (ppm) 164.8 (dt, $^1J_{CF}$ = 257.0 Hz, $^3J_{CF}$ = 15.8 Hz), 162.7 (ddd, $^1J_{CF}$ = 257.0 Hz, $^3J_{CF}$ = 15.8 Hz, *J* = 8.5 Hz), 152.3, 101.3 (td, *J* = 25.7, 3.9 Hz), 58.0, 22.5. HRMS (ESI): m/z Calcd for $C_{11}H_{13}F_3NOS$ [M+H$^+$]: 264.0661; Found 264.0664.

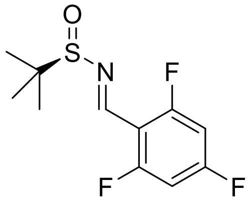

(*R*,*E*)-*N*-(2,6-Difluorobenzylidene)-2-methylpropane-2-sulfinamide (1d). According to general

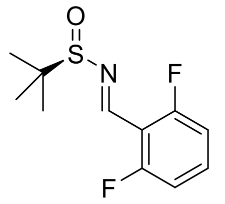

procedure, from 500 mg (3.52 mmol) of 2,6-difluorobenzaldehyde, compound **1d** was obtained as a white solid after column chromatography on silica gel using *n*-hexane:EtOAc (4:1) as eluent (783 mg, 91% yield). Mp: 49–51 °C; $[\alpha]^{25}_{D}$ = –61.7 (c 1.0, $CHCl_3$); ^{1}H NMR (300 MHz, $CDCl_3$): δ (ppm) 8.77 (s, 1H), 7.45–7.35 (m 1H), 6.98–6.91 (m, 2H), 1.23 (s, 9H); ^{19}F NMR (282 MHz, $CDCl_3$): δ (ppm) -110.72 (s, 2F); ^{13}C {^{1}H} NMR (75 MHz, $CDCl_3$): δ (ppm) 163.8 (d, $^{3}J_{CF}$ = 5.8 Hz), 160.3 (d, $^{3}J_{CF}$ = 5.8 Hz), 153.3, 133.7 (t, *J* = 11.0 Hz), 112.2 (d, *J* = 25.2 Hz), 58.0, 22.5. HRMS (ESI) m/z: [M + H^{+}] Calcd for $C_{11}H_{14}F_2NOS$ 246.0759; Found 246.0758.

(*R*,*E*)-*N*-(2-Fluorobenzylidene)-2-methylpropane-2-sulfinamide (1e). According to general

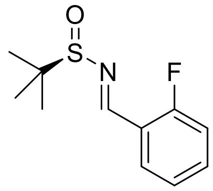

procedure, from 500 mg (4.03 mmol) of 2-fluorobenzaldehyde, compound **1e** was obtained as a colorless oil after column chromatography on silica gel using *n*-hexane:EtOAc (4:1) as eluent (611 mg, 67% yield); $[\alpha]^{25}_{D}$ = –78.3 (c 1.0, $CHCl_3$); ^{1}H NMR (300 MHz, $CDCl_3$): δ (ppm) 8.76 (s, 1H), 7.86 (td, *J* = 7.6, 1.8 Hz, 1H), 7.39-7.32 (m, 1H), 7.09 (t, *J* = 7.6 Hz, 1H), 7.03–6.97 (m, 1H), 1.13 (s, 9H); ^{19}F NMR (282 MHz, $CDCl_3$): δ (ppm) -118.18 (s, 1F); ^{13}C {^{1}H} NMR (75 MHz, $CDCl_3$): δ (ppm) 160.4 (d, $^{1}J_{CF}$ = 256.9 Hz), 156.4 (d, *J* = 5.4 Hz), 134.2 (d, *J* = 8.8 Hz), 128.6 (d, *J* = 2.0 Hz), 124.5 (d, *J* = 3.7 Hz), 122.0 (d, *J* = 9.4 Hz), 116.2 (d, *J* = 20.8 Hz), 57.8, 22.6. HRMS (ESI) m/z: [M + H^{+}] Calcd for $C_{11}H_{15}FNOS$ 228.0853; Found 228.0853.

(*R*,*E*)-*N*-benzylidene-2-methylpropane-2-sulfinamide (1f). Spectroscopic data of compound **1f** were in agreement with those previously reported.[1]

III. General procedure for the diastereoselective propargylation of sulfinyl imines.

III.a. General procedure for the propargylation reaction to sulfinamides 3 in THF.

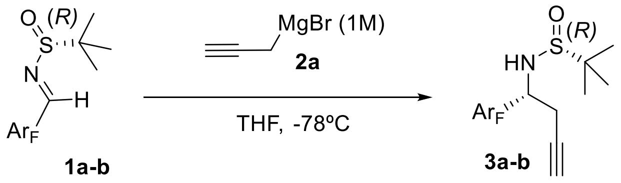

First, a 1 M solution of Grignard reagent in diethyl ether was prepared by adding magnesium turnings (214 mg, 11 mmol), mercury chloride (II) (19 mg, 1.7 mol%), two iodine balls and Et_2O (5 mL, 1 M) to a sealed tube under a nitrogen atmosphere. This mixture was cooled to 0 °C and propargyl bromide was added slowly (0.56 mL, 5 mmol). The mixture was then heated an oil

bath and stirred at 35 °C for 1.5 h. After this time, the mixture was cooled to room temperature, the stirring stopped, and the solution was used as a reagent in the next step without purification.

Next, for the asymmetric propargylation, a solution of the corresponding fluorinated imine **1** (1 mmol) in THF (0.1 M) was cooled to –78 °C. The freshly prepared Grignard reagent (1.5 mmol) was slowly added, and the reaction mixture was stirred at this temperature until the reaction was complete (TLC analysis, typically 24 h). The reaction mixture was then quenched with a saturated aqueous solution of NH_4Cl and extracted with EtOAc. The combined organic phases were dried over anhydrous Na_2SO_4, concentrated and the crude mixture was purified by flash column chromatography using deactivated silica gel (*n*-hexane:EtOAc).

(*RS*,*R*)-2-Methyl-*N*-(1-(perfluorophenyl)but-3-yn-1-yl)propane-2-sulfinamide (3a). According

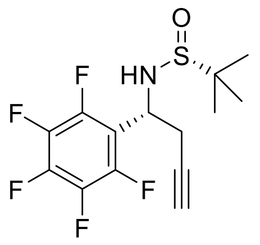

to general procedure, from 506 mg (0.91 mmol) of **1a**, compound **3a** was obtained as a yellowish oil after column chromatography on silica gel using *n*-hexane:EtOAc (6:1) as eluent (383 mg, 67% yield); $[\alpha]^{25}_D$ = +45.4 (c 1.0, $CHCl_3$); ^1H NMR (300 MHz, $CDCl_3$): δ (ppm) 4.89–4.80 (m, 1H), 3.99 (d, *J* = 10.6 Hz), 2.81 (ddd, *J* = 16.7, 6.8, 2.6 Hz, 1H), 2.71 (ddd, *J* = 16.7, 8.2, 2.6 Hz, 1H) 1.98 (t, *J* = 2.6 Hz, 1H), 1.18 (s, 9H); ^{19}F NMR (282 MHz, $CDCl_3$): δ (ppm) -142.77 – -142.90 (m, 2F), -154.15 – -154.30 (m, 1F), -161.19 – -161.38 (m, 2F); ^{13}C {^1H} NMR (75 MHz, $CDCl_3$): δ (ppm) 146.6–143.1 (m, 2C–F), 142.9–139.2 (m, 1C–F), 139.0–135.6 (m, 2C–F), 115.2–114.8 (m, 1C), 78.4, 71.6, 56.7, 51.0, 26.3, 22.3 HRMS (ESI) m/z: [M + H^+] Calcd for $C_{14}H_{15}F_5NOS$ 340.0790; Found 340.0791.

(*R$_S$*,*R*)-2-Methyl-*N*-(1-(2,3,5,6-tetrafluorophenyl)but-3-yn-1-yl)propane-2-sulfinamide (3b).

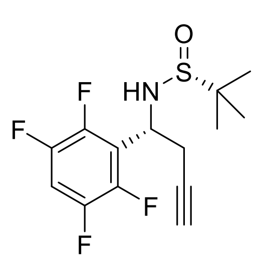

According to general procedure, from 103 mg (0.37 mmol) of **1b**, compound **3b** was obtained as a white solid after column chromatography on silica gel using *n*-hexane:EtOAc (6:1) as eluent (71 mg, 61% yield). Mp: 82–84 °C; $[\alpha]^{25}_D$ = +51.2 (c 1.0, $CHCl_3$); ^1H NMR (300 MHz, $CDCl_3$): δ (ppm) 7.07–6.96 (m, 1H), 4.97–4.88 (m, 1H), 4.04 (d, *J* = 10.5 Hz), 2.84 (ddd, *J* = 16.7, 7.0, 2.6 Hz, 1H), 2.74 (ddd, *J* = 16.7, 7.9, 2.6 Hz, 1H) 1.99 (t, *J* = 2.6 Hz, 1H), 1.22 (s, 9H); ^{19}F NMR (282 MHz, $CDCl_3$): δ (ppm) 138.15 – -138.27 (m, 2F), -143.32 – -143.45 (m, 2F); ^{13}C {^1H} NMR (75 MHz, $CDCl_3$): δ (ppm) 147.8–144.09 (m, 2C–F), 146.1–142.5 (m, 2C–F), 120.7 (t, *J* = 15.1 Hz, 1C), 105.9 (t, *J* = 22.6 Hz, 1C), 78.7, 71.4, 56.7, 51.4, 26.4, 22.4. HRMS (ESI) m/z: [M + H^+] Calcd for $C_{14}H_{16}F_4NOS$ 322.0879; Found 322.0883.

III.b. General procedure for the propargylation reaction to sulfinamides 3′ in DCM.

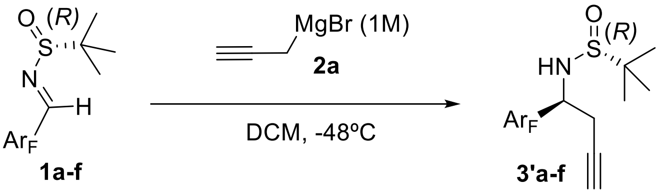

First, a 1 M solution of Grignard reagent in diethyl ether was prepared by adding magnesium turnings (214 mg, 11 mmol), mercury chloride (II) (19 mg, 1.7 mol%), two iodine balls and Et_2O (5 mL, 1 M) to a sealed tube under a nitrogen atmosphere. This mixture was cooled to 0 °C and propargyl bromide was added slowly (0.56 mL, 5 mmol). The mixture was then stirred at 35 °C for 1.5 h. After this time, the mixture was cooled to room temperature, the stirring stopped, and the solution was used as a reagent in the next step without purification.

Next, for the asymmetric propargylation, a solution of the corresponding fluorinated imine **1** (1 mmol) in DCM (0.1 M) was cooled to –48 °C. The freshly prepared Grignard reagent (1.5 mmol) was slowly added, and the reaction mixture was stirred at this temperature until the reaction was complete (TLC analysis, typically 18–24 h). The reaction mixture was then quenched with a saturated aqueous solution of NH_4Cl and extracted with EtOAc. The combined organic phases were dried over anhydrous Na_2SO_4, concentrated and the crude mixture was purified by flash column chromatography using deactivated silica gel (*n*-hexane:EtOAc).

(*R*~S~,*S*)-2-Methyl-*N*-(1-(perfluorophenyl)but-3-yn-1-yl)propane-2-sulfinamide (3′a). According to general procedure, from 51 mg (0.17 mmol) of **1a**, compound **3′a** was obtained as a yellowish solid after column chromatography on silica gel using *n*-hexane:EtOAc (6:1) as eluent (46 mg, 80% yield). Mp: 83–85 °C; $[\alpha]^{25}_{D}$ = –50.3 (c 1.0, $CHCl_3$); ^1H NMR (300 MHz, $CDCl_3$): δ (ppm) 7.08–6.97 (m, 1H), 4.99 (q, *J* = 7.8 Hz, 1H), 4.00 (d, *J* = 7.4 Hz), 2.97 (ddd, *J* = 16.6, 6.4, 2.6 Hz, 1H), 2.84 (ddd, *J* = 16.6, 8.2, 2.6 Hz, 1H) 2.04 (t, *J* = 2.6 Hz, 1H), 1.18 (s, 9H); ^{19}F NMR (282 MHz, $CDCl_3$): δ (ppm) -141.91 – -142.04 (m, 2F), -153.65 – -153.81 (m, 1F), -161.10 – -161.30 (m, 2F); ^{13}C {^1H} NMR (75 MHz, $CDCl_3$): δ (ppm) 146.8–143.1 (m, 2C–-F), 142.8–139.5 (m, 2C–F), 136.2–135.7 (m, 1C–F), 114.4–113.9 (m, 1C), 78.3, 72.1, 56.4, 50.9, 26.5, 22.3. HRMS (ESI) m/z: $[M + H^+]$ Calcd for $C_{14}H_{15}F_5NOS$ 340.0790; Found 340.0788.

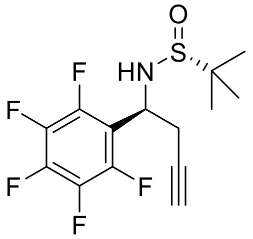

(R_S,S)-2-Methyl-*N*-(1-(2,3,5,6-tetrafluorophenyl)but-3-yn-1-yl)propane-2-sulfinamide (3’b).

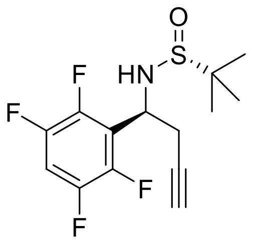

According to general procedure, from 517 mg (0.91 mmol) of **1b**, compound **3’b** was obtained as a white solid after column chromatography on silica gel using *n*-hexane:EtOAc (6:1) as eluent (405 mg, 68% yield). Mp: 58–60 °C; $[\alpha]^{25}_D$ = –49.4 (c 1.0, $CHCl_3$); ^1H NMR (300 MHz, $CDCl_3$): δ (ppm) 7.08–6.97 (m, 1H), 5.01 (q, *J* = 7.8 Hz, 1H), 4.04 (d, *J* = 7.8 Hz), 2.97 (ddd, *J* = 16.6, 6.4, 2.6 Hz, 1H), 2.84 (ddd, *J* = 16.6, 8.1, 2.6 Hz, 1H) 2.02 (t, *J* = 2.6 Hz, 1H), 1.16 (s, 9H); ^{19}F NMR (282 MHz, $CDCl_3$): δ (ppm) -138.28 – -138.41 (m, 2F), -142.64 – -142.77 (m, 2F); ^{13}C {^1H} NMR (75 MHz, $CDCl_3$): δ (ppm) 147.7–144.06 (m, 2C–F), 146.2-142.7 (m, 2C–F), 119.9 (t, *J* = 14.7 Hz, 1C), 105.8 (t, *J* = 22.6 Hz, 1C), 78.4, 71.9, 56.4, 51.3, 26.5, 22.3. HRMS (ESI) m/z: [M + H^+] Calcd for $C_{14}H_{16}F_4NOS$ 322.0879; Found 322.0883.

(R_S,S)-2-Methyl-*N*-(1-(2,4,6-trifluorophenyl)but-3-yn-1-yl)propane-2-sulfinamide (3’c).

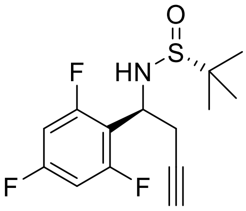

According to general procedure, from 282 mg (0.91 mmol) of **1c**, compound **3’c** was obtained as a colorless oil after column chromatography on silica gel using *n*-hexane:EtOAc (6:1) as eluent (290 mg, 89% yield); $[\alpha]^{25}_D$ = –53.4 (c 1.0, $CHCl_3$); ^1H NMR (300 MHz, $CDCl_3$): δ (ppm) 6.66–6.56 (m, 2H), 4.89 (q, *J* = 7.3 Hz, 1H), 3.97 (d, *J* = 7.3 Hz, 1H), 2.90 (ddd, *J* = 16.6, 6.8, 2.6 Hz, 1H), 2.75 (ddd, *J* = 16.6, 8.0, 2.6 Hz, 1H), 1.96 (t, *J* = 2.6 Hz, 1H), 1.10 (s, 9H); ^{19}F NMR (282 MHz, $CDCl_3$): δ (ppm) -107.66 (t, J_{FF} = 6.7 Hz, 1F), -110.12 (d, J_{FF} = 6.7 Hz, 2F); ^{13}C {^1H} NMR (75 MHz, $CDCl_3$): δ (ppm) 162.2 (dt, $^1J_{CF}$ = 250 Hz, $^3J_{CF}$ = 15.9 Hz, C-F), 161.2 (ddd, $^1J_{CF}$ = 250 Hz, $^3J_{CF}$ = 14.8, 11.0 Hz, C-F) 112.8 (td, *J* = 17.1, 4.9 Hz), 101.0–100.2 (m, 1C), 79.1, 71.5, 56.1, 50.3, 26.4, 22.3. HRMS (ESI) m/z: [M + H^+] Calcd for $C_{14}H_{17}F_3NOS$ 304.0974; Found 304.0977.

(R_S,S)-*N*-(1-(2,6-Difluorophenyl)but-3-yn-1-yl)-2-methylpropane-2-sulfinamide (3’d).

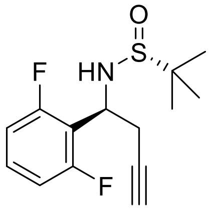

According to general procedure, from 223 mg (0.91 mmol) of **1d**, compound **3’d** was obtained as a white solid after column chromatography on silica gel using *n*-hexane:EtOAc (6:1) as eluent (179 mg, 70% yield). Mp: 70–72 °C; $[\alpha]^{25}_D$ = –44.9 (c 1.0, $CHCl_3$); ^1H NMR (300 MHz, $CDCl_3$): δ (ppm) 7.24–7.14 (m, 1H), 6.87–6.78 (m, 2H), 4.94 (dd, *J* = 14.8, 7.7 Hz, 1H), 4.00 (d, *J* = 7.7 Hz, 1H), 2.93 (ddd, *J* = 16.6, 6.7, 2.6 Hz, 1H), 2.77 (ddd, *J* = 16.6, 8.0, 2.6 Hz, 1H), 1.93 (t, *J* = 2.6 Hz, 1H), 1.09 (s, 9H); ^{19}F NMR (282 MHz, $CDCl_3$): δ (ppm) -113.52 (s, 2F); ^{13}C {^1H} NMR (75 MHz, $CDCl_3$): δ (ppm) 161.0 (d, $^1J_{CF}$ = 248.7 Hz, $^3J_{CF}$ = 8.1 Hz), 129.8 (t, *J* = 10.7 Hz), 116.5 (t, *J* = 16.7 Hz), 111.8 (d, *J* = 26.2 Hz), 79.3, 71.3, 56.1, 50.8, 26.6, 22.3. HRMS (ESI) m/z: [M + H^+] Calcd for $C_{14}H_{17}F_2NOS$ 286.1072; Found 286.1073.

(R_S,S)-*N*-(1-(2-Fluorophenyl)but-3-yn-1-yl)-2-methylpropane-2-sulfinamide (3’e). According to general procedure, from 74 mg (0.33 mmol) of **1e**, compound **3’e** was obtained as a white solid after column chromatography on silica gel using *n*-hexane:EtOAc (6:1) as eluent (76 mg, 86% yield). Mp: 100–102 °C; $[\alpha]^{25}_D$ = –47.6 (c 1.0, $CHCl_3$); ^{1}H NMR (300 MHz, $CDCl_3$): δ (ppm) 7.31 (td, *J* = 7.5, 1.2 Hz, 1H), 7.25-7.17 (m, 1H), 7.06 (td, *J* = 7.5, 1.2 Hz, 1H), 6.97 (ddd, *J* = 10.6, 8.2, 1.2 Hz, 1H), 4.81 (dd, *J* = 12.2, 5.1 Hz, 1H), 3.95 (d, *J* = 5.1 Hz, 1H), 2.82–2.64 (m, 2H), 2.02 (t, *J* = 2.6 Hz, 1H), 1.15 (s, 9H); ^{19}F NMR (282 MHz, $CDCl_3$): δ (ppm) -117.85 (s, 1F); ^{13}C {^{1}H} NMR (75 MHz, $CDCl_3$): δ (ppm) 160.4 (d, $^1J_{CF}$ = 247.3 Hz), 129.5 (d, *J* = 8.4 Hz), 128.7 (d, *J* = 4.1 Hz), 127.6 (d, *J* = 12.5 Hz), 124.1 (d, *J* =3.5 Hz), 115.7 (d, *J* = 21.8 Hz), 79.5, 72.2, 56.0, 51.9, 27.3, 22.5. HRMS (ESI) m/z: [M + H$^+$] Calcd for $C_{14}H_{18}FNOS$ 268.1166; Found 268.1163.

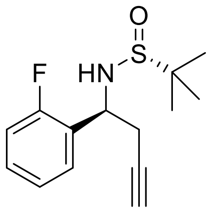

(R_S,S)-*N*-(1-(Phenyl)but-3-yn-1-yl)-2-methylpropane-2-sulfinamide (3’f). Spectroscopic data of compound **3’f** were in agreement with those previously reported.[1]

IV. General procedure for the propargylation reaction in DCM.

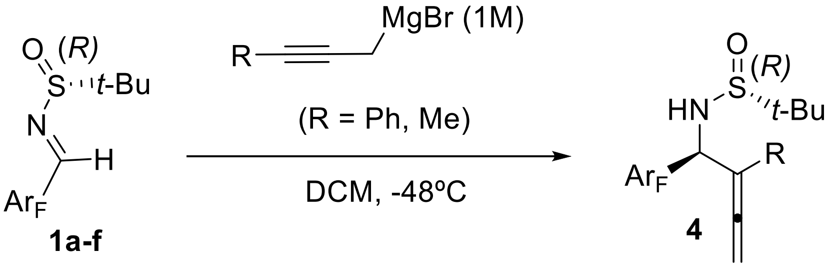

1a-f **4**

First, a 1 M solution of Grignard reagent in diethyl ether was prepared by adding magnesium turnings (214 mg, 11 mmol), mercury chloride (II) (19 mg, 1.7 mol%), two iodine balls and Et_2O (5 mL, 1 M) to a sealed tube under a nitrogen atmosphere. This mixture was cooled to 0 °C and the corresponding bromide was added slowly (0.56 mL, 5 mmol). The mixture was then heated an oil bath and stirred at 35 °C for 1.5 h. After this time, the mixture was cooled to room temperature, the stirring stopped, and the solution was used as a reagent in the next step without purification.

For the next asymmetric propargylation, a solution of the corresponding fluorinated imine **1** (1 mmol) in DCM (0.1 M) was cooled to –48 °C. The freshly prepared Grignard reagent (1.5 mmol) was slowly added, and the reaction mixture was stirred at this temperature until the reaction was complete (TLC analysis, typically 18–24 h). The reaction mixture was then quenched with a saturated aqueous solution of NH_4Cl and extracted with EtOAc. The combined

organic phases were dried over anhydrous Na_2SO_4, concentrated and the crude mixture was purified by flash column chromatography using deactivated silica gel (*n*-hexane:EtOAc).

(*R*$_S$,*S*)-2-Methyl-*N*-(1-(perfluorophenyl)-2-phenyl-3λ^5-buta-2,3-dien-1-yl)propane-2-sulfinamide (4ab).

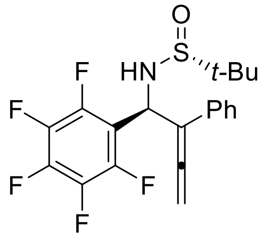

According to general procedure from 51 mg (0.17 mmol) of **1a**, compound **4ab** was obtained as a colorless oil after column chromatography on silica gel using *n*-hexane:EtOAc (6:1) as eluent (51 mg, 72% yield); $[\alpha]^{25}_D$ = –90.9 (c 1.0, $CHCl_3$); ^{1}H NMR (300 MHz, $CDCl_3$): δ (ppm) 7.38–7.29 (m, 5H), 5.95–5.92 (m, 1H), 5.44–5.32 (m, 2H), 4.19 (d, *J* = 4.8 Hz, 1H), 1.17 (s, 9H); ^{19}F NMR (282 MHz, $CDCl_3$): δ (ppm) -141.48 – -141.58 (m, 2F), -147.20 – -153.90 (t, *J* = 21.0 Hz, 1F), -161.46 – -161.64 (m, 2F); ^{13}C {^{1}H} NMR (75 MHz, $CDCl_3$): δ (ppm) 207.4, 147.6–144.0 (m, 2C–F), 146.5–143.0 (m, 2C-F), 132.9, 128.8, 127.8, 126.6, 120.5 (t, *J* = 13.8 Hz, C–F), 106.6, 105.7 (t, *J* = 22.6 Hz, C), 83.0, 56.5, 49.0, 22.4. HRMS (ESI) m/z: [M + H$^+$] Calcd for $C_{20}H_{19}F_5NOS$ 416.1099; Found 416.1102.

(*R*$_S$,*S*)-2-Methyl-*N*-(2-methyl-1-(perfluorophenyl)-3λ^5-buta-2,3-dien-1-yl)propane-2-sulfinamide (4ac). According to general procedure from 53 mg (0.18 mmol) of **1a**, compound

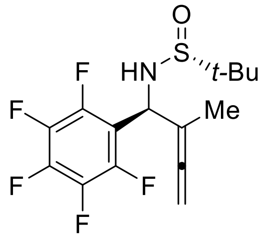

4ac was obtained as a colorless oil after column chromatography on silica gel using *n*-hexane:EtOAc (6:1) as eluent (54 mg, 85% yield); $[\alpha]^{25}_D$ = –93.1 (c 1.0, $CHCl_3$); ^{1}H NMR (300 MHz, $CDCl_3$): δ (ppm) 5.20–5.15 (m, 1H), 4.99–4.86 (m, 2H), 4.09 (d, *J* = 6.4 Hz, 1H), 1.73 (t, *J* = 3.1 Hz, 3H), 1.16 (s, 9H); ^{19}F NMR (282 MHz, $CDCl_3$): δ (ppm) -142.44 – -142.56 (m, 2F), -147.20 – -154.45 – -154.60 (m, 1F), -161.58 – -161.76 (m, 2F); ^{13}C {^{1}H} NMR (75 MHz, $CDCl_3$): δ (ppm) 205.0, 146.7–143.0 (m , 2C-F), 142.6–142.1 (m, 1C–F), 139.4–135.6 (m, 2C–F), 115.1–114.6 (m ,1C), 99.0, 79.9, 56.4, 52.4, 22.4, 16.2. HRMS (ESI) m/z: [M + H$^+$] Calcd for $C_{15}H_{17}F_5NOS$ 354.0943; Found 354.0946.

(*R*$_S$,*S*)-2-Methyl-*N*-(2-phenyl-1-(2,3,5,6-tetrafluorophenyl)-3λ^5-buta-2,3-dien-1-yl)propane-2-sulfinamide (4bb).

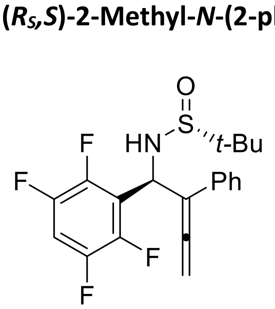

According to general procedure from 50 mg (0.18 mmol) of **1b**, compound **4bb** was obtained as a colorless oil after column chromatography on silica gel using *n*-hexane:EtOAc (6:1) as eluent (48 mg, 68% yield); $[\alpha]^{25}_D$ = –61.6 (c 1.0, $CHCl_3$); ^{1}H NMR (300 MHz, $CDCl_3$): δ (ppm) 7.39–7.28 (m, 5H), 7.01–6.90 (m, 1H), 5.98–5.94 (m, 1H), 5.44–5.32 (m, 2H), 4.22 (d, *J* = 5.3 Hz, 1H), 1.17 (s, 9H); ^{19}F NMR (282 MHz, $CDCl_3$): δ (ppm) -138.64 – -138.76 (m, 2F), -142.19 – -142.32 (m, 2F); ^{13}C {^{1}H} NMR (75 MHz, $CDCl_3$): δ (ppm) 207.3, 147.7–144.0 (m, 2C–F), 146.4–143.0 (m, 2C–F), 132.9, 128.8, 127.8, 126.6, 120.5

(t, J = 13.8 Hz, C), 106.6, 105.7 (t, J = 22.6 Hz, CH), 83.0, 56.5, 49.0, 22.4. HRMS (ESI) m/z: [M + H^+] Calcd for $C_{20}H_{20}F_4NOS$ 398.1203; Found398.1196.

(*R*$_S$,*S*)-2-Methyl-*N*-(2-methyl-1-(2,3,5,6-tetrafluorophenyl)-3λ^5-buta-2,3-dien-1-yl)propane-2-

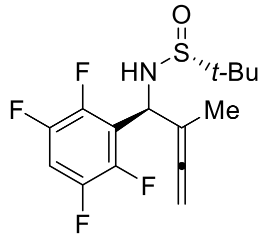

sulfinamide (4bc). According to general procedure from 50 mg (0.18 mmol) of **1b**, compound **4bc** was obtained as a colorless oil after column chromatography on silica gel using *n*-hexane:EtOAc (6:1) as eluent (60 mg, 88% yield); $[\alpha]^{25}_D$ = –79.4 (c 1.0, $CHCl_3$); ^{1}H NMR (300 MHz, $CDCl_3$): δ (ppm) 7.06–6.95 (m, 1H), 5.23–5.19 (m, 1H), 4.98–4.86 (m, 2H), 4.15 (d, J = 6.9 Hz, 1H), 1.73 (t, J = 3.1 Hz, 3H), 1.15 (s, 9H); ^{19}F NMR (282 MHz, $CDCl_3$): δ (ppm) -138.70 – -138.83 (m, 2F), -143.09 – -143.21 (m, 2F); ^{13}C {^{1}H} NMR (75 MHz, $CDCl_3$): δ (ppm) 205.1, 147.1–144.4 (m, 2C–F), 145.8–143.1 (m, 2C–F), 120.7 (t, J = 14.4 Hz, C), 105.3, 99.1, 79.8, 56.4, 52.8, 22.4, 16.2. HRMS (ESI) m/z: [M + H^+] Calcd for $C_{15}H_{18}F_4NOS$ 336.1037; Found 336.1040.

(*R*$_S$,*S*)-2-Methyl-*N*-(2-phenyl-1-(2,4,6-trifluorophenyl)-3λ^5-buta-2,3-dien-1-yl)propane-2-

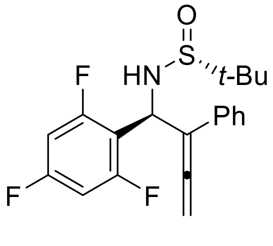

sulfinamide (4cb). According to general procedure, from 55 mg (0.19 mmol) of **1c**, compound **4cb** was obtained as a colorless oil after column chromatography on silica gel using *n*-hexane:EtOAc (6:1) as eluent (53 mg, 72% yield); $[\alpha]^{25}_D$ = –96.4 (c 1.0, $CHCl_3$); ^{1}H NMR (300 MHz, $CDCl_3$): δ (ppm) 7.38–7.26 (m, 5H), 6.61–6.55 (m, 2H), 5.91–5.86 (m, 1H), 5.39–5.28 (m, 2H), 4.16 (d, J = 5.1 Hz, 1H), 1.15 (s, 9H); ^{19}F NMR (282 MHz, $CDCl_3$): δ (ppm) -107.78 (t, J_{FF} = 6.9 Hz, 1F), -109.61 (d, J_{FF} = 6.2 Hz, 2F); ^{13}C {^{1}H} NMR (75 MHz, $CDCl_3$): δ (ppm) 207.5, 162.2 (dt, $^1J_{CF}$ = 250 Hz, $^3J_{CF}$ = 15.8 Hz, C–F), 161.4 (ddd, $^1J_{CF}$ = 250 Hz, $^3J_{CF}$ = 14.8, 10.6 Hz, C–F), 131.7, 128.6, 127.6, 126.6, 113.3 (td, J = 16.0, 4.9 Hz), 107.1, 101.0–100.2 (m, 1C), 82.5, 56.3, 48.1, 22.4. HRMS (ESI) m/z: [M + H^+] Calcd for $C_{20}H_{21}F_3NOS$ 380.1298; Found 380.1290.

(*R*$_S$,*S*)-2-Methyl-*N*-(2-methyl-1-(2,4,6-trifluorophenyl)-3λ^5-buta-2,3-dien-1-yl)propane-2-

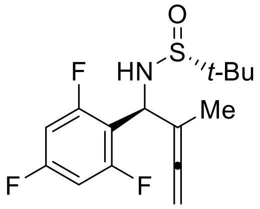

sulfinamide (4cc). According to general procedure, from 53 mg (0.20 mmol) of **1c**, compound **4cc** was obtained as a colorless oil after column chromatography on silica gel using *n*-hexane:EtOAc (6:1) as eluent (43 mg, 68% yield); $[\alpha]^{25}_D$ = –153.1 (c 1.0, $CHCl_3$); ^{1}H NMR (300 MHz, $CDCl_3$): δ (ppm) 6.68–6.62 (m, 2H), 5.18–5.13 (m, 1H), 4.96–4.85 (m, 2H), 4.12 (d, J = 6.5 Hz, 1H), 1.71 (t, J = 3.1 Hz, 3H), 1.15 (s, 9H); ^{19}F NMR (282 MHz, $CDCl_3$): δ (ppm) -108.31 (t, J_{FF} = 6.6 Hz, 1F), -110.59 (d, J_{FF} = 6.6 Hz, 2F); ^{13}C {^{1}H} NMR (75 MHz, $CDCl_3$): δ (ppm) 205.0,

162.0 (dt, $^{1}J_{CF}$ = 250 Hz, $^{3}J_{CF}$ = 15.8 Hz, C–F), 161.2 (ddd, $^{1}J_{CF}$ = 250 Hz, $^{3}J_{CF}$ = 14.8, 10.8 Hz, C–F), 113.4 (td, *J* = 16.6, 4.9 Hz), 100.9–100.2 (m, 1C), 99.7, 79.4, 56.2, 51.7, 22.4, 16.2. HRMS (ESI) m/z: [M + H$^+$] Calcd for $C_{15}H_{19}F_3NOS$ 318.1135; Found 318.1134.

(*R*$_S$,*S*)-*N*-(1-(2,6-Difluorophenyl)-2-phenyl-3λ^5-buta-2,3-dien-1-yl)-2-methylpropane-2-

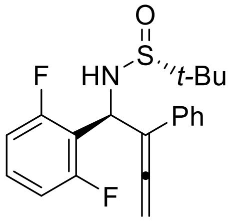

sulfinamide (4db). According to general procedure, from 55 mg (0.22 mmol) of **1d**, compound **4db** was obtained as a colorless oil after column chromatography on silica gel using *n*-hexane:EtOAc (6:1) as eluent (59 mg, 73% yield); $[\alpha]^{25}_{D}$ = –96.5 (c 1.0, $CHCl_3$); ^{1}H NMR (300 MHz, $CDCl_3$): δ (ppm) 7.38–7.23 (m, 5H), 7.20–7.12 (m, 1H), 6.82–6.76 (m, 2H), 5.94–5.90 (m, 1H), 5.38–5.26 (m, 2H), 4.21 (d, *J* = 5.8 Hz, 1H), 1.13 (s, 9H); ^{19}F NMR (282 MHz, $CDCl_3$): δ (ppm) -112.94 (s, 2F); ^{13}C {^{1}H} NMR (75 MHz, $CDCl_3$): δ (ppm) 207.6, 161.2 (dd, *J* = 250, 7.8 Hz, 2C–F), 133.5, 131.7, 129.6 (t, *J* = 10.7 Hz, 1C), 128.5, 127.4, 126.7, 117.0 (t, *J* = 15.8 Hz, C-F), 111.7 (d, *J* = 26.0 Hz, CH), 82.5, 56.3, 48.6, 22.4. HRMS (ESI) m/z: [M + H$^+$] Calcd for $C_{20}H_{22}F_2NOS$ 362.1380; Found 362.1385.

(*R*$_S$,*S*)-*N*-(1-(2,6-Difluorophenyl)-2-methyl-3λ^5-buta-2,3-dien-1-yl)-2-methylpropane-2-

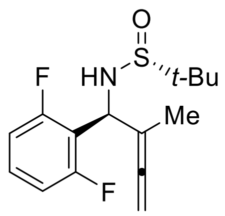

sulfinamide (4dc). According to general procedure, from 60 mg (0.24 mmol) of **1d**, compound **4dc** was obtained as a colorless oil after column chromatography on silica gel using *n*-hexane:EtOAc (6:1) as eluent (51 mg, 70% yield); $[\alpha]^{25}_{D}$ = –128.7 (c 1.0, $CHCl_3$); ^{1}H NMR (300 MHz, $CDCl_3$): δ (ppm) 7.24–7.18 (m, 1H), 6.88–6.82 (m, 2H), 5.22–5.17 (m, 1H), 4.94–4.82 (m, 2H), 4.17 (d, *J* = 7.1 Hz, 1H), 1.69 (t, *J* = 3.1 Hz, 3H), 1.13 (s, 9H); ^{19}F NMR (282 MHz, $CDCl_3$): δ (ppm) -113.89 (s, 2F); ^{13}C {^{1}H} NMR (75 MHz, $CDCl_3$): δ (ppm) 205.0, 161.0 (dd, *J* = 250, 8.0 Hz, 2C–F), 129.3 (t, *J* = 10.6 Hz, 1C), 117.1 (t, *J* = 16.3 Hz, C-F), 111.6 (d, *J* = 26.0 Hz, CH), 99.9, 79.2, 56.2, 52.2, 22.4, 16.2. HRMS (ESI) m/z: [M + H$^+$] Calcd for $C_{15}H_{20}F_2NOS$ 300.1228; Found 300.1228.

(*R*$_S$,*S*)-*N*-(1-(2-Fluorophenyl)-2-phenyl-3λ^5-buta-2,3-dien-1-yl)-2-methylpropane-2-

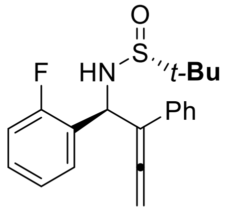

sulfinamide (4eb). According to general procedure, from 51 mg (0.22 mmol) of **1e**, compound **4eb** was obtained as a colorless oil after column chromatography on silica gel using *n*-hexane:EtOAc (10:1) as eluent (46 mg, 61% yield); $[\alpha]^{25}_{D}$ = –81.7 (c 1.0, $CHCl_3$); ^{1}H NMR (300 MHz, $CDCl_3$): δ (ppm) 7.33–7.29 (m, 2H), 7.23–7.08 (m, 5H), 7.02–6.92 (m, 2H), 5.79–5.75 (m, 1H), 5.31–5.18 (m, 2H), 3.83 (d, *J* = 4.3 Hz, 1H), 1.10 (s, 9H); ^{19}F NMR (282 MHz, $CDCl_3$): δ (ppm) -117.63 (s, 1F); ^{13}C {^{1}H} NMR (75 MHz, $CDCl_3$): δ (ppm) 208.4, 160.8 (d, *J* = 250 Hz, C–F), 133.5, 129.5 (d, *J* = 8.4

Hz), 129.4 (d, *J* = 3.6 Hz), 128.6, 127.7 (d, *J* = 12.8 Hz), 127.4, 126.6, 124.1 (d, *J* = 3.6 Hz), 115.5 (d, *J* = 21.9 Hz), 108.4, 82.5, 56.2, 51.0, 22.5. HRMS (ESI) m/z: [M + H^+] Calcd for $C_{20}H_{23}FNOS$ 344.1475; Found 344.1479.

(R_S,*S*)-*N*-(1-(2-Fluorophenyl)-2-methyl-3λ^5-buta-2,3-dien-1-yl)-2-methylpropane-2-sulfinamide (4ec).

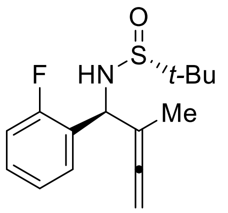

According to general procedure, from 53 mg (0.22 mmol) of **1e**, compound **4ec** was obtained as a colorless oil after column chromatography on silica gel using *n*-hexane:EtOAc (10:1) as eluent (55 mg, 84% yield); $[\alpha]^{25}_D$ = –133.4 (c 1.0, $CHCl_3$); ^{1}H NMR (300 MHz, $CDCl_3$): δ (ppm) 7.34–7.22 (m, 2H), 7.14–7.00 (m, 2H), 5.12–5.08 (m, 1H), 4.98–4.88 (m, 2H), 3.88 (d, *J* = 3.7 Hz, 1H), 1.63 (t, *J* = 3.1 Hz, 3H), 1.16 (s, 9H); ^{19}F NMR (282 MHz, $CDCl_3$): δ (ppm) -118.09 – -118.17 (m, 1F); ^{13}C {^{1}H} NMR (75 MHz, $CDCl_3$): δ (ppm) 204.9, 161.0 (d, *J* = 250 Hz, C–F), 129.4 (d, *J* = 7.1 Hz), 129.3 (d, *J* = 2.6 Hz), 127.6 (d, *J* = 12.7 Hz), 124.1 (d, *J* = 3.6 Hz), 115.5 (d, *J* = 22.0 Hz), 101.4, 79.2, 56.1, 53.4, 22.6, 16.3. HRMS (ESI) m/z: [M + H^+] Calcd for $C_{15}H_{21}FNOS$ 282.1324; Found 282.1322.

(R_S,*R*)-*N*-(1,2-Diphenyl-3λ^5-buta-2,3-dien-1-yl)-2-methylpropane-2-sulfinamide (4fb).

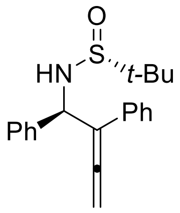

According to general procedure, from 55 mg (0.26 mmol) of **1f**, compound **4fb** was obtained as a colorless oil after column chromatography on silica gel using *n*-hexane:EtOAc (10:1) as eluent (44 mg, 52% yield); $[\alpha]^{25}_D$ = –124.4 (c 1.0, $CHCl_3$); ^{1}H NMR (300 MHz, $CDCl_3$): δ (ppm) 7.36–7.10 (m, 10H), 5.41–5.38 (m, 1H), 5.29–5.17 (m, 2H), 3.88 (d, *J* = 3.5 Hz, 1H), 1.11 (s, 9H); ^{13}C {^{1}H} NMR (75 MHz, $CDCl_3$): δ (ppm) 208.2, 140.2, 133.8, 128.5, 128.5, 128.4, 127.9, 127.3, 126.8, 109.4, 81.9, 57.2, 56.1, 22.6. HRMS (ESI) m/z: [M + H^+] Calcd for $C_{20}H_{24}NOS$ 326.1572; Found 326.1573.

(R_S,*R*)-2-Methyl-*N*-(2-methyl-1-phenyl-3λ^5-buta-2,3-dien-1-yl)propane-2-sulfinamide (4fc).

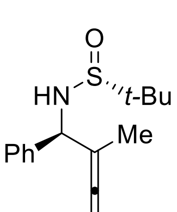

According to general procedure, from 76 mg (0.36 mmol) of **1f**, compound **4fc** was obtained as a colorless oil after column chromatography on silica gel using *n*-hexane:EtOAc (10:1) as eluent (64 mg, 67% yield); $[\alpha]^{25}_D$ = –128.5 (c 1.0, $CHCl_3$); ^{1}H NMR (300 MHz, $CDCl_3$): δ (ppm) 7.28–7.23 (m, 5H), 4.91–4.87 (m, 2H), 4.68–4.65 (m, 1H), 3.83 (d, *J* = 2.0 Hz, 1H), 1.50 (t, *J* = 3.0 Hz, 3H), 1.11 (s, 9H); ^{13}C {^{1}H} NMR (75 MHz, $CDCl_3$): δ (ppm) 204.5, 140.0, 128.4, 128.3, 128.0, 102.1, 78.7, 59.3, 55.9, 22.6, 16.2. HRMS (ESI) m/z: [M + H^+] Calcd for $C_{15}H_{22}NOS$ 264.1416; Found 264.1417.

V. X-ray structure of compound 3b (Deposition Number 2067822).

Experimental

Single crystals of $C_{14}H_{15}F_4NOS$ [**CCDC 2067822**] were obtained by slow evaporation method at room temperature using chloroform as solvent. A suitable crystal was selected and mounted on a SuperNova, Single source at offset, Atlas diffractometer. The crystal was kept at 150.00(10) K during data collection. Using Olex2,[2] the structure was solved with the ShelXS[3] structure solution program using Direct Methods and refined with the ShelXL[4] refinement package using Least Squares minimization. Displacement ellipsoids are drawn at the 50% probability level.

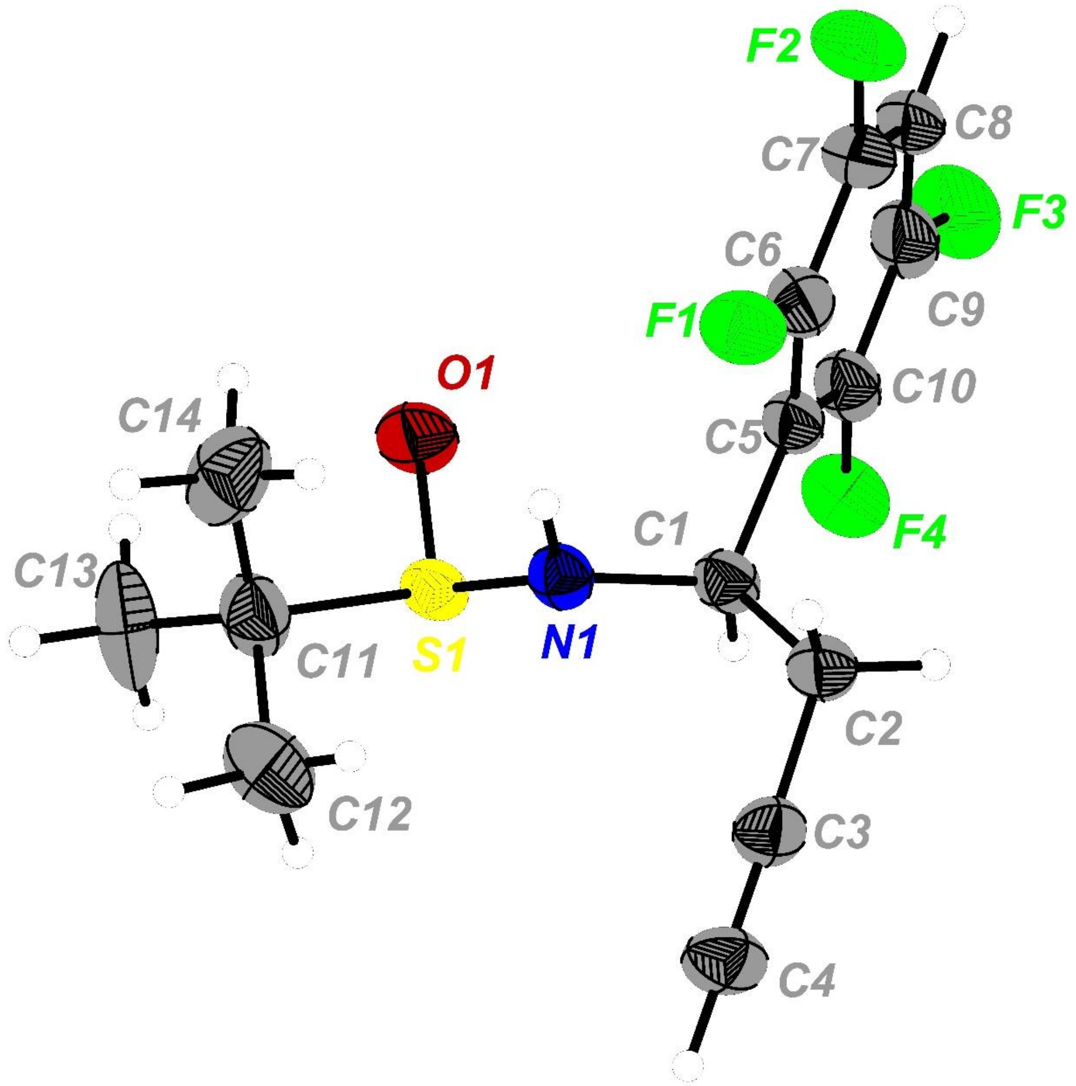

Table S1. Crystal data and structure refinement for CCDC 2067822.

Identification code	CCDC 2067822
Empirical formula	$C_{14}H_{15}F_4NOS$
Formula weight	321.33
Temperature/K	150.4(5)
Crystal system	monoclinic
Space group	$P2_1$
a/Å	7.8963(3)
b/Å	10.1860(3)
c/Å	10.2163(4)
α/°	90.0
β/°	107.419(4)
γ/°	90.0
Volume/Å^3	784.04(5)
Z	2
ρ_{calc}g/cm^3	1.361
μ/mm^{-1}	2.221
F(000)	332.0
Crystal size/mm^3	0.343 × 0.272 × 0.114
Radiation	CuKα (λ = 1.54184)
2Θ range for data collection/°	9.072 to 137.984
Index ranges	$-9 \le h \le 9$, $-12 \le k \le 12$, $-12 \le l \le 11$
Reflections collected	14341
Independent reflections	2909 [R_{int} = 0.0352, R_{sigma} = 0.0300]
Data/restraints/parameters	2909/2/196
Goodness-of-fit on F^2	1.046
Final R indexes [I>=2σ (I)]	R_1 = 0.0378, wR_2 = 0.0932
Final R indexes [all data]	R_1 = 0.0431, wR_2 = 0.0977
Largest diff. peak/hole / e Å^{-3}	0.21/-0.40
Flack parameter	-0.007(10)
Friedel coverage	99%
Flack x	-0.007(10)
Hooft y	-0.013(5)
P2(wrong)	$<10^{-99}$

Table S2. Fractional Atomic Coordinates (×10^4) and Equivalent Isotropic Displacement Parameters (Å^2×10^3) for CCDC 2067822. U_{eq} is defined as 1/3 of the trace of the orthogonalised U_{IJ} tensor.

Atom	*x*	*y*	*z*	U(eq)
S1	3522.0(11)	6708.1(9)	2607.7(8)	33.6(2)
F4	4687(3)	5356(2)	6429(2)	50.3(6)
F1	2501(3)	2462(2)	2698(2)	48.8(6)
F2	248(3)	1452(3)	3927(3)	66.0(8)
F3	2387(4)	4366(3)	7621(3)	62.5(8)
O1	1894(3)	6848(3)	3034(3)	45.9(7)
N1	4058(4)	5140(3)	2522(3)	33.4(7)
C5	3694(5)	3926(4)	4536(4)	33.8(8)
C10	3599(6)	4386(4)	5788(4)	39.1(9)
C1	4966(5)	4525(4)	3852(4)	34.1(8)
C3	7718(5)	4199(4)	3220(5)	39.9(9)
C6	2532(5)	2917(4)	3947(4)	39.5(9)
C9	2411(6)	3876(4)	6411(4)	46.0(11)
C7	1363(5)	2402(4)	4579(5)	45.8(10)
C11	2858(6)	7089(4)	767(4)	48.6(11)
C8	1272(5)	2878(4)	5804(5)	47.7(11)
C2	6338(5)	3519(4)	3664(4)	39.2(9)
C4	8790(6)	4789(4)	2885(5)	46.5(10)
C14	1323(7)	6221(6)	-14(5)	66.2(15)
C12	4480(7)	6912(6)	264(5)	69.5(15)
C13	2295(10)	8533(6)	710(6)	81.0(18)

Table S3. Anisotropic Displacement Parameters (Å^2×10^3) for CCDC 2067822. The Anisotropic displacement factor exponent takes the form: -2π^2[h^{2}a*^{2}U$_{11}$+2hka*b*U$_{12}$+…].

Atom	U_{11}	U_{22}	U_{33}	U_{23}	U_{13}	U_{12}
S1	34.7(4)	36.1(4)	34.8(4)	1.8(4)	17.5(3)	-0.4(4)
F4	64.6(15)	46.1(13)	45.6(14)	-8.6(11)	24.8(12)	-6.9(12)
F1	49.3(14)	52.3(14)	45.9(14)	-7.0(11)	15.7(11)	-10.5(11)
F2	55.8(15)	66.2(19)	74.6(18)	7.5(15)	17.3(13)	-25.9(14)
F3	77(2)	72.7(19)	53.9(16)	5.8(14)	43.4(15)	13.5(14)
O1	44.4(14)	52.7(16)	50.1(16)	1.9(15)	28.4(12)	5.7(13)
N1	36.3(16)	35.7(16)	30.7(16)	0.9(13)	13.9(13)	1.2(13)
C5	32.9(18)	35.1(18)	36.3(19)	6.8(16)	14.8(16)	4.6(15)
C10	43(2)	39(2)	41(2)	7.1(18)	21.2(19)	7.3(17)
C1	36(2)	35.6(18)	34.4(19)	2.2(15)	16.4(17)	0.1(15)
C3	37(2)	44(2)	41(2)	-0.3(18)	16.0(18)	4.3(17)
C6	41(2)	40(2)	39(2)	5.2(17)	14.1(17)	2.1(16)
C9	49(3)	52(2)	45(3)	14(2)	27(2)	15(2)
C7	36(2)	44(2)	57(3)	9(2)	15.3(19)	-4.5(18)
C11	57(3)	53(3)	40(2)	10.6(18)	20(2)	6.8(19)
C8	36(2)	58(3)	56(3)	23(2)	25(2)	8.8(18)
C2	36(2)	43(2)	43(2)	5.2(18)	17.7(18)	3.1(17)
C4	41(2)	50(2)	57(3)	-2(2)	27(2)	1.7(18)
C14	61(3)	89(4)	41(2)	-1(2)	4(2)	8(3)
C12	79(3)	95(4)	47(3)	14(3)	38(2)	1(3)
C13	119(5)	61(3)	58(3)	30(3)	19(3)	27(3)

Table S4. Bond Lengths for CCDC 2067822.

Atom	Atom	Length/Å	Atom	Atom	Length/Å
S1	O1	1.482(2)	C10	C9	1.383(5)
S1	N1	1.662(3)	C1	C2	1.544(5)
S1	C11	1.837(4)	C3	C2	1.472(5)
F4	C10	1.344(5)	C3	C4	1.170(6)
F1	C6	1.350(5)	C6	C7	1.379(5)
F2	C7	1.343(5)	C9	C8	1.376(6)
F3	C9	1.338(5)	C7	C8	1.364(6)
N1	C1	1.473(5)	C11	C14	1.520(7)
C5	C10	1.385(5)	C11	C12	1.526(6)
C5	C1	1.513(5)	C11	C13	1.532(7)
C5	C6	1.390(5)			

Table S5. Bond Angles for CCDC 2067822.

Atom	Atom	Atom	Angle/°	Atom	Atom	Atom	Angle/°
O1	S1	N1	111.37(16)	C7	C6	C5	121.8(4)
O1	S1	C11	105.90(18)	F3	C9	C10	119.0(4)
N1	S1	C11	98.53(17)	F3	C9	C8	120.3(4)
C1	N1	S1	114.9(2)	C8	C9	C10	120.8(4)
C10	C5	C1	121.2(4)	F2	C7	C6	118.3(4)
C10	C5	C6	116.0(3)	F2	C7	C8	120.3(4)
C6	C5	C1	122.8(3)	C8	C7	C6	121.4(4)
F4	C10	C5	119.6(3)	C14	C11	S1	110.7(3)
F4	C10	C9	118.4(3)	C14	C11	C12	111.9(4)
C9	C10	C5	122.0(4)	C14	C11	C13	111.2(4)
N1	C1	C5	112.9(3)	C12	C11	S1	108.0(3)
N1	C1	C2	109.5(3)	C12	C11	C13	111.3(4)
C5	C1	C2	112.2(3)	C13	C11	S1	103.4(3)
C4	C3	C2	177.1(5)	C7	C8	C9	118.0(4)
F1	C6	C5	119.1(3)	C3	C2	C1	109.7(3)
F1	C6	C7	119.1(4)				

Table S6. Torsion Angles for CCDC 2067822.

A	B	C	D	Angle/°	A	B	C	D	Angle/°
S1	N1	C1	C5	88.9(3)	C5	C1	C2	C3	-170.3(4)
S1	N1	C1	C2	-145.3(3)	C5	C6	C7	F2	178.2(4)
F4	C10	C9	F3	0.1(5)	C5	C6	C7	C8	1.2(6)
F4	C10	C9	C8	-179.7(4)	C10	C5	C1	N1	-117.2(4)
F1	C6	C7	F2	0.7(6)	C10	C5	C1	C2	118.5(4)
F1	C6	C7	C8	-176.3(4)	C10	C5	C6	F1	177.2(3)
F2	C7	C8	C9	-178.2(4)	C10	C5	C6	C7	-0.4(5)
F3	C9	C8	C7	-179.2(4)	C10	C9	C8	C7	0.5(6)
O1	S1	N1	C1	-79.2(3)	C1	C5	C10	F4	-1.9(6)
O1	S1	C11	C14	-55.3(4)	C1	C5	C10	C9	178.2(3)
O1	S1	C11	C12	-178.0(3)	C1	C5	C6	F1	-1.4(5)
O1	S1	C11	C13	63.9(4)	C1	C5	C6	C7	-178.9(4)
N1	S1	C11	C14	59.9(3)	C6	C5	C10	F4	179.6(3)
N1	S1	C11	C12	-62.8(4)	C6	C5	C10	C9	-0.4(6)
N1	S1	C11	C13	179.1(4)	C6	C5	C1	N1	61.2(4)
N1	C1	C2	C3	63.5(4)	C6	C5	C1	C2	-63.1(5)
C5	C10	C9	F3	-179.9(4)	C6	C7	C8	C9	-1.3(6)
C5	C10	C9	C8	0.3(6)	C11	S1	N1	C1	169.9(3)

Table S7. Hydrogen Atom Coordinates (Å×10[4]) and Isotropic Displacement Parameters (Å[2]×10[3]) for CCDC 2067822.

Atom	*x*	*y*	*z*	U(eq)
H1A	5638.4	5232.15	4471.87	41
H8	448.01	2530.47	6225.37	57
H2A	6888.9	3056.96	4540.74	47
H2B	5738.85	2859.15	2968.33	47
H4	9660.65	5267.34	2612.2	56
H14A	1730.49	5309.89	10.25	99
H14B	880.82	6517.29	-968.6	99
H14C	366.81	6275.22	413.08	99
H12A	5480.32	7409.25	857.29	104
H12B	4209.18	7234.84	-679.71	104
H12C	4792.5	5979.41	293.06	104
H13A	1300.73	8622.97	1092.41	121
H13B	1926.56	8833.03	-244.99	121
H13C	3295.96	9065.04	1246.76	121
H1	3250(60)	4590(50)	1970(50)	97

.

VI. X-ray structure of compound 3’b (Deposition Number 2067817).

Experimental

Single crystals of $C_{14}H_{15}F_4NOS$ [**CCDC 2067817**] were obtained by vapour diffusion method using dichloromethane and *n*-hexane (1:1) and slow evaporation in glass vial. A suitable crystal was selected and mounted on a SuperNova, Single source at offset, Atlas diffractometer. The crystal was kept at 150.00(10) K during data collection. Using Olex2,[2] the structure was solved with the ShelXS[3] structure solution program using Direct Methods and refined with the ShelXL[4] refinement package using Least Squares minimization. Displacement ellipsoids are drawn at the 50% probability level.

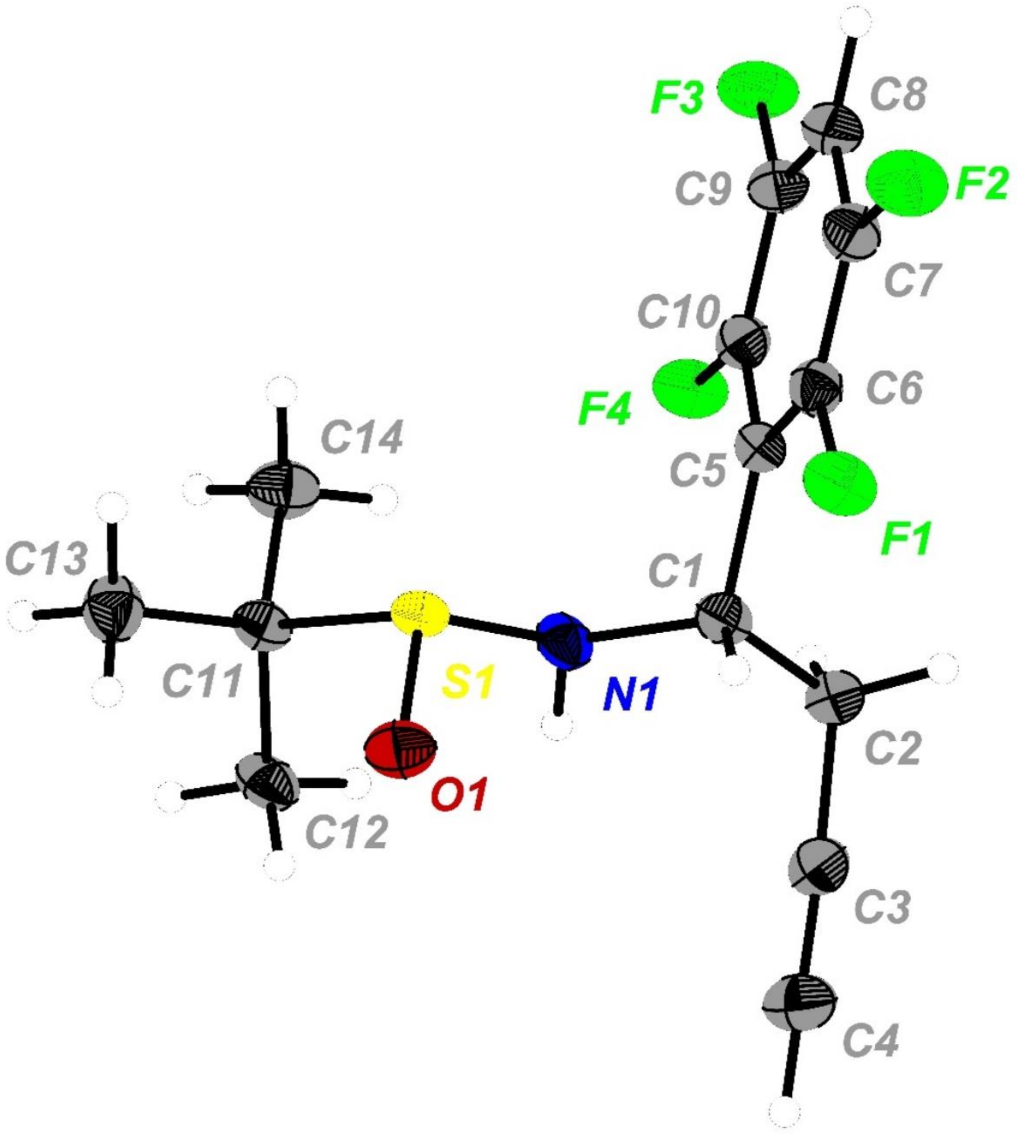

Table S8. Crystal data and structure refinement for CCDC 2067817.

Identification code	CCDC 2067817
Empirical formula	$C_{14}H_{15}F_4NOS$
Formula weight	321.33
Temperature/K	150.00(10)
Crystal system	monoclinic
Space group	$P2_1$
a/Å	8.87345(12)
b/Å	5.57753(8)
c/Å	15.18268(19)
α/°	90.0
β/°	101.7056(13)
γ/°	90.0
Volume/Å^3	735.793(18)
Z	2
ρ_{calc}g/cm^3	1.450
μ/mm^{-1}	2.372
F(000)	332.0
Crystal size/mm^3	0.297 × 0.165 × 0.067
Radiation	CuKα (λ = 1.54184)
2Θ range for data collection/°	10.18 to 137.99
Index ranges	$-10 \le h \le 10$, $-6 \le k \le 6$, $-18 \le l \le 18$
Reflections collected	13571
Independent reflections	2686 [R_{int} = 0.0408, R_{sigma} = 0.0263]
Data/restraints/parameters	2686/2/196
Goodness-of-fit on F^2	1.039
Final R indexes [I>=2σ (I)]	R_1 = 0.0330, wR_2 = 0.0877
Final R indexes [all data]	R_1 = 0.0342, wR_2 = 0.0891
Largest diff. peak/hole / e Å^{-3}	0.30/-0.16
Flack parameter	0.00(2)
Friedel coverage	99%
Flack x	-0.007(10)
Hooft y	-0.013(5)
P2(wrong)	$<10^{-99}$

Table S9. Fractional Atomic Coordinates (×10[4]) and Equivalent Isotropic Displacement Parameters (Å2×10[3]) for CCDC 2067817. Ueq is defined as 1/3 of the trace of the orthogonalised UIJ tensor.

Atom	x	y	z	U(eq)
S1	1263.3(7)	2075.0(13)	7843.6(3)	21.78(18)
F1	4575(2)	3228(4)	6268.6(11)	34.6(4)
F2	3720(2)	3291(4)	4482.7(12)	41.0(5)
F3	805(2)	-3727(4)	4576.9(12)	38.8(5)
F4	1643(2)	-3832(4)	6358.7(11)	32.2(4)
O1	2023(2)	3790(4)	8548.6(14)	32.2(5)
N1	2294(3)	-413(5)	7835.4(15)	25.9(5)
C1	3632(3)	-290(6)	7400.3(17)	24.4(6)
C5	3122(3)	-275(5)	6386.3(17)	22.4(6)
C6	3617(3)	1498(5)	5867.7(18)	24.7(6)
C7	3181(3)	1532(6)	4941.6(18)	28.5(7)
C8	2227(3)	-206(6)	4488.9(18)	30.0(6)
C9	1735(3)	-1983(6)	4989.3(18)	27.4(6)
C10	2172(3)	-2034(6)	5917.2(19)	23.9(6)
C2	4663(3)	-2477(6)	7726.4(18)	28.9(7)
C3	5386(3)	-2250(6)	8678(2)	32.1(7)
C4	5926(4)	-1967(7)	9452(2)	39.6(8)
C11	-322(3)	646(5)	8275.5(17)	23.2(6)
C12	298(4)	-538(6)	9182.5(18)	29.5(6)
C13	-1418(3)	2689(6)	8373(2)	32.4(7)
C14	-1090(4)	-1150(6)	7569(2)	32.0(7)

Table S10. Anisotropic Displacement Parameters (Å2×103) for CCDC 2067817. The Anisotropic displacement factor exponent takes the form: -2π2[h2a*2U11+2hka*b*U12+…].

Atom	U11	U22	U33	U23	U13	U12
S1	26.0(3)	21.8(3)	16.7(3)	1.2(2)	2.3(2)	-1.9(3)
F1	39.9(9)	33.8(10)	30.9(9)	-2.9(8)	9.1(7)	-13.8(8)
F2	46.7(11)	44.8(12)	31.9(9)	11.2(8)	8.8(8)	-11.4(9)
F3	41.3(10)	43.8(12)	28.5(9)	-6.7(8)	0.0(7)	-15.1(9)
F4	39.4(9)	29.7(9)	26.7(8)	1.0(7)	4.7(7)	-11.5(8)
O1	33.9(10)	30.5(13)	29.8(10)	-7.1(9)	0.4(8)	-6.0(9)
N1	31.4(12)	27.1(14)	21.0(11)	5.1(9)	9.5(9)	2.8(10)
C1	26.5(12)	26.7(16)	20.4(12)	-1.1(11)	5.5(9)	-3.4(11)
C5	21.2(11)	27.5(16)	18.7(12)	-0.2(11)	4.8(9)	1.3(10)
C6	23.0(12)	26.3(18)	25.6(13)	-1.1(10)	6.3(10)	-1.4(10)
C7	28.1(13)	33(2)	25.2(13)	7.1(11)	8.3(10)	1.2(11)
C8	29.0(13)	40.3(18)	20.3(13)	1.8(12)	4.0(10)	1.4(12)
C9	24.4(13)	32.0(17)	24.4(13)	-5.4(12)	1.7(10)	-2.8(11)
C10	22.9(12)	25.7(15)	23.2(13)	0.8(10)	4.4(10)	-0.4(10)
C2	24.6(12)	37(2)	24.3(12)	-0.9(12)	2.3(10)	1.2(12)
C3	25.5(13)	41(2)	28.9(15)	3.0(12)	3.1(11)	1.4(11)
C4	36.4(16)	52(2)	27.8(15)	4.0(14)	-0.1(12)	1.6(15)
C11	27.4(13)	22.7(15)	19.8(12)	0.5(10)	5.3(10)	-2.1(11)
C12	38.8(15)	31.2(16)	20.1(12)	4.7(11)	9.5(11)	2.0(13)
C13	32.4(14)	32(2)	34.6(14)	4.1(12)	10.2(11)	3.4(12)
C14	34.5(15)	30.1(18)	29.7(14)	-1.8(12)	2.3(12)	-8.5(13)

Table S11 Bond Lengths for CCDC 2067817.

Atom	Atom	Length/Å	Atom	Atom	Length/Å
S1	O1	1.491(2)	C5	C10	1.392(4)
S1	N1	1.664(3)	C6	C7	1.381(4)
S1	C11	1.849(3)	C7	C8	1.376(4)
F1	C6	1.347(3)	C8	C9	1.373(4)
F2	C7	1.346(3)	C9	C10	1.384(4)
F3	C9	1.345(3)	C2	C3	1.463(4)
F4	C10	1.343(3)	C3	C4	1.186(5)
N1	C1	1.473(3)	C11	C12	1.526(4)
C1	C5	1.514(3)	C11	C13	1.525(4)
C1	C2	1.545(4)	C11	C14	1.524(4)
C5	C6	1.390(4)			

Table S12. Bond Angles for CCDC 2067817.

Atom	Atom	Atom	Angle/˚	Atom	Atom	Atom	Angle/˚
O1	S1	N1	111.80(12)	C9	C8	C7	117.7(2)
O1	S1	C11	106.71(12)	F3	C9	C8	119.9(2)
N1	S1	C11	95.86(13)	F3	C9	C10	118.5(3)
C1	N1	S1	117.3(2)	C8	C9	C10	121.6(3)
N1	C1	C5	110.8(2)	F4	C10	C5	120.5(2)
N1	C1	C2	107.4(2)	F4	C10	C9	118.0(3)
C5	C1	C2	111.4(2)	C9	C10	C5	121.5(3)
C6	C5	C1	121.1(3)	C3	C2	C1	111.2(3)
C6	C5	C10	116.1(2)	C4	C3	C2	176.6(4)
C10	C5	C1	122.8(3)	C12	C11	S1	110.23(19)
F1	C6	C5	119.8(2)	C13	C11	S1	105.0(2)
F1	C6	C7	118.1(2)	C13	C11	C12	110.9(2)
C7	C6	C5	122.1(3)	C14	C11	S1	107.33(18)
F2	C7	C6	118.8(3)	C14	C11	C12	112.2(3)
F2	C7	C8	120.2(2)	C14	C11	C13	110.9(2)
C8	C7	C6	121.1(3)				

Table S13. Torsion Angles for CCDC 2067817.

A	B	C	D	Angle/˚	A	B	C	D	Angle/˚
S1	N1	C1	C5	-75.3(3)	C1	C5	C6	C7	179.3(3)
S1	N1	C1	C2	162.84(18)	C1	C5	C10	F4	1.0(4)
F1	C6	C7	F2	-0.5(4)	C1	C5	C10	C9	-179.5(3)
F1	C6	C7	C8	178.8(3)	C5	C1	C2	C3	169.2(2)
F2	C7	C8	C9	178.8(3)	C5	C6	C7	F2	-179.2(2)
F3	C9	C10	F4	0.1(4)	C5	C6	C7	C8	0.1(4)
F3	C9	C10	C5	-179.5(3)	C6	C5	C10	F4	179.6(2)
O1	S1	N1	C1	-78.4(2)	C6	C5	C10	C9	-0.9(4)
O1	S1	C11	C12	-56.4(2)	C6	C7	C8	C9	-0.5(4)
O1	S1	C11	C13	63.1(2)	C7	C8	C9	F3	-179.8(3)
O1	S1	C11	C14	-178.9(2)	C7	C8	C9	C10	0.3(4)
N1	S1	C11	C12	58.5(2)	C8	C9	C10	F4	-180.0(3)
N1	S1	C11	C13	177.96(19)	C8	C9	C10	C5	0.5(4)
N1	S1	C11	C14	-64.0(2)	C10	C5	C6	F1	-178.1(2)
N1	C1	C5	C6	126.9(3)	C10	C5	C6	C7	0.6(4)
N1	C1	C5	C10	-54.6(4)	C2	C1	C5	C6	-113.7(3)
N1	C1	C2	C3	-69.3(3)	C2	C1	C5	C10	64.8(3)
C1	C5	C6	F1	0.5(4)	C11	S1	N1	C1	171.0(2)

Table S14 Hydrogen Atom Coordinates (Å×104) and Isotropic Displacement Parameters (Å2×103) for CCDC 2067817.

Atom	x	y	z	U(eq)
H1A	4220.77	1212.23	7595.31	29
H8	1918.92	-177.81	3852.08	36
H2A	4033.17	-3955.75	7635.3	35
H2B	5470.56	-2615.51	7364.99	35
H1	2510(40)	-1210(70)	8348(16)	38
H4	6358.76	-1741.01	10072.09	48
H12A	861.91	-1997.97	9091.41	44
H12B	-561.45	-951.47	9470.08	44
H12C	991.46	573.53	9568.43	44
H13A	-870.71	3900.06	8784.8	49
H13B	-2284.86	2061.8	8612.87	49
H13C	-1804.17	3418.83	7782.83	49
H14A	-1445.88	-322.49	6995.27	48
H14B	-1970.65	-1886.65	7764.1	48
H14C	-348.34	-2396.79	7493.96	48

VII. Computational details.

All DFT geometry optimizations were performed with the dispersion-corrected B97D functional[5] and 6-311+G(2d,2p) basis set as implemented within the Gaussian 16 series of programs.[6] Solvent effects were included with the conductor-like polarizable continuum model (CPCM)[7] to mimic the solvent (CH_2Cl_2 or THF) during both geometry optimizations and vibrational analysis. All energies presented for the reactant complex (RC), transition state (TS), and product (P) are given in Hartree. All energies have been corrected with zero-point energies (ZPE). Vibrational frequency calculations were performed at the same level of theory used for optimization. All transition states were verified to have only one negative eigenvalue in the Hessian matrix, describing the motion along the reaction coordinate. In addition, intrinsic reaction coordinate (IRC)[8] calculations were performed at the wB97D/6-311+G(2d,2p) level to verify the expected connections of the first-order saddle points with the local minima Found on the potential energy surface. Natural bond orbital (NBO)[9] analysis of charges was performed at TPSS-D3/def2-TZVPP level of theory.[10,11] Optimized structures were illustrated using CYLview20.3.[12]

VIII. Natural bond orbital (NBO) analysis of charges of the different atoms in sulfinyl imines.

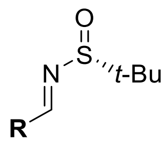

Table S15. NBO charges on different atoms of experimentally studied sulfinyl imines based on TPSS-D3/def2-TZVPP calculations.

Entry		R	S	O	N	C
1	**1a**	C_6F_5	1.1809	-0.8474	-0.5275	0.0766
2	**1b**	2,3,5,6-C_6HF_4	1.1808	-0.8478	-0.5243	0.0772
3	**1c**	2,4,6-$C_6H_2F_3$	1.1805	-0.8530	-0.5367	0.0816
4	**1d**	2,6-$C_6H_4F_2$	1.1802	-0.8538	-0.5339	0.0824
5	**1e**	2-C_6H_4F	1.1868	-0.8561	-0.5688	0.1005
6	**1f**	C_6H_5	1.1863	-0.8569	-0.5658	0.1016

Sulfinyl imine 1a

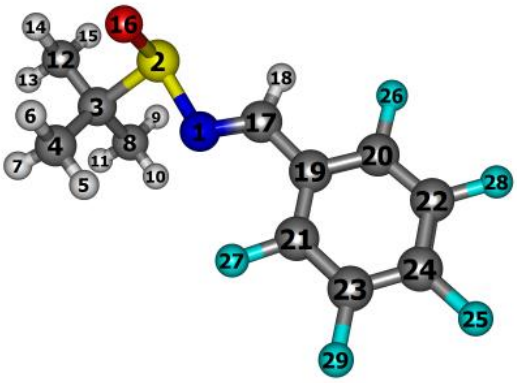

Summary of Natural Population Analysis:

Atom	No	Natural Charge	Natural Population Core	Valence	Rydberg	Total
N	1	-0.52755	1.99940	5.50854	0.01961	7.52755
S	2	1.18094	9.99904	4.70637	0.11364	14.81906
C	3	-0.15636	1.99943	4.13596	0.02098	6.15636
C	4	-0.60940	1.99935	4.60103	0.00901	6.60940
H	5	0.22397	0.00000	0.77454	0.00149	0.77603
H	6	0.22779	0.00000	0.77047	0.00173	0.77221
H	7	0.20369	0.00000	0.79457	0.00174	0.79631
C	8	-0.60653	1.99936	4.59962	0.00755	6.60653
H	9	0.20244	0.00000	0.79555	0.00202	0.79756
H	10	0.21865	0.00000	0.77967	0.00167	0.78135
H	11	0.21268	0.00000	0.78554	0.00179	0.78732
C	12	-0.60605	1.99937	4.59853	0.00815	6.60605
H	13	0.20997	0.00000	0.78826	0.00176	0.79003
H	14	0.22317	0.00000	0.77532	0.00151	0.77683
H	15	0.20350	0.00000	0.79484	0.00167	0.79650
O	16	-0.84736	1.99990	6.83647	0.01099	8.84736
C	17	0.07656	1.99932	3.89635	0.02777	5.92344
H	18	0.18011	0.00000	0.81322	0.00667	0.81989
C	19	-0.21310	1.99893	4.19613	0.01804	6.21310
C	20	0.34252	1.99846	3.63559	0.02343	5.65748
C	21	0.35462	1.99845	3.62306	0.02387	5.64538
C	22	0.23930	1.99833	3.73735	0.02503	5.76070
C	23	0.24104	1.99837	3.73551	0.02508	5.75896
C	24	0.27950	1.99839	3.69661	0.02550	5.72050
F	25	-0.24398	1.99994	7.23662	0.00742	9.24398
F	26	-0.26552	1.99994	7.25872	0.00686	9.26552
F	27	-0.24256	1.99994	7.23531	0.00731	9.24256
F	28	-0.25102	1.99994	7.24404	0.00704	9.25102
F	29	-0.25102	1.99994	7.24399	0.00708	9.25102
* Total *		-0.00000	45.98582	105.59778	0.41640	152.00000

Sulfinyl imine 1b

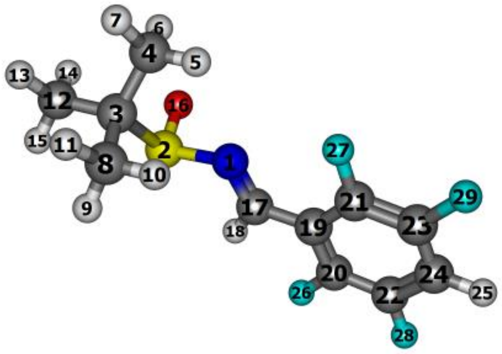

Summary of Natural Population Analysis:

Atom	No	Natural Charge	Natural Population Core	Valence	Rydberg	Total
N	1	-0.52432	1.99940	5.50552	0.01940	7.52432
S	2	1.18080	9.99904	4.70662	0.11354	14.81920
C	3	-0.15635	1.99943	4.13594	0.02099	6.15635
C	4	-0.60951	1.99935	4.60115	0.00901	6.60951
H	5	0.22460	0.00000	0.77391	0.00149	0.77540
H	6	0.22765	0.00000	0.77062	0.00173	0.77235
H	7	0.20328	0.00000	0.79498	0.00175	0.79672
C	8	-0.60652	1.99936	4.59964	0.00752	6.60652
H	9	0.20231	0.00000	0.79568	0.00202	0.79769
H	10	0.21907	0.00000	0.77928	0.00165	0.78093
H	11	0.21231	0.00000	0.78589	0.00180	0.78769
C	12	-0.60601	1.99937	4.59849	0.00815	6.60601
H	13	0.20965	0.00000	0.78857	0.00177	0.79035
H	14	0.22303	0.00000	0.77545	0.00152	0.77697
H	15	0.20334	0.00000	0.79499	0.00168	0.79666
O	16	-0.84778	1.99990	6.83690	0.01098	8.84778
C	17	0.07725	1.99932	3.89577	0.02766	5.92275
H	18	0.17984	0.00000	0.81349	0.00667	0.82016
C	19	-0.20065	1.99893	4.18410	0.01761	6.20065
C	20	0.33312	1.99842	3.64586	0.02260	5.66688
C	21	0.34220	1.99842	3.63418	0.02521	5.65780
C	22	0.30021	1.99840	3.67778	0.02360	5.69979
C	23	0.30231	1.99844	3.67545	0.02380	5.69769
C	24	-0.28618	1.99900	4.27453	0.01265	6.28618
H	25	0.25047	0.00000	0.74807	0.00146	0.74953
F	26	-0.27038	1.99994	7.26376	0.00668	9.27038
F	27	-0.24710	1.99994	7.24010	0.00706	9.24710
F	28	-0.26823	1.99994	7.26161	0.00667	9.26823
F	29	-0.26841	1.99994	7.26172	0.00674	9.26841
* Total *		-0.00000	43.98657	99.62005	0.39338	144.00000

Sulfinyl imine 1c

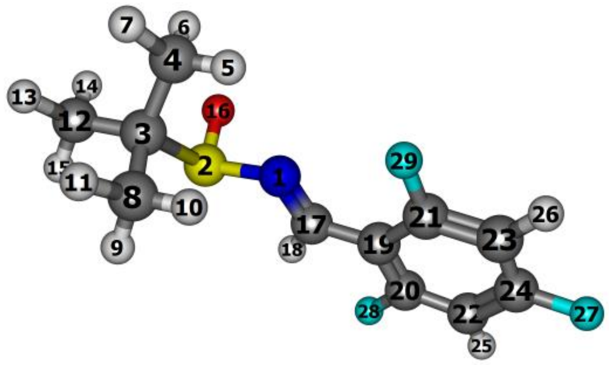

Summary of Natural Population Analysis:

Atom	No	Natural Charge	Natural Population Core	Valence	Rydberg	Total
N	1	-0.53669	1.99940	5.51790	0.01939	7.53669
S	2	1.18053	9.99904	4.70635	0.11408	14.81947
C	3	-0.15723	1.99943	4.13670	0.02110	6.15723
C	4	-0.60942	1.99936	4.60103	0.00903	6.60942
H	5	0.22430	0.00000	0.77419	0.00151	0.77570
H	6	0.22743	0.00000	0.77081	0.00176	0.77257
H	7	0.20186	0.00000	0.79637	0.00177	0.79814
C	8	-0.60613	1.99936	4.59922	0.00756	6.60613
H	9	0.20181	0.00000	0.79614	0.00205	0.79819
H	10	0.21898	0.00000	0.77934	0.00168	0.78102
H	11	0.21079	0.00000	0.78739	0.00182	0.78921
C	12	-0.60561	1.99937	4.59808	0.00816	6.60561
H	13	0.20837	0.00000	0.78984	0.00179	0.79163
H	14	0.22290	0.00000	0.77557	0.00153	0.77710
H	15	0.20280	0.00000	0.79550	0.00169	0.79720
O	16	-0.85303	1.99990	6.84219	0.01093	8.85303
C	17	0.08156	1.99932	3.89156	0.02757	5.91844
H	18	0.17716	0.00000	0.81595	0.00689	0.82284
C	19	-0.24232	1.99887	4.22625	0.01719	6.24232
C	20	0.42325	1.99855	3.55602	0.02217	5.57675
C	21	0.43353	1.99854	3.54504	0.02288	5.56647
C	22	-0.34303	1.99893	4.33144	0.01265	6.34303
C	23	-0.33947	1.99896	4.32803	0.01249	6.33947
C	24	0.40405	1.99854	3.57468	0.02273	5.59595
H	25	0.24854	0.00000	0.75005	0.00141	0.75146
H	26	0.24797	0.00000	0.75065	0.00137	0.75203
F	27	-0.27832	1.99994	7.27183	0.00655	9.27832
F	28	-0.28461	1.99994	7.27814	0.00653	9.28461
F	29	-0.25999	1.99994	7.25317	0.00688	9.25999
* Total *		-0.00000	41.98740	93.63945	0.37314	136.00000

Sulfinyl imine 1d

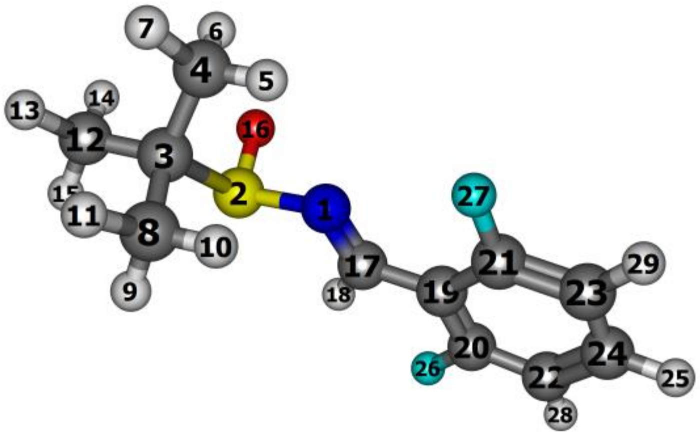

Summary of Natural Population Analysis:

Atom	No	Natural Charge	Natural Population Core	Valence	Rydberg	Total
N	1	-0.53394	1.99941	5.51543	0.01910	7.53394
S	2	1.18025	9.99904	4.70671	0.11399	14.81975
C	3	-0.15723	1.99943	4.13668	0.02113	6.15723
C	4	-0.60954	1.99936	4.60116	0.00903	6.60954
H	5	0.22500	0.00000	0.77349	0.00151	0.77500
H	6	0.22724	0.00000	0.77099	0.00176	0.77276
H	7	0.20131	0.00000	0.79691	0.00178	0.79869
C	8	-0.60611	1.99936	4.59922	0.00753	6.60611
H	9	0.20166	0.00000	0.79630	0.00204	0.79834
H	10	0.21948	0.00000	0.77886	0.00166	0.78052
H	11	0.21029	0.00000	0.78788	0.00183	0.78971
C	12	-0.60553	1.99937	4.59800	0.00816	6.60553
H	13	0.20792	0.00000	0.79027	0.00181	0.79208
H	14	0.22275	0.00000	0.77572	0.00154	0.77725
H	15	0.20259	0.00000	0.79570	0.00170	0.79741
O	16	-0.85383	1.99990	6.84301	0.01091	8.85383
C	17	0.08242	1.99932	3.89086	0.02740	5.91758
H	18	0.17701	0.00000	0.81614	0.00685	0.82299
C	19	-0.23216	1.99889	4.21566	0.01761	6.23216
C	20	0.41033	1.99853	3.56952	0.02162	5.58967
C	21	0.41863	1.99852	3.55915	0.02370	5.58137
C	22	-0.28274	1.99908	4.27033	0.01334	6.28274
C	23	-0.27862	1.99910	4.26610	0.01341	6.27862
C	24	-0.15005	1.99925	4.13767	0.01313	6.15005
H	25	0.21752	0.00000	0.78145	0.00103	0.78248
F	26	-0.29127	1.99994	7.28505	0.00627	9.29127
F	27	-0.26628	1.99994	7.25966	0.00667	9.26628
H	28	0.23176	0.00000	0.76697	0.00127	0.76824
H	29	0.23114	0.00000	0.76756	0.00130	0.76886
* Total *		0.00000	39.98844	87.65247	0.35909	128.00000

Sulfinyl imine 1e

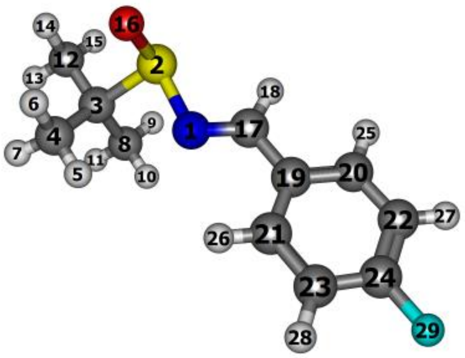

Summary of Natural Population Analysis:

Atom	No	Natural Charge	Natural Population Core	Valence	Rydberg	Total
N	1	-0.56879	1.99939	5.54981	0.01960	7.56879
S	2	1.18683	9.99904	4.69913	0.11499	14.81317
C	3	-0.15785	1.99942	4.13724	0.02118	6.15785
C	4	-0.60803	1.99936	4.59966	0.00901	6.60803
H	5	0.21824	0.00000	0.78013	0.00163	0.78176
H	6	0.22855	0.00000	0.76965	0.00180	0.77145
H	7	0.20194	0.00000	0.79627	0.00179	0.79806
C	8	-0.60540	1.99936	4.59846	0.00758	6.60540
H	9	0.20211	0.00000	0.79583	0.00206	0.79789
H	10	0.21684	0.00000	0.78148	0.00167	0.78316
H	11	0.21040	0.00000	0.78777	0.00183	0.78960
C	12	-0.60567	1.99937	4.59812	0.00817	6.60567
H	13	0.20786	0.00000	0.79033	0.00181	0.79214
H	14	0.22327	0.00000	0.77520	0.00153	0.77673
H	15	0.20285	0.00000	0.79544	0.00171	0.79715
O	16	-0.85610	1.99990	6.84528	0.01091	8.85610
C	17	0.10053	1.99930	3.87314	0.02702	5.89947
H	18	0.15530	0.00000	0.83757	0.00713	0.84470
C	19	-0.13575	1.99909	4.11843	0.01823	6.13575
C	20	-0.14344	1.99918	4.13158	0.01269	6.14344
C	21	-0.13538	1.99915	4.12301	0.01322	6.13538
C	22	-0.26750	1.99908	4.25523	0.01319	6.26750
C	23	-0.26039	1.99909	4.24849	0.01281	6.26039
C	24	0.37958	1.99853	3.60014	0.02175	5.62042
H	25	0.21105	0.00000	0.78754	0.00141	0.78895
H	26	0.23229	0.00000	0.76613	0.00158	0.76771
H	27	0.22854	0.00000	0.77008	0.00137	0.77146
H	28	0.22853	0.00000	0.77013	0.00134	0.77147
F	29	-0.29043	1.99994	7.28417	0.00632	9.29043
* Total *		-0.00000	37.98921	81.66546	0.34532	120.00000

Sulfinyl imine 1f

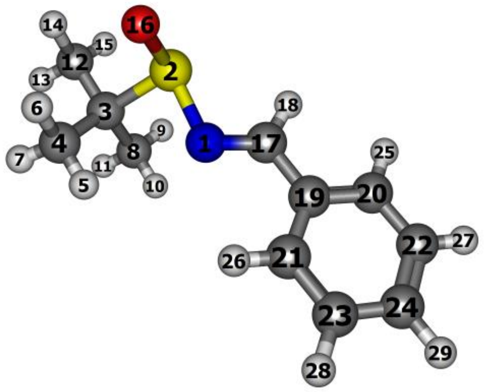

Summary of Natural Population Analysis:

Atom	No	Natural Charge	Natural Population Core	Valence	Rydberg	Total
N	1	-0.56578	1.99939	5.54678	0.01961	7.56578
S	2	1.18627	9.99904	4.69976	0.11493	14.81373
C	3	-0.15787	1.99942	4.13724	0.02120	6.15787
C	4	-0.60817	1.99936	4.59980	0.00901	6.60817
H	5	0.21888	0.00000	0.77948	0.00164	0.78112
H	6	0.22839	0.00000	0.76981	0.00180	0.77161
H	7	0.20138	0.00000	0.79682	0.00180	0.79862
C	8	-0.60547	1.99936	4.59853	0.00758	6.60547
H	9	0.20193	0.00000	0.79601	0.00206	0.79807
H	10	0.21732	0.00000	0.78100	0.00168	0.78268
H	11	0.20988	0.00000	0.78828	0.00184	0.79012
C	12	-0.60559	1.99937	4.59804	0.00818	6.60559
H	13	0.20742	0.00000	0.79076	0.00182	0.79258
H	14	0.22310	0.00000	0.77536	0.00154	0.77690
H	15	0.20265	0.00000	0.79564	0.00171	0.79735
O	16	-0.85690	1.99990	6.84611	0.01089	8.85690
C	17	0.10165	1.99930	3.87201	0.02703	5.89835
H	18	0.15432	0.00000	0.83850	0.00718	0.84568
C	19	-0.12263	1.99909	4.10481	0.01873	6.12263
C	20	-0.15928	1.99916	4.14746	0.01265	6.15928
C	21	-0.15193	1.99914	4.13971	0.01309	6.15193
C	22	-0.20418	1.99923	4.19164	0.01331	6.20418
C	23	-0.19908	1.99923	4.18677	0.01308	6.19908
C	24	-0.17726	1.99924	4.16492	0.01310	6.17726
H	25	0.20605	0.00000	0.79248	0.00146	0.79395
H	26	0.22724	0.00000	0.77116	0.00160	0.77276
H	27	0.20982	0.00000	0.78895	0.00124	0.79018
H	28	0.20979	0.00000	0.78899	0.00121	0.79021
H	29	0.20804	0.00000	0.79078	0.00118	0.79196
* Total *		-0.00000	35.99024	75.67760	0.33216	112.00000

IX. Cartesian coordinates of optimized structures.

Sulfinyl imine 1a

Center Number	Atomic Number	Atomic Type	Coordinates (Angstroms) X	Y	Z
1	7	0	-0.342465	-0.039253	0.216642
2	16	0	1.091084	-0.723996	-0.683629
3	6	0	2.345906	0.505719	0.064879
4	6	0	2.368183	0.307130	1.578933
5	1	0	1.422124	0.614701	2.031106
6	1	0	2.549447	-0.744536	1.823001
7	1	0	3.180282	0.908737	2.006624
8	6	0	1.914076	1.912545	-0.354711
9	1	0	1.805373	1.994725	-1.443441
10	1	0	0.968721	2.193435	0.118813
11	1	0	2.680549	2.632838	-0.042914
12	6	0	3.678828	0.099926	-0.579581
13	1	0	4.474504	0.750836	-0.197897
14	1	0	3.934242	-0.935335	-0.332647
15	1	0	3.650373	0.204705	-1.670741
16	8	0	1.370382	-2.076792	-0.045401
17	6	0	-1.412928	-0.144100	-0.485098
18	1	0	-1.417911	-0.536147	-1.510179
19	6	0	-2.739848	0.240098	0.019369
20	6	0	-3.860698	0.050325	-0.811943
21	6	0	-2.988470	0.798512	1.288986
22	6	0	-5.153378	0.381092	-0.419795
23	6	0	-4.276195	1.136736	1.700281
24	6	0	-5.360584	0.929166	0.846262
25	9	0	-6.596086	1.255385	1.241394
26	9	0	-3.697723	-0.477046	-2.042556
27	9	0	-1.991418	1.030196	2.150208
28	9	0	-6.193821	0.179686	-1.242935
29	9	0	-4.482311	1.667347	2.915486

Sulfinyl imine 1b

Center Number	Atomic Number	Atomic Type	Coordinates (Angstroms) X	Y	Z
1	7	0	-0.340409	-0.052449	0.221949
2	16	0	1.094104	-0.731373	-0.680845
3	6	0	2.344526	0.504962	0.065041
4	6	0	2.370003	0.306907	1.579111
5	1	0	1.422610	0.608704	2.032364
6	1	0	2.557832	-0.743647	1.823050
7	1	0	3.179076	0.913445	2.005681
8	6	0	1.905363	1.909606	-0.354118
9	1	0	1.792608	1.990492	-1.442566
10	1	0	0.960401	2.186866	0.122308
11	1	0	2.669819	2.633444	-0.045490
12	6	0	3.678275	0.105312	-0.581442
13	1	0	4.471814	0.759362	-0.200588
14	1	0	3.938414	-0.929013	-0.335423
15	1	0	3.647734	0.210472	-1.672520
16	8	0	1.380839	-2.082096	-0.041484
17	6	0	-1.409375	-0.147200	-0.483455
18	1	0	-1.412214	-0.530412	-1.511802
19	6	0	-2.737324	0.237810	0.020676
20	6	0	-3.855051	0.048683	-0.815544
21	6	0	-2.984974	0.795495	1.290735
22	6	0	-5.142972	0.385287	-0.410659
23	6	0	-4.279972	1.129573	1.685257
24	6	0	-5.369498	0.931157	0.846083
25	1	0	-6.371570	1.194430	1.166138
26	9	0	-3.686005	-0.478424	-2.047419
27	9	0	-1.986097	1.025533	2.152651
28	9	0	-6.176594	0.177965	-1.252141
29	9	0	-4.471986	1.661075	2.910214

Sulfinyl imine 1c

Center Number	Atomic Number	Atomic Type	Coordinates (Angstroms) X	Y	Z
1	7	0	-0.335417	-0.056567	0.238513
2	16	0	1.090869	-0.741536	-0.660484
3	6	0	2.344662	0.505690	0.061514
4	6	0	2.377625	0.328195	1.578062
5	1	0	1.431417	0.634705	2.030696
6	1	0	2.567026	-0.719102	1.834659
7	1	0	3.188024	0.940971	1.993324
8	6	0	1.902911	1.904778	-0.373087
9	1	0	1.786017	1.971803	-1.462068
10	1	0	0.958734	2.185719	0.102697
11	1	0	2.667424	2.633583	-0.076080
12	6	0	3.676126	0.099331	-0.585057
13	1	0	4.471300	0.758045	-0.215536
14	1	0	3.937106	-0.932083	-0.327888
15	1	0	3.640761	0.191092	-1.677236
16	8	0	1.390524	-2.084432	-0.008261
17	6	0	-1.407966	-0.155641	-0.464899
18	1	0	-1.407459	-0.549153	-1.489640
19	6	0	-2.732998	0.233722	0.027712
20	6	0	-3.861209	0.057755	-0.799409
21	6	0	-3.011430	0.790401	1.294625
22	6	0	-5.159282	0.384078	-0.439676
23	6	0	-4.288244	1.138296	1.714894
24	6	0	-5.338011	0.923682	0.829081
25	1	0	-5.989833	0.223493	-1.116392
26	1	0	-4.453043	1.562087	2.698443
27	9	0	-6.589025	1.255645	1.218749
28	9	0	-3.672184	-0.469102	-2.034440
29	9	0	-2.003527	1.009489	2.155869

Sulfinyl imine 1d

Center Number	Atomic Number	Atomic Type	Coordinates (Angstroms) X	Y	Z
1	7	0	-0.332951	-0.069477	0.242257
2	16	0	1.094187	-0.747044	-0.660177
3	6	0	2.343379	0.505381	0.061810
4	6	0	2.378251	0.326514	1.578141
5	1	0	1.430659	0.627606	2.031486
6	1	0	2.572956	-0.720184	1.833294
7	1	0	3.185902	0.942839	1.993648
8	6	0	1.894987	1.902937	-0.370830
9	1	0	1.775704	1.970211	-1.459571
10	1	0	0.950468	2.179399	0.106871
11	1	0	2.656935	2.634854	-0.074740
12	6	0	3.676109	0.105700	-0.586196
13	1	0	4.468967	0.766962	-0.216134
14	1	0	3.941324	-0.925127	-0.330910
15	1	0	3.639603	0.199034	-1.678220
16	8	0	1.401596	-2.089152	-0.009603
17	6	0	-1.404290	-0.158439	-0.464429
18	1	0	-1.402019	-0.542849	-1.492483
19	6	0	-2.730307	0.231431	0.028398
20	6	0	-3.856171	0.055641	-0.801646
21	6	0	-3.008691	0.788003	1.294573
22	6	0	-5.151740	0.384712	-0.435413
23	6	0	-4.289775	1.133287	1.704927
24	6	0	-5.359787	0.927848	0.833125
25	1	0	-6.365302	1.193352	1.145550
26	9	0	-3.660456	-0.472674	-2.038909
27	9	0	-1.997057	1.006748	2.156461
28	1	0	-5.966015	0.215000	-1.131261
29	1	0	-4.430854	1.555804	2.693991

Sulfinyl imine 1e

Center Number	Atomic Number	Atomic Type	Coordinates (Angstroms) X	Y	Z
1	7	0	-0.364759	-0.130948	0.260239
2	16	0	1.086566	-0.823530	-0.567465
3	6	0	2.303938	0.507253	0.067891
4	6	0	2.335404	0.438640	1.593080
5	1	0	1.382815	0.760513	2.021360
6	1	0	2.540388	-0.584825	1.923470
7	1	0	3.133426	1.092819	1.967033
8	6	0	1.831012	1.860122	-0.467321
9	1	0	1.719108	1.845479	-1.558766
10	1	0	0.876482	2.151359	-0.018969
11	1	0	2.574922	2.627972	-0.220498
12	6	0	3.648597	0.089086	-0.542762
13	1	0	4.425854	0.791958	-0.219149
14	1	0	3.932884	-0.914596	-0.211324
15	1	0	3.616322	0.101034	-1.638801
16	8	0	1.424641	-2.106184	0.180088
17	6	0	-1.417594	-0.211875	-0.475184
18	1	0	-1.387202	-0.596464	-1.507055
19	6	0	-2.741808	0.199482	0.002132
20	6	0	-3.836070	0.136732	-0.877470
21	6	0	-2.943646	0.655334	1.318804
22	6	0	-5.109732	0.522786	-0.465194
23	6	0	-4.207501	1.041744	1.746309
24	6	0	-5.267131	0.969040	0.842232
25	1	0	-3.686933	-0.219222	-1.894502
26	1	0	-2.095338	0.692768	1.995489
27	1	0	-5.965603	0.480781	-1.130940
28	1	0	-4.386514	1.392236	2.757842
29	9	0	-6.503151	1.347440	1.258240

Sulfinyl imine 1f

Center Number	Atomic Number	Atomic Type	Coordinates (Angstroms) X	Y	Z
1	7	0	-0.361295	-0.143824	0.268626
2	16	0	1.090122	-0.832038	-0.562295
3	6	0	2.303043	0.506255	0.066830
4	6	0	2.339212	0.440426	1.592060
5	1	0	1.386001	0.757608	2.022399
6	1	0	2.550576	-0.581443	1.923420
7	1	0	3.134791	1.099522	1.962728
8	6	0	1.821888	1.855921	-0.469062
9	1	0	1.705913	1.838677	-1.560064
10	1	0	0.867642	2.143455	-0.017780
11	1	0	2.563149	2.627737	-0.226473
12	6	0	3.648017	0.093814	-0.546894
13	1	0	4.423032	0.800439	-0.225934
14	1	0	3.937605	-0.908328	-0.215311
15	1	0	3.612774	0.104548	-1.642870
16	8	0	1.437211	-2.111178	0.187450
17	6	0	-1.413277	-0.217076	-0.468448
18	1	0	-1.382079	-0.596646	-1.502258
19	6	0	-2.738996	0.197632	0.007830
20	6	0	-3.827056	0.142711	-0.879361
21	6	0	-2.944897	0.646644	1.325215
22	6	0	-5.099163	0.533289	-0.462605
23	6	0	-4.215300	1.032830	1.738217
24	6	0	-5.294196	0.978995	0.846338
25	1	0	-3.669473	-0.208842	-1.897191
26	1	0	-2.098938	0.677265	2.005536
27	1	0	-5.935832	0.488219	-1.154525
28	1	0	-4.372241	1.375620	2.757585
29	1	0	-6.285100	1.281930	1.174751

RC for *Si* attack in DCM of 1a with 2a

Center Number	Atomic Number	Atomic Type	Coordinates (Angstroms) X	Y	Z
1	12	0	1.752059	0.993972	1.338645
2	6	0	0.168084	0.976086	2.770136
3	6	0	-0.508465	-0.099134	2.991829
4	6	0	-1.144228	-1.244342	3.141462
5	6	0	-0.464268	-1.365487	0.052405
6	7	0	0.694645	-0.824521	0.013671
7	1	0	-0.593773	-2.414239	0.308682
8	16	0	2.004027	-1.808736	0.482680
9	8	0	2.804406	-0.775112	1.253745
10	6	0	-1.687624	-0.640647	-0.252089
11	6	0	-2.908015	-1.284560	-0.032947
12	6	0	-1.737088	0.662058	-0.753997
13	6	0	-4.118286	-0.669696	-0.274392
14	6	0	-2.939964	1.292052	-1.006002
15	6	0	-4.130850	0.626986	-0.762093
16	35	0	2.984442	2.860563	0.251230
17	6	0	2.845665	-2.002998	-1.153502
18	6	0	3.046930	-0.651062	-1.818994
19	1	0	3.605494	0.029918	-1.177971
20	1	0	3.621684	-0.802819	-2.733265
21	1	0	2.099500	-0.185392	-2.083553
22	6	0	1.960228	-2.934008	-1.976311
23	1	0	1.758235	-3.871048	-1.455633
24	1	0	1.014943	-2.462675	-2.243370
25	1	0	2.483257	-3.174884	-2.901855
26	6	0	4.179822	-2.663355	-0.804593
27	1	0	4.039726	-3.618937	-0.297716
28	1	0	4.722853	-2.850674	-1.730925
29	1	0	4.789025	-2.014336	-0.177321
30	1	0	-0.769050	-2.007781	3.811315
31	1	0	-2.095398	-1.429544	2.660040
32	1	0	-0.041888	1.826008	3.419160
33	9	0	-5.256914	-1.302805	-0.043301
34	9	0	-5.278246	1.228102	-0.999210
35	9	0	-2.924366	-2.525256	0.438106
36	9	0	-0.632691	1.333327	-1.031033
37	9	0	-2.962142	2.525526	-1.483921

TS for *Si* attack in DCM of 1a with 2a

Imaginary frequency: -362.8069

Center Number	Atomic Number	Atomic Type	Coordinates (Angstroms) X	Y	Z
1	12	0	1.935766	0.978448	1.031645
2	6	0	0.807175	0.950552	2.953043
3	6	0	-0.075306	0.058769	3.020668
4	6	0	-0.928905	-0.969125	2.822759
5	6	0	-0.473307	-1.183641	0.600035
6	7	0	0.720036	-0.722492	0.271613
7	1	0	-0.609878	-2.242822	0.776230
8	16	0	2.012215	-1.770958	0.523472
9	8	0	3.064002	-0.758491	0.971005
10	6	0	-1.685083	-0.490808	0.129041
11	6	0	-2.879861	-1.206445	0.080273
12	6	0	-1.733346	0.832060	-0.297301
13	6	0	-4.058501	-0.653717	-0.377023
14	6	0	-2.903404	1.408759	-0.757673
15	6	0	-4.069076	0.664835	-0.800020
16	35	0	2.752482	2.860665	-0.352488
17	6	0	2.454561	-2.199635	-1.222586
18	6	0	2.655936	-0.936672	-2.043990
19	1	0	3.437770	-0.307977	-1.619939
20	1	0	2.961162	-1.224264	-3.050788
21	1	0	1.738444	-0.355316	-2.117334

22	6	0	1.307121	-3.054682	-1.751670
23	1	0	1.106449	-3.912688	-1.107819
24	1	0	0.392751	-2.473064	-1.864963
25	1	0	1.583323	-3.436228	-2.734880
26	6	0	3.746546	-3.007096	-1.104569
27	1	0	3.609381	-3.903506	-0.498300
28	1	0	4.055016	-3.319992	-2.102454
29	1	0	4.546817	-2.407281	-0.673132
30	1	0	-0.651830	-1.949528	3.191381
31	1	0	-1.988888	-0.769627	2.744820
32	1	0	1.160571	1.608153	3.734179
33	9	0	-2.903470	-2.470564	0.496957
34	9	0	-5.172026	-1.371119	-0.411575
35	9	0	-5.189312	1.211526	-1.237748
36	9	0	-2.912746	2.672567	-1.154536
37	9	0	-0.653180	1.606925	-0.271049

PRODUCT for *Si* attack in DCM of 1a with 2a

Center Number	Atomic Number	Atomic Type	Coordinates (Angstroms) X	Y	Z
1	12	0	2.221079	0.606913	1.256093
2	6	0	0.679391	1.482991	3.341757
3	6	0	0.081226	0.488502	3.032821
4	6	0	-0.620642	-0.746915	2.693747
5	6	0	-0.463193	-1.180705	1.206702
6	7	0	0.820327	-0.796832	0.661370
7	1	0	-0.568945	-2.264982	1.216033
8	16	0	1.939639	-1.957246	0.468345
9	8	0	3.204152	-1.156817	0.867364
10	6	0	-1.582275	-0.669652	0.313139
11	6	0	-2.516304	-1.545714	-0.217455
12	6	0	-1.694017	0.658234	-0.071792
13	6	0	-3.510355	-1.137811	-1.090690
14	6	0	-2.675107	1.096940	-0.941737
15	6	0	-3.589857	0.193077	-1.454204
16	35	0	3.115901	2.876001	0.948192
17	6	0	2.119627	-2.142623	-1.366917
18	6	0	2.381984	-0.790856	-2.008459
19	1	0	3.324784	-0.365676	-1.665598
20	1	0	2.440363	-0.914244	-3.090821
21	1	0	1.574287	-0.091359	-1.791136
22	6	0	0.796000	-2.737878	-1.839057
23	1	0	0.549146	-3.656446	-1.303070
24	1	0	-0.020390	-2.026956	-1.710730
25	1	0	0.869124	-2.980861	-2.899717
26	6	0	3.279421	-3.110342	-1.583244
27	1	0	3.097287	-4.069029	-1.094462
28	1	0	3.400674	-3.297050	-2.651229
29	1	0	4.211072	-2.694699	-1.200844
30	1	0	-0.203028	-1.540002	3.315576
31	1	0	-1.673406	-0.652707	2.961404
32	1	0	1.161332	2.385254	3.637837
33	9	0	-2.467073	-2.844561	0.091762
34	9	0	-4.379012	-2.014081	-1.581330
35	9	0	-4.535205	0.602640	-2.287553
36	9	0	-2.746767	2.376849	-1.286663
37	9	0	-0.836669	1.567775	0.392115

RC for *Re* attack in DCM of 1a with 2a

Center Number	Atomic Number	Atomic Type	Coordinates (Angstroms) X	Y	Z
1	12	0	-2.095113	1.332956	0.402503
2	6	0	-1.080424	1.065005	2.257687
3	6	0	-0.245928	0.104134	2.480627
4	6	0	0.578434	-0.909065	2.643881
5	6	0	0.169037	-1.194897	-1.135451
6	7	0	-0.894131	-0.609008	-0.756357
7	1	0	0.159116	-2.053101	-1.803639
8	16	0	-2.419814	-1.091634	-1.346055
9	8	0	-3.236096	0.053696	-0.780086
10	6	0	1.480768	-0.774374	-0.650833
11	6	0	2.463196	-1.743153	-0.471756
12	6	0	1.798657	0.539053	-0.314621
13	6	0	3.702737	-1.437583	0.053102
14	6	0	3.037719	0.868719	0.197345
15	6	0	3.987001	-0.123814	0.388943
16	35	0	-2.665424	3.631879	-0.397325
17	6	0	-2.813948	-2.570840	-0.281662
18	6	0	-2.742421	-2.206267	1.190624
19	1	0	-3.439059	-1.405902	1.434677
20	1	0	-3.024992	-3.085235	1.771181
21	1	0	-1.738244	-1.906800	1.487430
22	6	0	-1.838779	-3.682944	-0.649222
23	1	0	-1.781415	-3.845778	-1.726813
24	1	0	-0.840450	-3.498179	-0.255390
25	1	0	-2.196743	-4.607935	-0.196962
26	6	0	-4.240654	-2.922204	-0.709045
27	1	0	-4.295100	-3.184950	-1.765839
28	1	0	-4.565649	-3.786104	-0.129228
29	1	0	-4.928190	-2.101539	-0.509431
30	1	0	1.628591	-0.829046	2.390286
31	1	0	0.239655	-1.842714	3.076280
32	1	0	-1.201858	1.780006	3.072896
33	9	0	2.195931	-3.009626	-0.772111
34	9	0	4.610869	-2.382064	0.239217
35	9	0	5.168277	0.185970	0.884702
36	9	0	3.327666	2.123445	0.498998
37	9	0	0.934220	1.519829	-0.519746

TS for *Re* attack in DCM of 1a with 2a

Imaginary frequency: -346.1914

Center Number	Atomic Number	Atomic Type	Coordinates (Angstroms) X	Y	Z
1	12	0	-1.707010	1.186986	0.260833
2	6	0	-0.968204	0.609129	2.320738
3	6	0	-0.307615	-0.456164	2.372707
4	6	0	0.326346	-1.624472	2.118277
5	6	0	0.293554	-1.305374	-0.163957
6	7	0	-0.752974	-0.600948	-0.522863
7	1	0	0.317240	-2.377761	-0.295192
8	16	0	-2.169664	-1.163964	-1.220471
9	8	0	-3.020753	0.067260	-0.912543
10	6	0	1.628222	-0.673310	-0.170857
11	6	0	2.748941	-1.500520	-0.141775
12	6	0	1.871347	0.694240	-0.229498
13	6	0	4.038395	-1.010777	-0.175112
14	6	0	3.153912	1.210846	-0.262172
15	6	0	4.242252	0.357336	-0.235283
16	35	0	-2.167654	3.595540	-0.086798
17	6	0	-2.943843	-2.527869	-0.213458
18	6	0	-3.272059	-2.066219	1.193417
19	1	0	-3.913787	-1.186491	1.176973
20	1	0	-3.808578	-2.866345	1.705224
21	1	0	-2.378999	-1.832109	1.766209

22	6	0	-2.009634	-3.734026	-0.242709
23	1	0	-1.617052	-3.925426	-1.243218
24	1	0	-1.180719	-3.625780	0.453139
25	1	0	-2.573406	-4.616964	0.059776
26	6	0	-4.220482	-2.820752	-1.009022
27	1	0	-3.998450	-3.142405	-2.027139
28	1	0	-4.757291	-3.628443	-0.510618
29	1	0	-4.873212	-1.949732	-1.046931
30	1	0	1.400485	-1.681587	2.223390
31	1	0	-0.212414	-2.550117	2.287663
32	1	0	-1.316856	1.251729	3.115112
33	9	0	2.584479	-2.819759	-0.061636
34	9	0	5.074120	-1.836217	-0.145786
35	9	0	5.469716	0.845379	-0.262756
36	9	0	3.343719	2.520267	-0.316602
37	9	0	0.875368	1.584756	-0.258258

PRODUCT for *Re* attack in DCM of 1a with 2a

Center Number	Atomic Number	Atomic Type	Coordinates (Angstroms) X	Y	Z
1	12	0	-2.024246	0.209499	-0.219732
2	6	0	0.480012	2.361783	-1.081365
3	6	0	-0.944980	2.679260	-1.029076
4	6	0	-2.121675	2.907204	-0.955783
5	6	0	0.847290	1.442630	0.107109
6	7	0	0.050877	0.217641	0.171443
7	1	0	0.563471	1.993696	1.002319
8	16	0	0.297441	-0.895894	-0.999033
9	8	0	-1.163621	-1.130240	-1.478646
10	6	0	2.341260	1.193021	0.247696
11	6	0	2.865299	1.059980	1.528047
12	6	0	3.234225	1.007692	-0.797218
13	6	0	4.191844	0.760280	1.769794
14	6	0	4.570559	0.706827	-0.587392
15	6	0	5.052647	0.580950	0.701230
16	35	0	-4.313261	0.441874	0.669293
17	6	0	0.686473	-2.459737	-0.081321
18	6	0	-0.402944	-2.770371	0.930774
19	1	0	-1.365754	-2.920228	0.443234
20	1	0	-0.144229	-3.689658	1.457988
21	1	0	-0.492716	-1.972611	1.668142
22	6	0	2.030289	-2.232088	0.602518
23	1	0	2.797494	-1.907225	-0.103168
24	1	0	1.942909	-1.491218	1.395033
25	1	0	2.364553	-3.169683	1.048158
26	6	0	0.786815	-3.543381	-1.152570
27	1	0	1.555790	-3.306708	-1.889697
28	1	0	1.054557	-4.489450	-0.680263
29	1	0	-0.163575	-3.675595	-1.667949
30	1	0	-3.155975	3.155301	-0.905517
31	1	0	1.058650	3.285792	-1.038958
32	1	0	0.699627	1.883191	-2.033530
33	9	0	6.327408	0.292150	0.912550
34	9	0	4.640908	0.635191	3.012120
35	9	0	2.065105	1.193564	2.586473
36	9	0	5.387184	0.535182	-1.619454
37	9	0	2.837156	1.094738	-2.069103

RC for *Si* attack in THF of 1a with 2a

Center Number	Atomic Number	Atomic Type	X	Y	Z
1	16	0	-1.305457	1.969138	-1.216155
2	7	0	-0.164330	0.737919	-0.639880
3	8	0	-0.660448	2.682255	-2.344182
4	6	0	1.020257	0.951419	-1.054390
5	1	0	1.204419	1.807815	-1.703601
6	6	0	2.199201	0.180738	-0.664814
7	6	0	2.208941	-1.175937	-0.372722
8	6	0	3.401541	0.869354	-0.526474
9	6	0	3.343803	-1.822900	0.067265
10	6	0	4.551391	0.248544	-0.079122
11	6	0	4.518827	-1.103130	0.221743
12	12	0	-1.119260	-1.057832	0.301214
13	8	0	-2.984338	-0.270746	0.781290
14	6	0	-4.114626	-0.097815	-0.094298
15	6	0	-5.173172	-1.076663	0.417642
16	6	0	-4.712359	-1.422227	1.851340
17	6	0	-3.539858	-0.482477	2.092429
18	1	0	-5.201758	-1.967794	-0.204500
19	1	0	-2.751237	-0.878890	2.724806
20	1	0	-5.495502	-1.278172	2.591721
21	1	0	-4.378807	-2.456494	1.906602
22	1	0	-6.159793	-0.619661	0.402058
23	1	0	-4.444404	0.940262	-0.011644
24	1	0	-3.866464	0.483217	2.485735
25	1	0	-3.778457	-0.297768	-1.106285
26	6	0	-1.165209	3.144654	0.242758
27	6	0	-2.069854	4.303506	-0.181798
28	1	0	-2.099717	5.032023	0.628822
29	1	0	-1.692250	4.797186	-1.075408
30	1	0	-3.091460	3.968027	-0.367896
31	6	0	0.274938	3.604110	0.404540
32	1	0	0.286809	4.450615	1.092349
33	1	0	0.903884	2.824724	0.833516
34	1	0	0.695061	3.940181	-0.543103
35	6	0	-1.699031	2.479080	1.502455
36	1	0	-2.737386	2.176139	1.382200
37	1	0	-1.105459	1.615584	1.799054
38	1	0	-1.655769	3.208886	2.312605
39	35	0	-1.901979	-2.497354	-1.610212
40	6	0	1.589259	0.699781	2.697453
41	1	0	1.290858	1.616093	3.191568
42	1	0	2.629946	0.599063	2.413886
43	6	0	0.728338	-0.273495	2.486622
44	6	0	-0.136478	-1.187383	2.191079
45	1	0	-0.285214	-1.967542	2.937983
46	9	0	1.111556	-1.905935	-0.561627
47	9	0	3.448037	2.170793	-0.784476
48	9	0	5.673448	0.933831	0.070059
49	9	0	5.609183	-1.709201	0.647260
50	9	0	3.320888	-3.117686	0.333766

TS for *Si* attack in THF of 1a with 2a

Imaginary frequency: -355.8269

Center Number	Atomic Number	Atomic Type	X	Y	Z
1	16	0	-1.101403	1.938963	-1.312504
2	7	0	-0.099495	0.802764	-0.493841
3	8	0	-0.215086	2.729292	-2.211897
4	6	0	1.040362	1.258378	-0.009311
5	1	0	1.242377	2.320915	-0.060906
6	6	0	2.276497	0.450167	-0.013085
7	6	0	2.359642	-0.897582	-0.327698
8	6	0	3.485870	1.089670	0.257808

9	6	0	3.558147	-1.584397	-0.365931
10	6	0	4.698785	0.432915	0.228786
11	6	0	4.735640	-0.916146	-0.083665
12	12	0	-0.967432	-1.077293	0.027058
13	8	0	-2.963672	-0.507837	0.075989
14	6	0	-3.836899	-0.505429	-1.082288
15	6	0	-5.171907	-1.026695	-0.582146
16	6	0	-5.170294	-0.578481	0.878690
17	6	0	-3.724565	-0.804782	1.272739
18	1	0	-5.200030	-2.114406	-0.644285
19	1	0	-3.539500	-1.844818	1.547054
20	1	0	-5.426622	0.478840	0.957235
21	1	0	-5.854363	-1.147911	1.502800
22	1	0	-6.003614	-0.623722	-1.154602
23	1	0	-3.900130	0.521585	-1.441489
24	1	0	-3.362916	-0.149498	2.061456
25	1	0	-3.385349	-1.131960	-1.848468
26	6	0	-1.711336	3.189113	-0.054083
27	6	0	-2.771788	3.951966	-0.855814
28	1	0	-3.248252	4.683053	-0.201247
29	1	0	-2.326189	4.484039	-1.695431
30	1	0	-3.547784	3.285255	-1.235325
31	6	0	-0.600896	4.142009	0.366276
32	1	0	-1.050921	5.018101	0.836024
33	1	0	0.077709	3.696227	1.088477
34	1	0	-0.031516	4.475483	-0.500392
35	6	0	-2.355135	2.469760	1.121068
36	1	0	-3.213598	1.883996	0.797266
37	1	0	-1.660737	1.804627	1.630776
38	1	0	-2.707259	3.213321	1.838024
39	35	0	-1.429826	-3.455952	-0.637949
40	6	0	0.983167	1.333264	2.305229
41	1	0	0.485830	2.263053	2.551968
42	1	0	2.048526	1.308633	2.483974
43	6	0	0.268312	0.187181	2.386681
44	6	0	-0.437656	-0.830451	2.181745
45	1	0	-0.866230	-1.529807	2.883450
46	9	0	1.261901	-1.607055	-0.634441
47	9	0	3.485492	2.382128	0.573837
48	9	0	5.821774	1.080428	0.499426
49	9	0	5.888362	-1.559976	-0.111597
50	9	0	3.582267	-2.871833	-0.671281

PRODUCT for *Si* attack in THF of 1a with 2a

Center Number	Atomic Number	Atomic Type	Coordinates (Angstroms) X	Y	Z
1	16	0	-0.720413	1.743188	-1.304029
2	7	0	-0.182982	0.714929	-0.152471
3	8	0	0.435712	2.388295	-2.010258
4	6	0	0.944751	1.131231	0.660147
5	1	0	1.095636	2.203008	0.587982
6	6	0	2.266729	0.527749	0.200630
7	6	0	2.434316	-0.791936	-0.179206
8	6	0	3.393718	1.331445	0.106823
9	6	0	3.634127	-1.296229	-0.644614
10	6	0	4.611914	0.862975	-0.351862
11	6	0	4.733025	-0.461026	-0.732114
12	12	0	-1.045999	-1.109298	-0.024861
13	8	0	-3.031979	-0.662445	0.272386
14	6	0	-3.990620	-0.627158	-0.812809
15	6	0	-5.307944	-0.271488	-0.152388
16	6	0	-5.177736	-0.953237	1.209468
17	6	0	-3.712158	-0.739240	1.548202
18	1	0	-6.158210	-0.628306	-0.727869
19	1	0	-3.276220	-1.555879	2.119113
20	1	0	-5.832647	-0.526861	1.965028
21	1	0	-5.394366	-2.017578	1.122584
22	1	0	-5.398035	0.808685	-0.034650
23	1	0	-3.638395	0.108335	-1.533810
24	1	0	-3.539758	0.202061	2.070071
25	1	0	-4.017005	-1.614137	-1.276322

26	6	0	-1.543215	3.186345	-0.422564
27	6	0	-2.224366	3.952630	-1.557725
28	1	0	-2.805167	4.778947	-1.143771
29	1	0	-1.487926	4.362726	-2.247748
30	1	0	-2.905397	3.310426	-2.119490
31	6	0	-0.535240	4.085661	0.282185
32	1	0	-0.996204	5.053144	0.491739
33	1	0	-0.211809	3.670534	1.236395
34	1	0	0.336489	4.253247	-0.350471
35	6	0	-2.579971	2.628662	0.540813
36	1	0	-3.304568	2.000134	0.022691
37	1	0	-2.114886	2.030250	1.323514
38	1	0	-3.123457	3.450940	1.010497
39	35	0	-1.298132	-3.536934	-0.499548
40	6	0	0.658869	0.841306	2.151312
41	1	0	-0.058964	1.580722	2.508988
42	1	0	1.564567	0.948136	2.749330
43	6	0	0.090526	-0.487220	2.369186
44	6	0	-0.383371	-1.578597	2.532784
45	1	0	-0.758467	-2.557979	2.719439
46	9	0	1.411095	-1.663484	-0.107426
47	9	0	3.325557	2.615533	0.465396
48	9	0	5.660603	1.672721	-0.429145
49	9	0	5.892567	-0.927634	-1.171342
50	9	0	3.737824	-2.570329	-0.999326

RC for *Re* attack in THF of 1a with 2a

Center Number	Atomic Number	Atomic Type	Coordinates (Angstroms) X	Y	Z
1	16	0	-1.587562	2.185817	-0.689907
2	7	0	-0.300648	1.100072	-0.159539
3	8	0	-0.944492	3.314874	-1.406107
4	6	0	0.853655	1.513225	-0.512054
5	1	0	0.931858	2.440484	-1.080774
6	6	0	2.105187	0.828234	-0.210463
7	6	0	3.211118	1.061568	-1.025662
8	6	0	2.281250	-0.030531	0.869826
9	6	0	4.426387	0.445552	-0.799848
10	6	0	3.485829	-0.650927	1.121900
11	6	0	4.560417	-0.414907	0.277474
12	12	0	-0.789307	-1.122237	0.183258
13	35	0	-1.768029	-1.961444	2.328130
14	8	0	-2.421677	-0.956797	-1.058505
15	6	0	-3.722878	-1.514116	-0.734991
16	6	0	-4.512772	-1.444497	-2.027802
17	6	0	-3.421433	-1.627600	-3.081344
18	6	0	-2.277908	-0.824268	-2.495561
19	1	0	-4.987182	-0.468618	-2.134603
20	1	0	-2.355997	0.235231	-2.742152
21	1	0	-3.135773	-2.676674	-3.158922
22	1	0	-3.709362	-1.266329	-4.065550
23	1	0	-5.282961	-2.210627	-2.069970
24	1	0	-3.574263	-2.540338	-0.400590
25	1	0	-1.295619	-1.201259	-2.769245
26	1	0	-4.141314	-0.928941	0.079578
27	6	0	0.732667	-2.189890	-0.909195
28	1	0	1.475983	-2.609218	-0.227008
29	1	0	1.255608	-1.564906	-1.637452
30	6	0	-2.067474	2.839352	0.990244
31	6	0	-3.074627	3.940961	0.654354
32	1	0	-3.457918	4.351375	1.588789
33	1	0	-2.610109	4.746086	0.088121
34	1	0	-3.922022	3.552950	0.086850
35	6	0	-0.848418	3.404007	1.701040
36	1	0	-1.181484	3.967653	2.573200
37	1	0	-0.180523	2.616304	2.045209
38	1	0	-0.297833	4.084699	1.051024
39	6	0	-2.737763	1.711929	1.761985
40	1	0	-3.536536	1.245938	1.182943
41	1	0	-2.033427	0.941749	2.068502
42	1	0	-3.184926	2.126743	2.666280

43	6	0	0.010556	-3.242064	-1.576214
44	6	0	-0.677488	-4.081346	-2.105582
45	1	0	-1.244904	-4.841182	-2.584487
46	9	0	3.620971	-1.469744	2.151352
47	9	0	1.272204	-0.264466	1.706479
48	9	0	3.102100	1.877270	-2.064971
49	9	0	5.713626	-1.010271	0.503035
50	9	0	5.455361	0.668645	-1.600814

TS for *Re* attack in THF of 1a with 2a

Imaginary frequency: -378.6744

Center Number	Atomic Number	Atomic Type	Coordinates (Angstroms) X	Y	Z
1	16	0	-1.489237	-1.891829	-0.393813
2	7	0	-0.291007	-0.692182	-0.087996
3	8	0	-1.018991	-3.193253	0.147660
4	6	0	0.824778	-1.181079	0.420311
5	1	0	0.909703	-2.260377	0.496512
6	6	0	2.109302	-0.467686	0.261411
7	6	0	3.292258	-1.166456	0.496598
8	6	0	2.249024	0.849327	-0.155775
9	6	0	4.539365	-0.595842	0.335904
10	6	0	3.484012	1.449301	-0.323798
11	6	0	4.635963	0.723795	-0.075407
12	12	0	-1.053158	1.210494	0.498427
13	35	0	-1.812811	2.942014	-1.018646
14	8	0	-2.910539	0.527756	1.188318
15	6	0	-4.153388	0.663349	0.461322
16	6	0	-5.204908	0.060840	1.373216
17	6	0	-4.423601	-1.057739	2.061296
18	6	0	-3.061360	-0.418817	2.270230
19	1	0	-6.069067	-0.297048	0.818079
20	1	0	-2.238607	-1.129471	2.226341
21	1	0	-4.868169	-1.383121	2.999021
22	1	0	-4.336034	-1.921867	1.402338
23	1	0	-5.544646	0.797825	2.101732
24	1	0	-4.287257	1.716119	0.232793
25	1	0	-2.999158	0.133649	3.207768
26	1	0	-4.063292	0.104016	-0.472122
27	6	0	0.833052	-1.143095	2.651623
28	1	0	1.886320	-1.326382	2.810475
29	1	0	0.171480	-1.968077	2.889337
30	6	0	-1.279794	-1.988601	-2.242373
31	6	0	-2.308494	-3.035194	-2.669295
32	1	0	-2.279660	-3.145056	-3.754171
33	1	0	-2.095738	-4.003368	-2.217676
34	1	0	-3.320970	-2.735453	-2.391724
35	6	0	0.133524	-2.440708	-2.571793
36	1	0	0.200900	-2.660857	-3.638420
37	1	0	0.863355	-1.664206	-2.345340
38	1	0	0.389231	-3.345355	-2.019649
39	6	0	-1.608069	-0.620223	-2.826378
40	1	0	-2.581701	-0.255736	-2.493016
41	1	0	-0.863105	0.127372	-2.561444
42	1	0	-1.642188	-0.694556	-3.914681
43	6	0	0.355856	0.120912	2.750000
44	6	0	-0.152764	1.247348	2.534406
45	1	0	-0.417349	2.042148	3.213315
46	9	0	3.236016	-2.432312	0.902707
47	9	0	5.636927	-1.297639	0.573067
48	9	0	5.821292	1.285637	-0.229474
49	9	0	1.180689	1.604609	-0.437364
50	9	0	3.567827	2.708074	-0.719806

PRODUCT for *Re* attack in THF of 1b with 2a

Center Number	Atomic Number	Atomic Type	Coordinates (Angstroms) X	Y	Z
1	16	0	-1.493695	-1.787799	-0.890716
2	7	0	-0.639590	-0.700378	-0.014670
3	8	0	-1.478148	-3.126434	-0.207258
4	6	0	0.553866	-1.154841	0.668070
5	1	0	0.664397	-2.231483	0.544631
6	6	0	1.840697	-0.511780	0.166614
7	6	0	3.009244	-1.260607	0.132304
8	6	0	1.950103	0.799083	-0.268200
9	6	0	4.211390	-0.759544	-0.331555
10	6	0	3.136337	1.333539	-0.739633
11	6	0	4.274560	0.549482	-0.773959
12	12	0	-1.604622	1.024482	0.362375
13	35	0	-2.247511	3.052248	-0.868504
14	8	0	-3.248015	0.434110	1.413391
15	6	0	-4.572755	1.018362	1.310797
16	6	0	-5.390643	0.317841	2.378965
17	6	0	-4.757122	-1.072394	2.412463
18	6	0	-3.282870	-0.761003	2.237889
19	1	0	-6.448773	0.300669	2.130339
20	1	0	-2.724321	-1.540179	1.724788
21	1	0	-4.949181	-1.608748	3.338330
22	1	0	-5.116878	-1.677126	1.580093
23	1	0	-5.267971	0.816824	3.340440
24	1	0	-4.474479	2.091100	1.452801
25	1	0	-2.798618	-0.522086	3.185369
26	1	0	-4.950949	0.818987	0.308277
27	6	0	0.405545	-0.934463	2.193119
28	1	0	1.338876	-1.158500	2.710951
29	1	0	-0.353501	-1.626037	2.559038
30	6	0	-0.542664	-2.076530	-2.475661
31	6	0	-1.511929	-2.877544	-3.346072
32	1	0	-1.064760	-3.049616	-4.326644
33	1	0	-1.733301	-3.845184	-2.896521
34	1	0	-2.450732	-2.340952	-3.494396
35	6	0	0.728509	-2.873443	-2.224576
36	1	0	1.119715	-3.243236	-3.174450
37	1	0	1.504403	-2.262072	-1.769386
38	1	0	0.524426	-3.729001	-1.581288
39	6	0	-0.259150	-0.714773	-3.095237
40	1	0	-1.173471	-0.128040	-3.206982
41	1	0	0.440076	-0.140508	-2.489524
42	1	0	0.174403	-0.847868	-4.088298
43	6	0	-0.007840	0.430989	2.505285
44	6	0	-0.364532	1.556588	2.723579
45	1	0	-0.644405	2.556173	2.963545
46	9	0	2.988462	-2.529655	0.547334
47	9	0	5.296717	-1.522064	-0.357518
48	9	0	5.416687	1.049989	-1.220035
49	9	0	0.895888	1.630116	-0.254745
50	9	0	3.187107	2.593327	-1.151500

RC for *Re* attack in DCM of 1a with 2b

Center Number	Atomic Number	Atomic Type	Coordinates (Angstroms) X	Y	Z
1	12	0	1.807659	0.815041	1.294378
2	6	0	0.094691	0.763026	2.612256
3	6	0	-0.517636	-0.537733	2.693562
4	6	0	-0.989892	-1.650533	2.707015
5	6	0	-0.540527	-1.218928	-0.272330
6	7	0	0.653413	-0.761220	-0.324605
7	1	0	-0.633209	1.515930	2.298253
8	1	0	0.470615	1.063458	3.594621

9	1	0	-0.743106	-2.272684	-0.099631
10	16	0	1.888086	-1.869734	0.054840
11	8	0	2.730254	-0.996535	0.968568
12	6	0	-1.713538	-0.378793	-0.460473
13	6	0	-2.973532	-0.959594	-0.305610
14	6	0	-1.673730	0.984307	-0.760855
15	6	0	-4.138676	-0.231897	-0.423496
16	6	0	-2.830371	1.729930	-0.883129
17	6	0	-4.063313	1.121449	-0.712099
18	35	0	3.065710	2.776416	0.396106
19	6	0	2.771988	-1.914868	-1.566543
20	6	0	3.126063	-0.507350	-2.020372
21	1	0	3.728556	0.010941	-1.275888
22	1	0	3.709862	-0.580935	-2.938608
23	1	0	2.236968	0.085563	-2.225407
24	6	0	1.837922	-2.628237	-2.540104
25	1	0	1.507002	-3.593683	-2.153779
26	1	0	0.964851	-2.021909	-2.777557
27	1	0	2.381289	-2.811464	-3.467070
28	6	0	4.023227	-2.746578	-1.281732
29	1	0	3.772323	-3.750419	-0.936733
30	1	0	4.592914	-2.840547	-2.206190
31	1	0	4.657586	-2.264215	-0.539217
32	6	0	-1.616751	-2.969910	2.819084
33	1	0	-1.149942	-3.704005	2.159605
34	1	0	-1.538225	-3.349638	3.838803
35	1	0	-2.676412	-2.924457	2.566069
36	9	0	-2.767236	3.021641	-1.168104
37	9	0	-5.168232	1.831073	-0.829859
38	9	0	-5.318634	-0.812151	-0.264468
39	9	0	-3.073487	-2.254795	-0.028582
40	9	0	-0.525044	1.611326	-0.955467

TS for *Re* attack in DCM of 1a with 2b

Frequency -214.275

Center Number	Atomic Number	Atomic Type	Coordinates (Angstroms) X	Y	Z
1	12	0	-1.195957	-1.072022	1.186858
2	6	0	0.286690	-0.941042	2.833340
3	6	0	1.283999	0.007394	2.591112
4	6	0	2.068530	0.868136	2.215407
5	6	0	1.043296	1.057439	0.019568
6	7	0	-0.175394	0.593812	0.012236
7	1	0	0.661466	-1.961773	2.912485
8	1	0	-0.362582	-0.687118	3.672162
9	1	0	1.233967	2.111679	0.184229
10	16	0	-1.375651	1.669892	0.526791
11	8	0	-2.285647	0.692423	1.258696
12	6	0	2.138089	0.313709	-0.612599
13	6	0	3.202560	1.022211	-1.161726
14	6	0	2.185064	-1.072725	-0.714976
15	6	0	4.261855	0.393617	-1.788112
16	6	0	3.233762	-1.723584	-1.332742
17	6	0	4.276621	-0.987215	-1.872530
18	35	0	-2.328426	-2.983072	0.086442
19	6	0	-2.212781	2.013108	-1.087895
20	6	0	-2.598580	0.710626	-1.770563
21	1	0	-3.241033	0.103687	-1.134031
22	1	0	-3.151189	0.949223	-2.680096
23	1	0	-1.722848	0.125943	-2.045804
24	6	0	-1.227027	2.833184	-1.914748
25	1	0	-0.885687	3.721488	-1.380583
26	1	0	-0.360913	2.242863	-2.211779
27	1	0	-1.730141	3.165955	-2.822844
28	6	0	-3.443286	2.836198	-0.707218
29	1	0	-3.170116	3.758146	-0.192133
30	1	0	-3.976988	3.106122	-1.618716
31	1	0	-4.119007	2.263484	-0.073514
32	6	0	3.218368	1.780277	2.215076

33	1	0	2.920382	2.797579	1.957799
34	1	0	3.705734	1.807286	3.189836
35	1	0	3.962301	1.461727	1.482517
36	9	0	3.255627	-3.045828	-1.402571
37	9	0	5.284189	-1.604338	-2.462851
38	9	0	5.254589	1.101993	-2.305611
39	9	0	1.225746	-1.825061	-0.183347
40	9	0	3.211612	2.352599	-1.102987

PRODUCT for *Re* attack in DCM of 1a with 2b

Center Number	Atomic Number	Atomic Type	Coordinates (Angstroms) X	Y	Z
1	12	0	-1.962037	-0.328538	0.494381
2	6	0	-1.436526	-2.674506	1.868483
3	6	0	-0.228755	-2.175052	1.826008
4	6	0	0.971725	-1.681959	1.770249
5	6	0	1.223969	-0.323419	1.119953
6	7	0	0.025679	0.284873	0.586754
7	1	0	-1.795521	-3.339335	1.091681
8	1	0	-2.093437	-2.485456	2.710547
9	1	0	1.662118	0.301391	1.903926
10	16	0	-0.386678	1.738056	1.183912
11	8	0	-1.926021	1.564987	1.251220
12	6	0	2.285379	-0.453161	0.034727
13	6	0	3.519991	0.161616	0.149201
14	6	0	2.039071	-1.147006	-1.140877
15	6	0	4.470996	0.109782	-0.855887
16	6	0	2.968128	-1.217177	-2.162184
17	6	0	4.191535	-0.583908	-2.018307
18	35	0	-3.747362	-1.403638	-0.801111
19	6	0	-0.174512	2.927300	-0.222369
20	6	0	-0.937309	2.441901	-1.443061
21	1	0	-2.008781	2.399283	-1.249967
22	1	0	-0.770982	3.138438	-2.266201
23	1	0	-0.588064	1.458980	-1.760408
24	6	0	1.326548	2.984735	-0.488947
25	1	0	1.894698	3.191778	0.420342
26	1	0	1.683044	2.049413	-0.918679
27	1	0	1.534432	3.784629	-1.200520
28	6	0	-0.703414	4.268304	0.279426
29	1	0	-0.167967	4.601731	1.170004
30	1	0	-0.565183	5.022784	-0.496341
31	1	0	-1.765915	4.207942	0.512055
32	6	0	2.162350	-2.392832	2.355558
33	1	0	2.682179	-1.737991	3.057280
34	1	0	1.866269	-3.298899	2.877374
35	1	0	2.870647	-2.661089	1.570015
36	9	0	3.822218	0.853736	1.251000
37	9	0	5.640619	0.721481	-0.711666
38	9	0	5.090080	-0.645905	-2.990378
39	9	0	2.699377	-1.889007	-3.275234
40	9	0	0.876679	-1.774066	-1.317745

X. References.

[1] Llobat, A.; Escorihuela, J.; Sedgwick, D. M.; Rodenes, M.; Román, R.; Soloshonok, V. A.; Han, J.; Medio-Simón, M.; Fustero, S. The Ruthenium-Catalyzed Domino Cross Enyne Metathesis/Ring-Closing Metathesis in the Synthesis of Enantioen-riched Nitrogen-Containing Heterocycles. *Eur. J. Org. Chem*. **2020**, 4193−4207.
[2] Dolomanov, O.V.; Bourhis, L.J.; Gildea, R.J.; Howard, J. A. K.; Puschmann, H. OLEX2: a complete structure solution, refinement and analysis program. *J. Appl. Cryst*. **2009**, *42*, 339−341.
[3] Sheldrick, G.M. A short history of SHELX. *Acta Cryst.* **2008**, *A64*, 112−122.
[4] Sheldrick, G.M. Crystal structure refinement with SHELXL. *Acta Cryst*. **2015**, *C71*, 3−8.
[5] Chai, J.-D.; Head-Gordon, M. Long-range corrected hybrid density functionals with damped atom–atom dispersion corrections. Phys. Chem. Chem. Phys. 2008, 10, 6615−6620.
[6] Gaussian 16, Revision B.01, Frisch, M. J.; Trucks, G. W.; Schlegel, H. B.; Scuseria, G. E.; Robb, M. A.; Cheeseman, J. R.; Scalmani, G.; Barone, V.; Petersson, G. A.; Nakatsuji, H.; Li, X.; Caricato, M.; Marenich, A. V.; Bloino, J.; Janesko, B. G.; Gomperts, R.; Mennucci, B.; Hratchian, H. P.; Ortiz, J. V.; Izmaylov, A. F.; Sonnenberg, J. L.; Williams-Young, D.; Ding, F.; Lipparini, F.; Egidi, F.; Goings, J.; Peng, B.; Petrone, A.; Henderson, T.; Ranasinghe, D.; Zakrzewski, V. G.; Gao, J.; Rega, N.; Zheng, G.; Liang, W.; Hada, M.; Ehara, M.; Toyota, K.; Fukuda, R.; Hasegawa, J.; Ishida, M.; Nakajima, T.; Honda, Y.; Kitao, O.; Nakai, H.; Vreven, T.; Throssell, K.; Mont-gomery, J. A., Jr.; Peralta, J. E.; Ogliaro, F.; Bearpark, M. J.; Heyd, J. J.; Brothers, E. N.; Kudin, K. N.; Staroverov, V. N.; Keith, T. A.; Kobayashi, R.; Normand, J.; Raghavachari, K.; Rendell, A. P.; Burant, J. C.; Iyengar, S. S.; Tomasi, J.; Cossi, M.; Millam, J. M.; Klene, M.; Adamo, C.; Cammi, R.; Ochterski, J. W.; Martin, R. L.; Morokuma, K.; Farkas, O.; Foresman, J. B.; Fox, D. J. Gaussian, Inc., Wallingford CT, 2016.
[7] Marenich, A. V.; Cramer, C. J.; Truhlar, D. G. Universal Solvation Model Based on Solute Electron Density and on a Continuum Model of the Solvent Defined by the Bulk Dielectric Constant and Atomic Surface Tensions. *J. Phys. Chem. B* **2009**, *113*, 6378−6396.
[8] a) Gonzalez, C.; Schlegel, H.B. An improved algorithm for reaction path following. J. Chem. Phys. 1989, 90, 2154; b) Gonzalez, C.; Schlegel, H.B. Reaction path following in mass-weighted internal coordinates. *J. Phys. Chem*. **1990**, *94*, 5523−5527.
[9] Weinhold, F. Natural bond orbital analysis: A critical overview of relationships to alternative bonding perspectives. *J. Comput. Chem*. **2012**, *33*, 2363−2379.
[10] Tao, J.; Perdew, J. P.; Staroverov, V. N.; Scuseria, G. E. Climbing the density functional ladder: Nonempirical meta-generalized gradi-ent approximation designed for molecules and solids. *Phys. Rev. Lett*. **2003**, *91*, 146401−146404.
[11] a) Weigend, F.; Ahlrichs, R. Balanced basis sets of split valence, triple zeta valence and quadruple zeta valence quality for H to Rn: Design and assessment of accuracy. Phys. Chem. Chem. Phys. 2005, 7, 3297−3305; b) Weigend, F. Accurate Coulomb-fitting basis sets for H to Rn. *Phys. Chem. Chem. Phys*. **2006**, *8*, 1057−1065.
[12] CYLview20; Legault, C. Y., Université de Sherbrooke, 2020 (http://www.cylview.org).

XI. ^{1}H, ^{13}C and ^{19}F NMR spectra of new compounds.

^{1}H NMR spectrum of compound **1a** (300 MHz, $CDCl_3$)

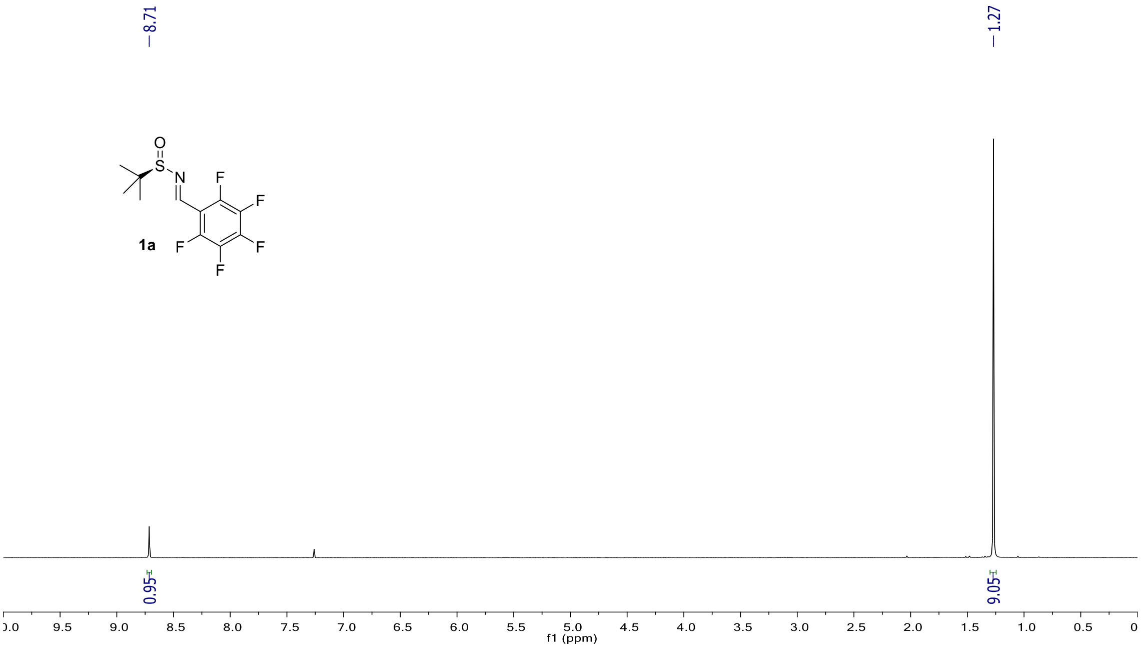

^{19}F NMR spectrum of compound **1a** (282 MHz, $CDCl_3$)

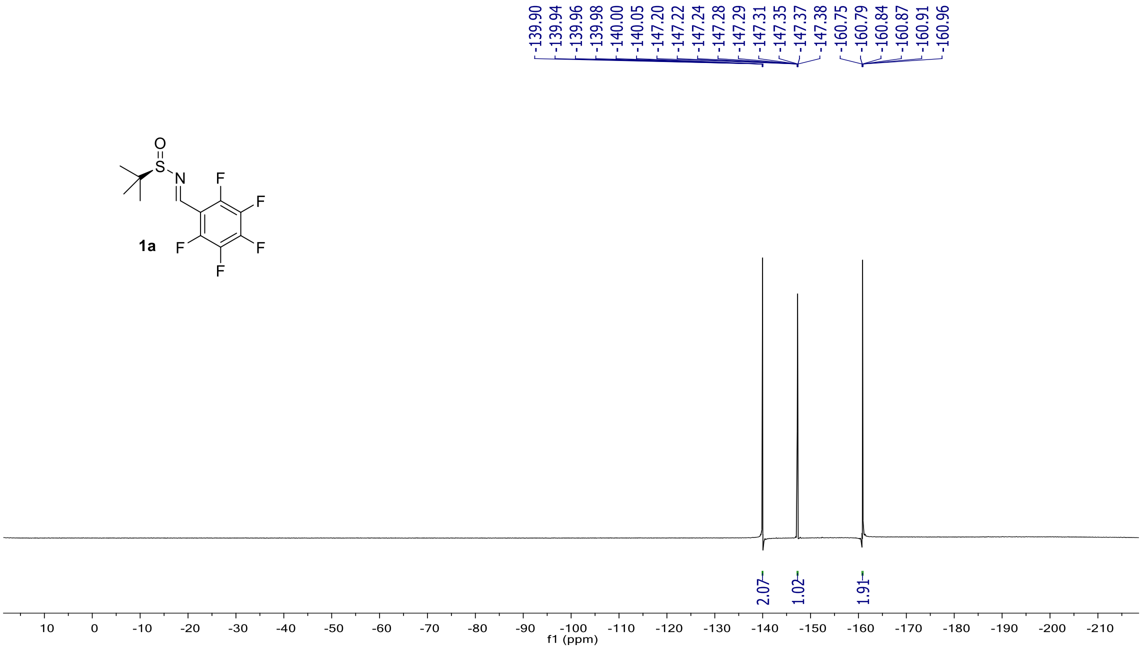

^{13}C NMR spectrum of compound **1a** (75 MHz, $CDCl_3$)

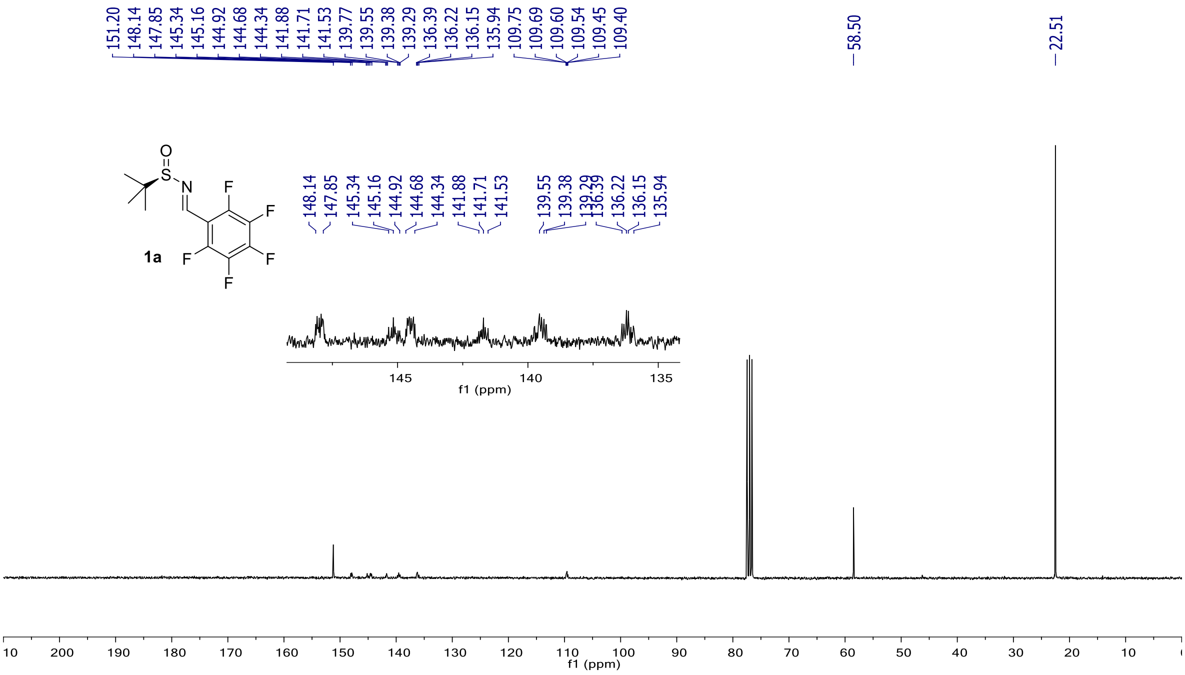

^{1}H NMR spectrum of compound **1b** (300 MHz, $CDCl_3$)

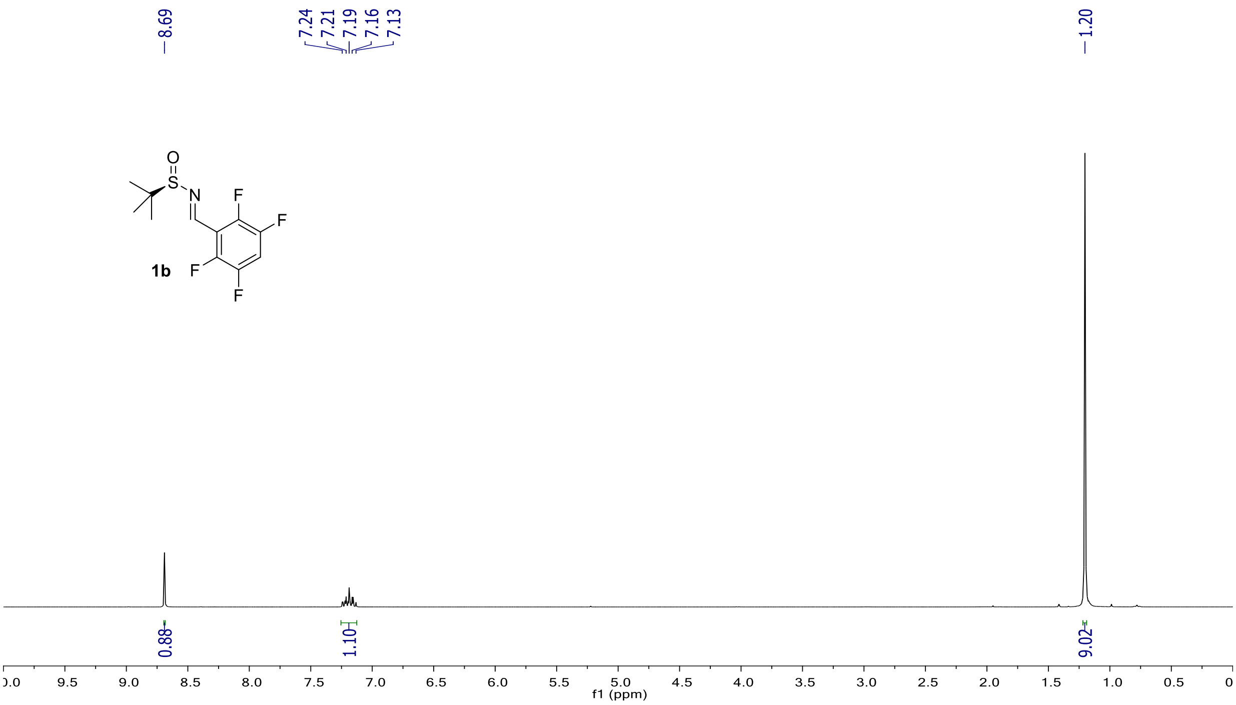

^{19}F NMR spectrum of compound **1b** (282 MHz, $CDCl_3$)

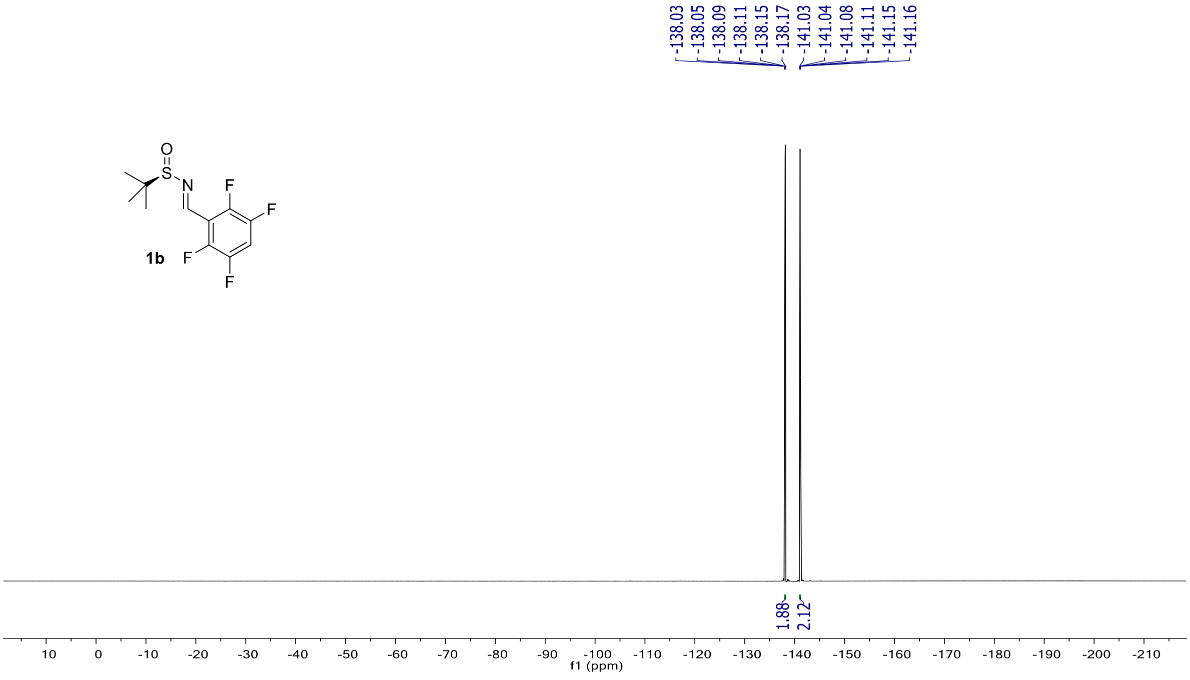

^{13}C NMR spectrum of compound **1b** (75 MHz, $CDCl_3$)

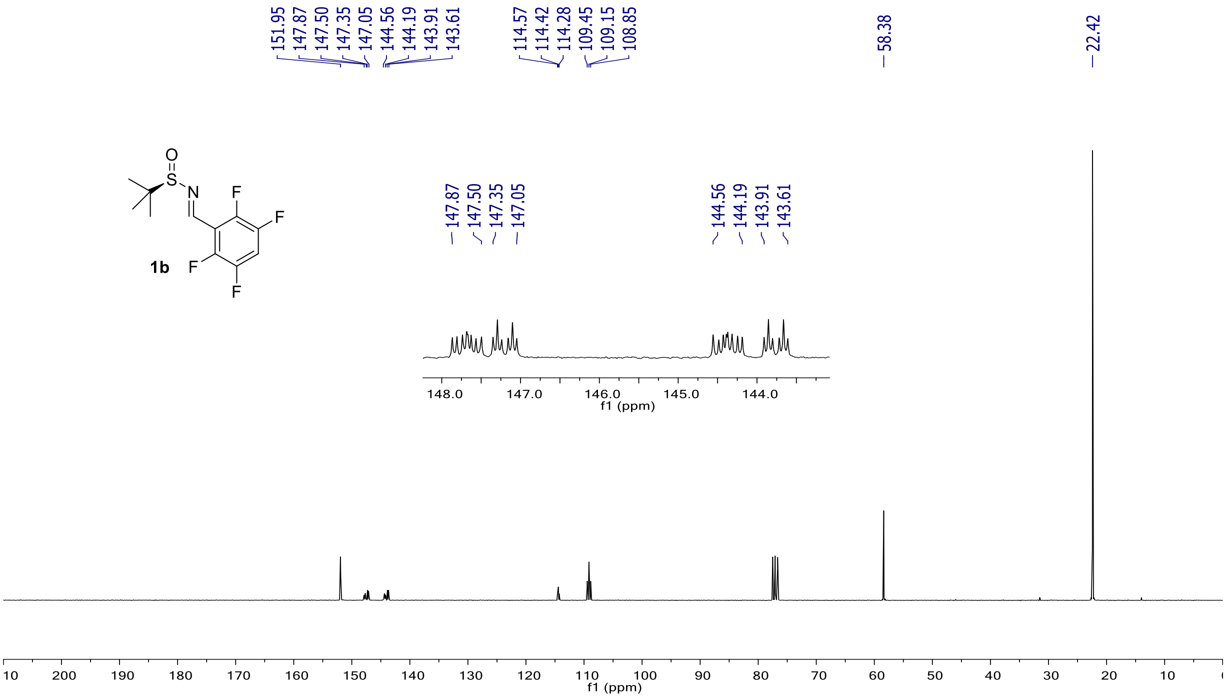

¹H NMR spectrum of compound **1c** (300 MHz, $CDCl_3$)

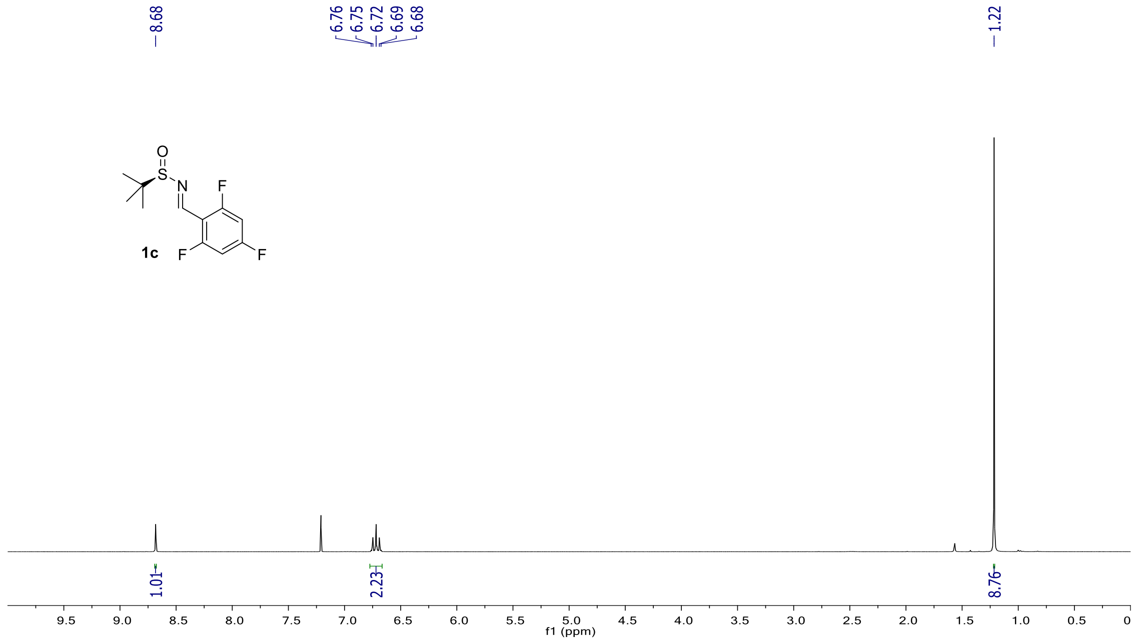

^{19}F NMR spectrum of compound **1c** (282 MHz, $CDCl_3$)

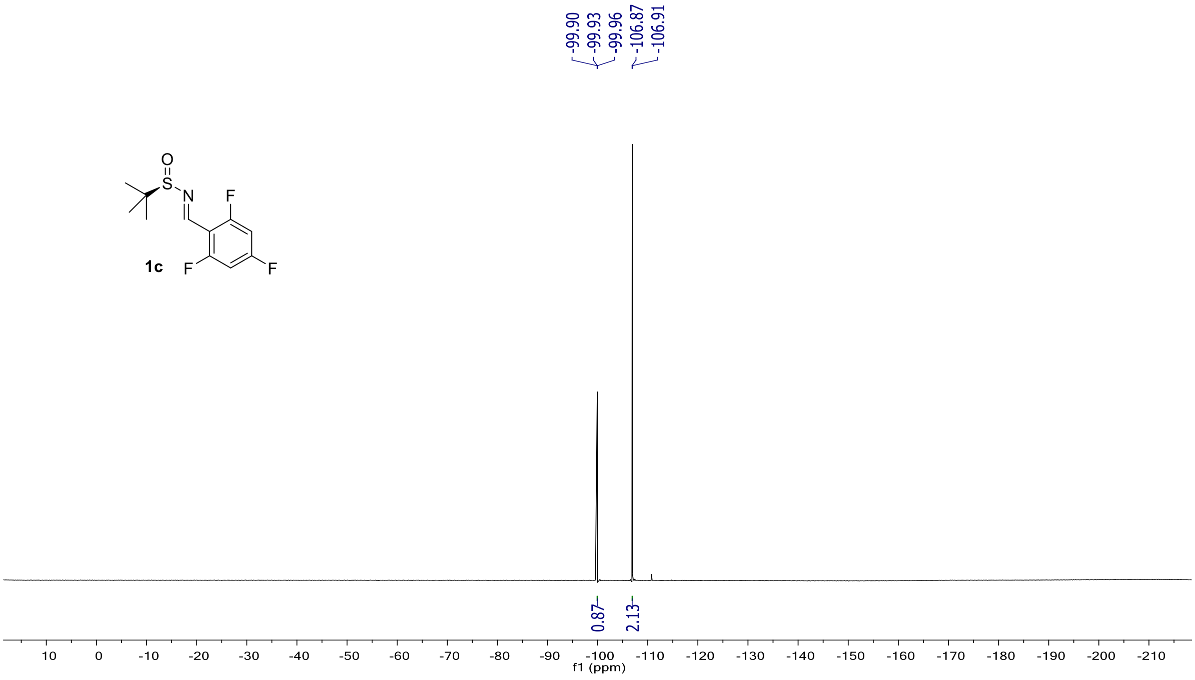

^{13}C NMR spectrum of compound **1c** (75 MHz, $CDCl_3$)

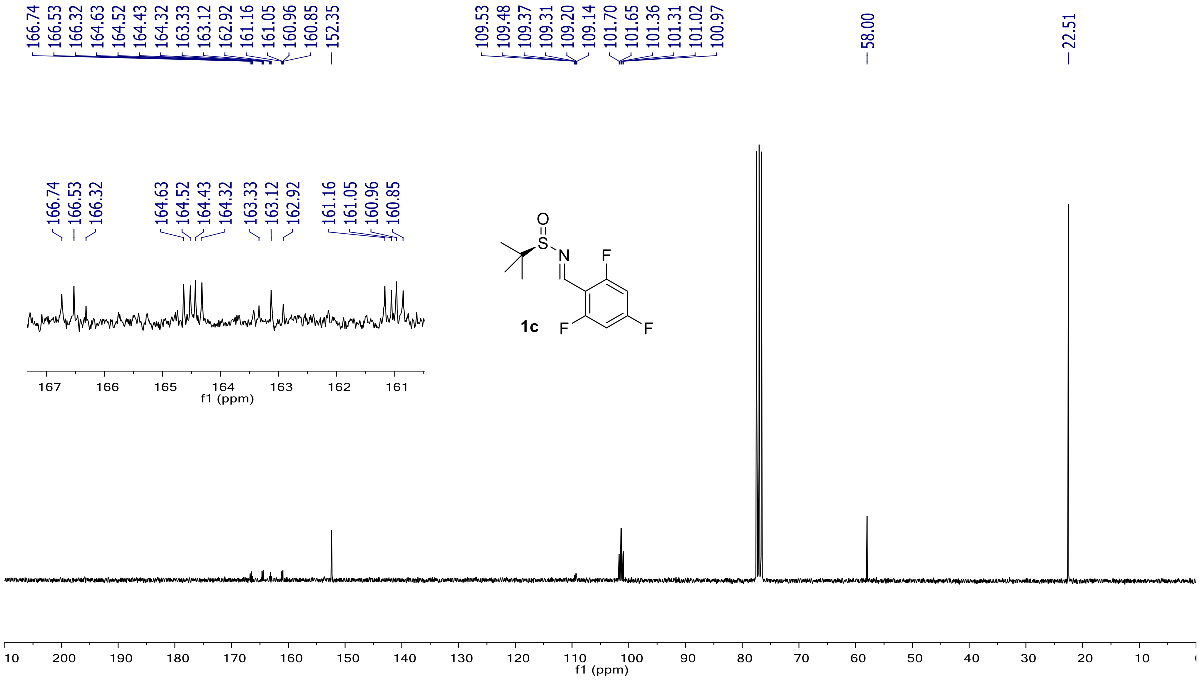

^{1}H NMR spectrum of compound **1d** (300 MHz, $CDCl_3$)

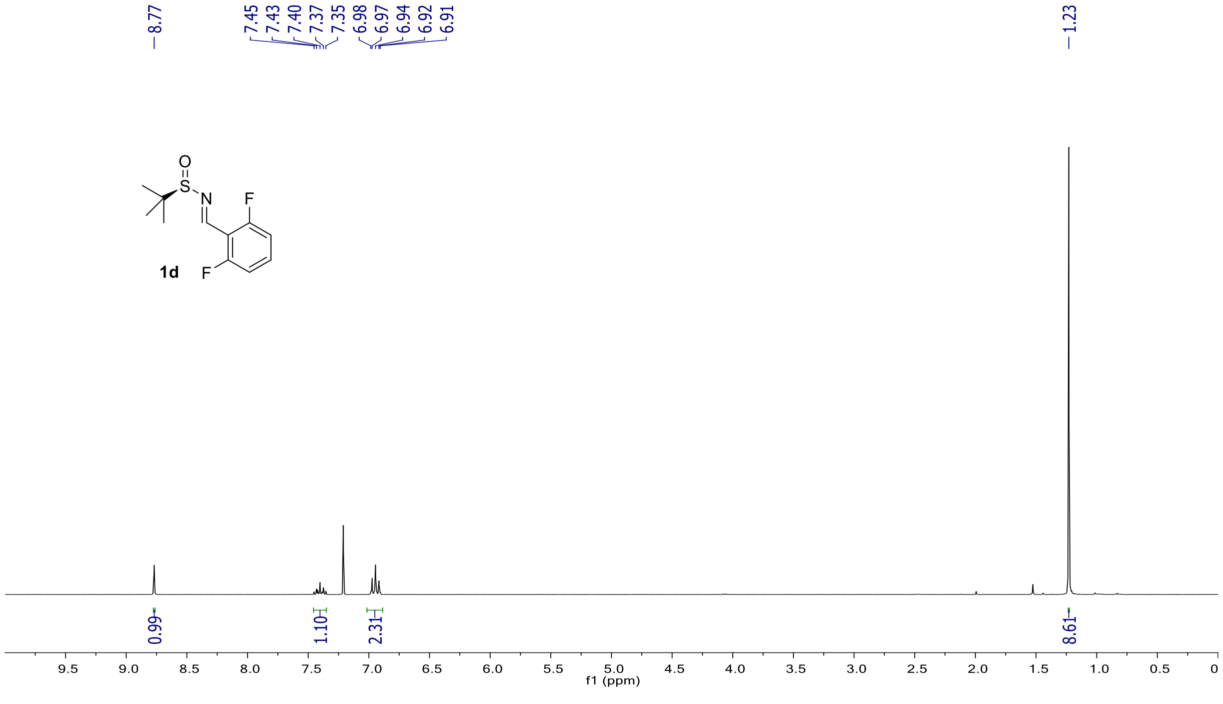

^{19}F NMR spectrum of compound **1d** (282 MHz, $CDCl_3$)

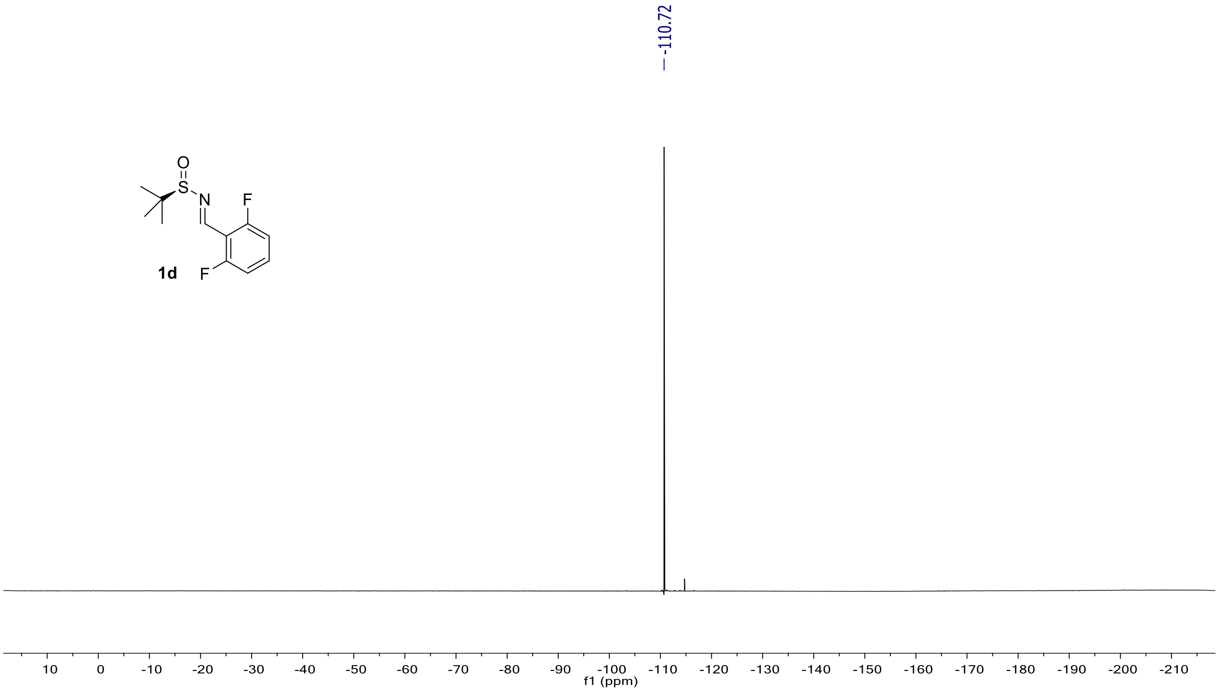

^{13}C NMR spectrum of compound **1d** (75 MHz, $CDCl_3$)

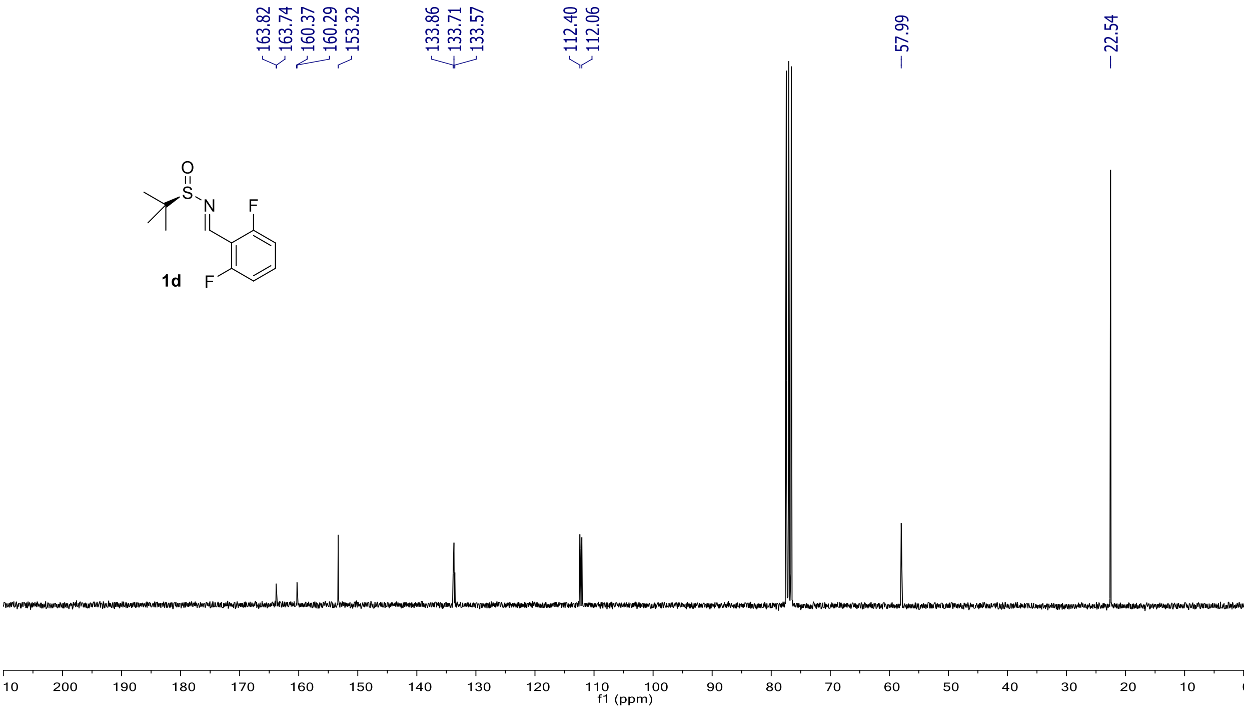

^{1}H NMR spectrum of compound **1e** (300 MHz, $CDCl_3$)

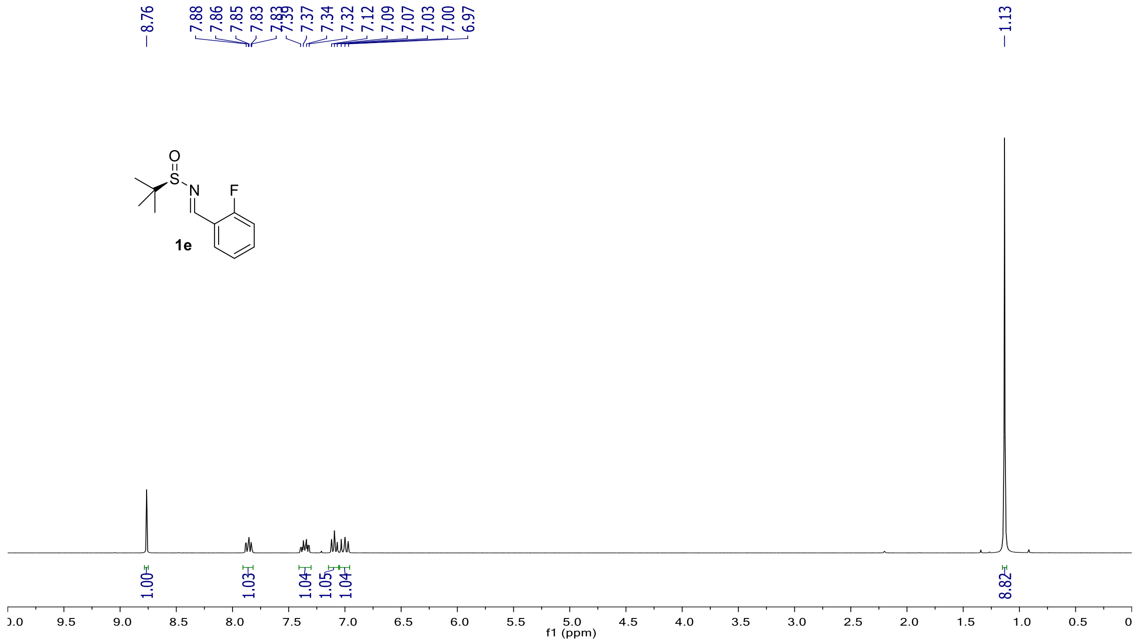

^{19}F NMR spectrum of compound **1e** (282 MHz, $CDCl_3$)

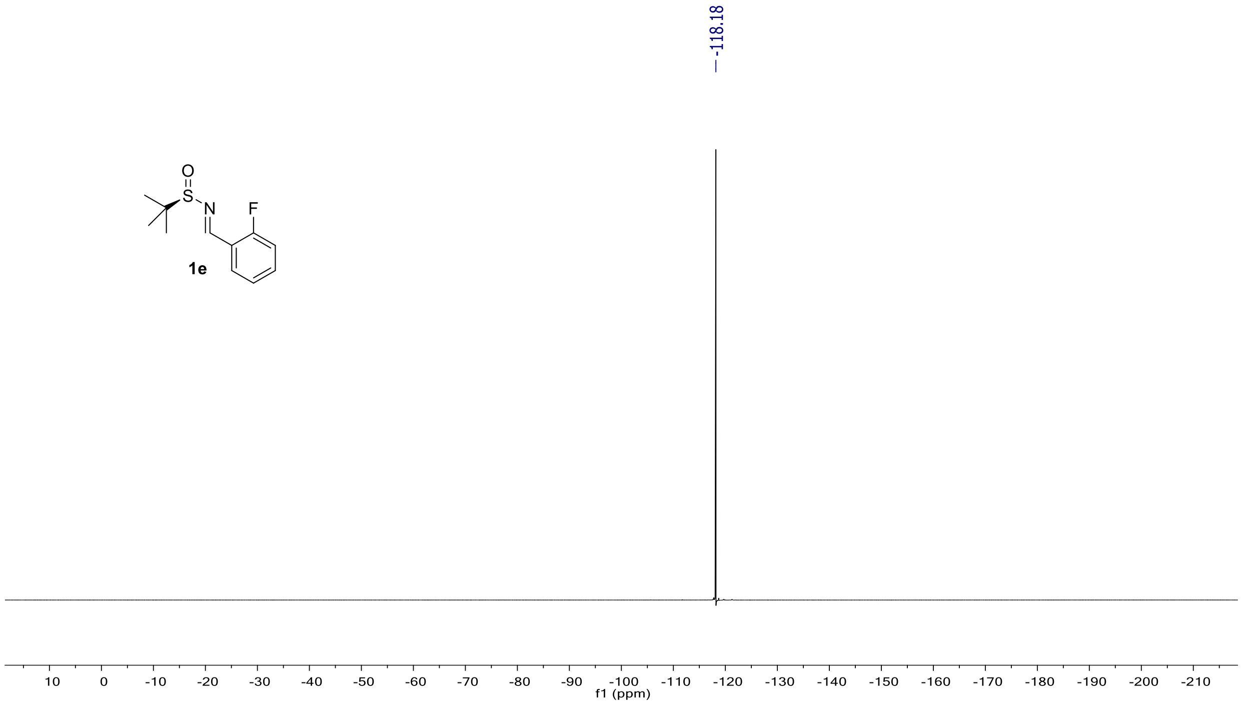

^{13}C NMR spectrum of compound **1e** (75 MHz, $CDCl_3$)

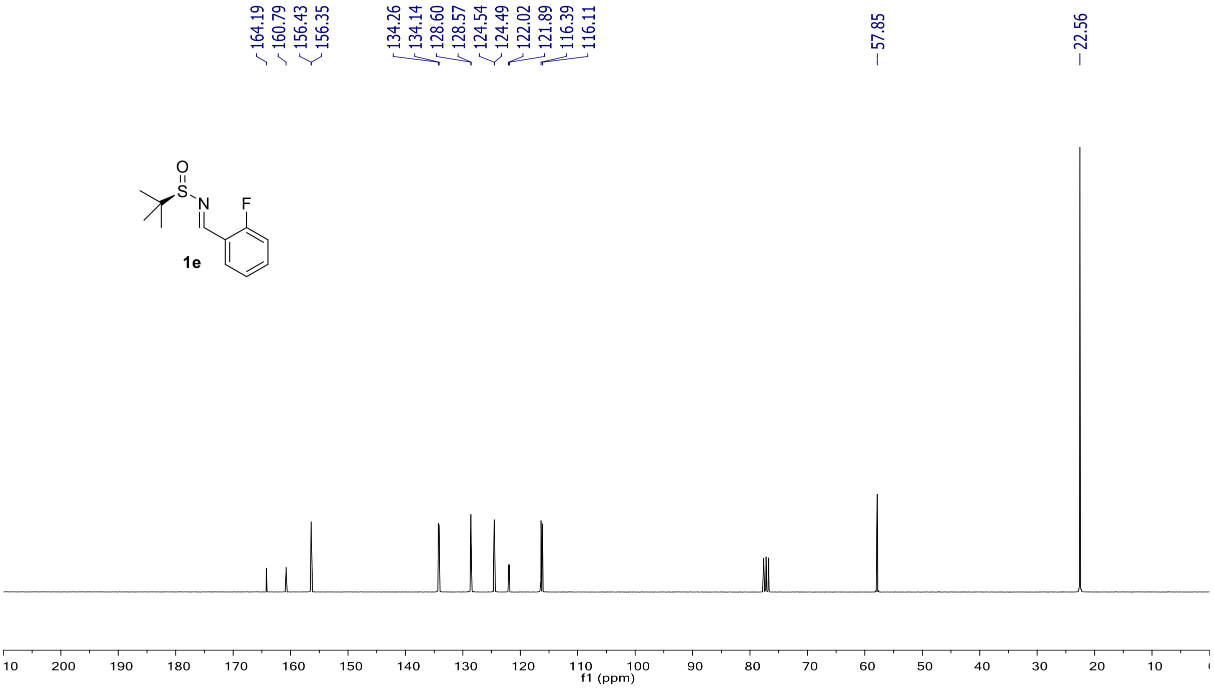

^{1}H NMR spectrum of compound **3a** (300 MHz, $CDCl_3$)

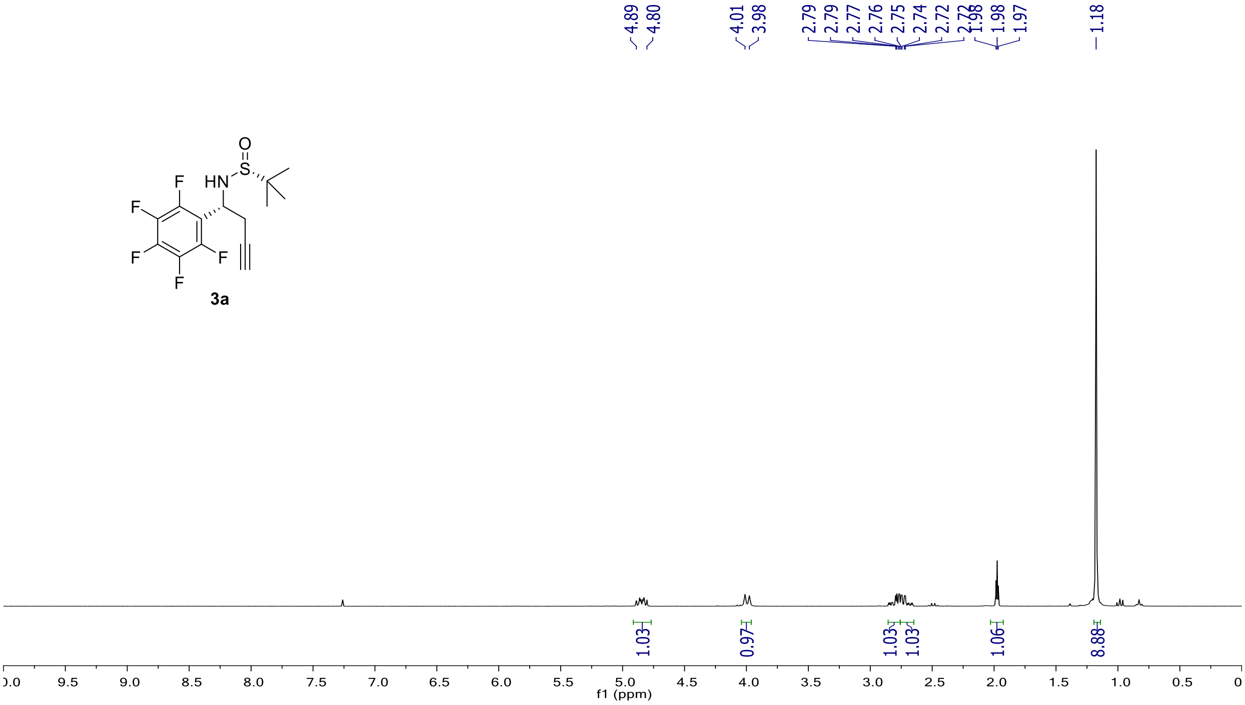

^{19}F NMR spectrum of compound **3a** (282 MHz, $CDCl_3$)

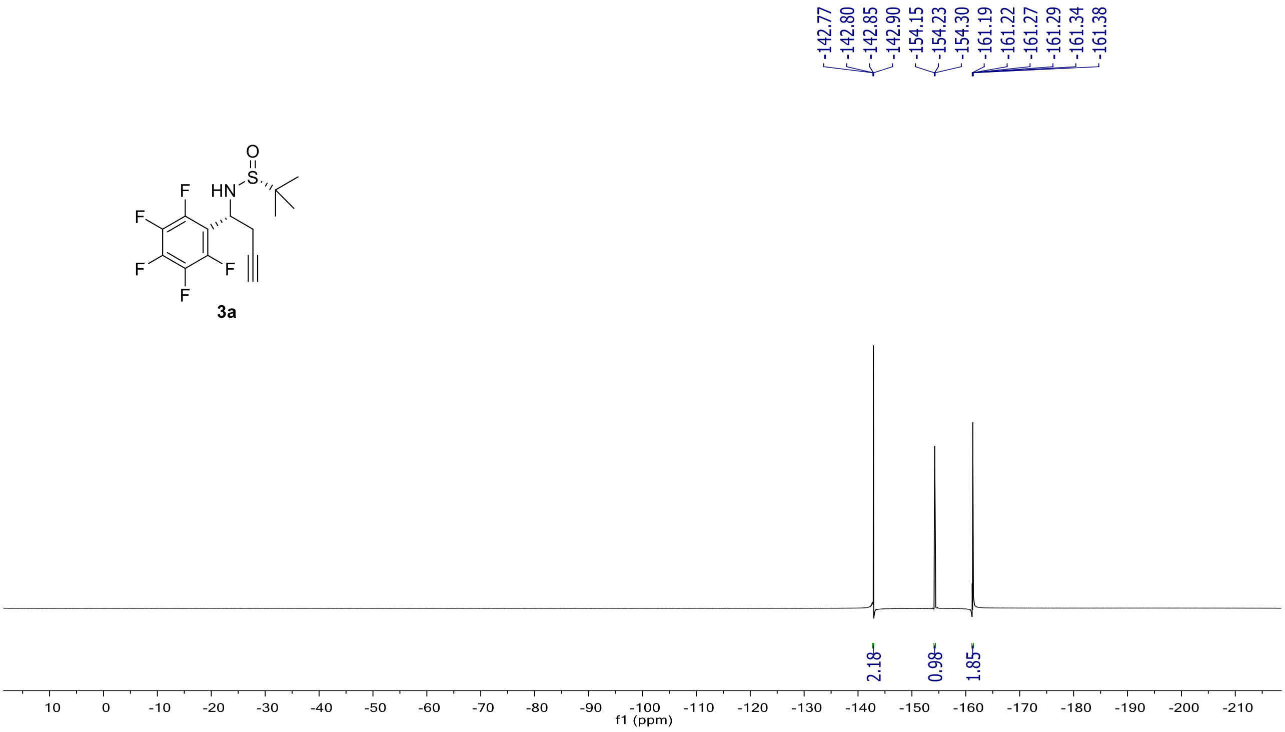

^{13}C NMR spectrum of compound **3a** (75 MHz, $CDCl_3$)

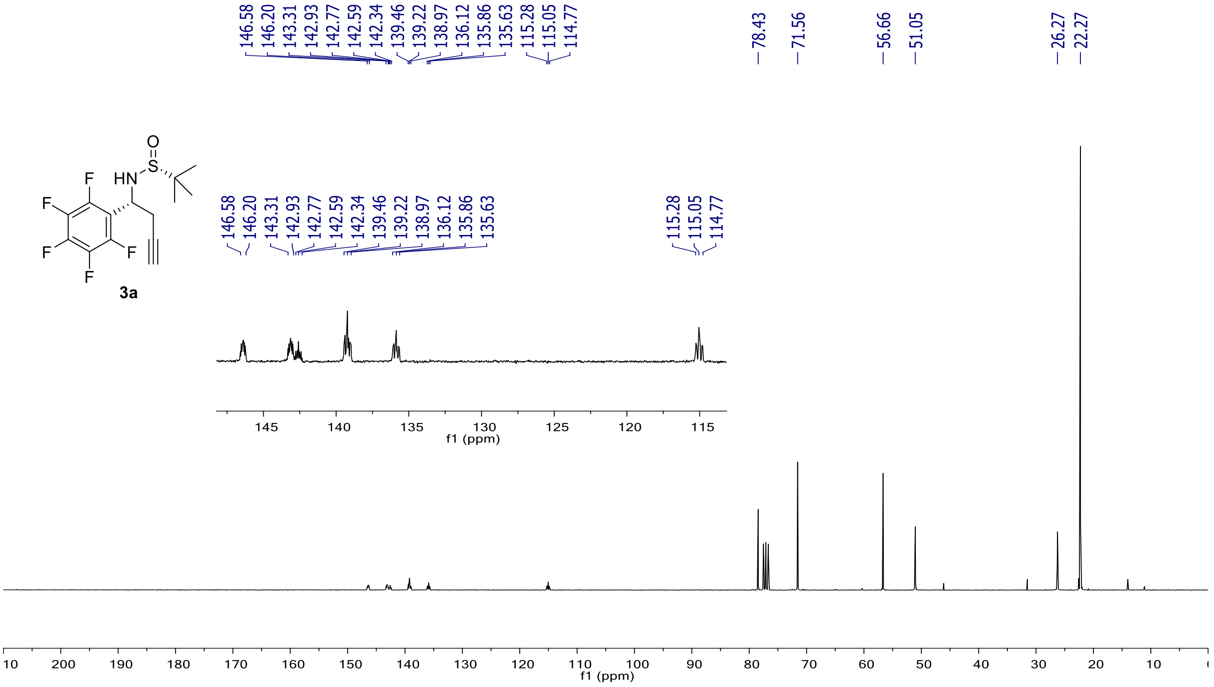

^{1}H NMR spectrum of compound **3’a** (300 MHz, $CDCl_3$)

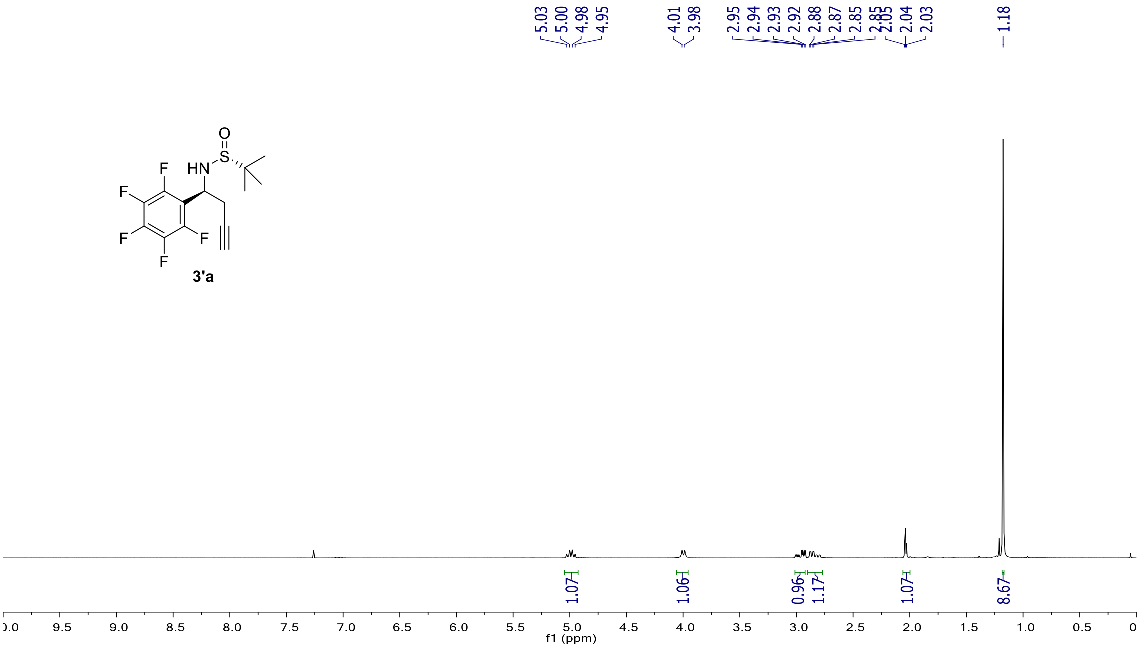

^{19}F NMR spectrum of compound **3'a** (282 MHz, $CDCl_3$)

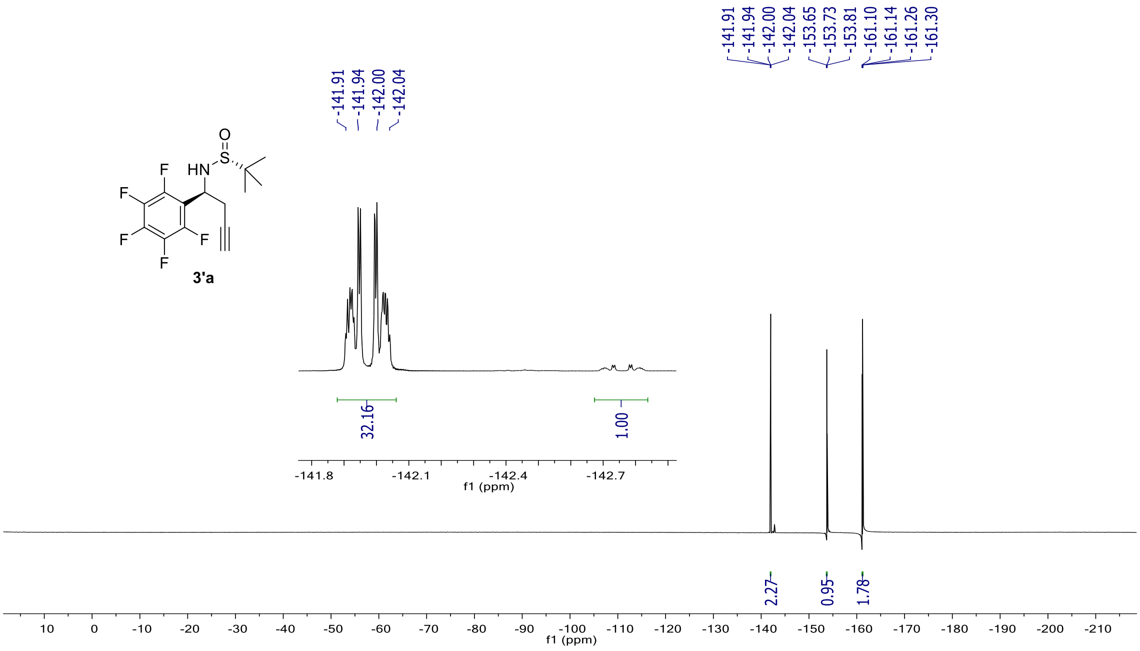

^{13}C NMR spectrum of compound **3'a** (75 MHz, $CDCl_3$)

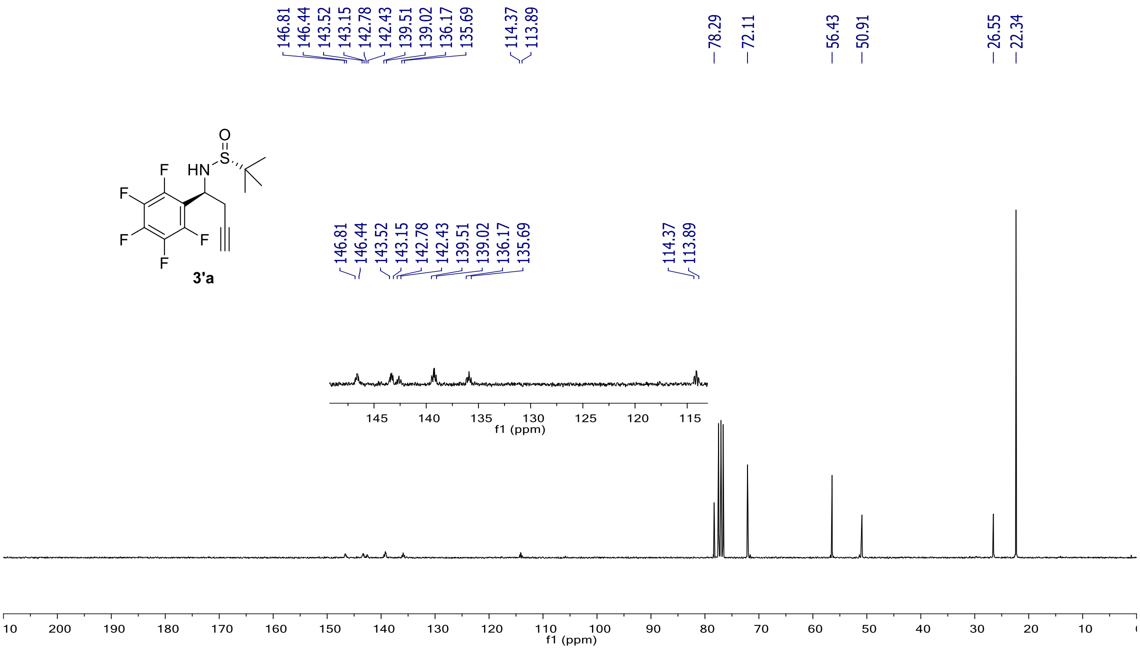

^{1}H NMR spectrum of compound **3b** (300 MHz, $CDCl_3$)

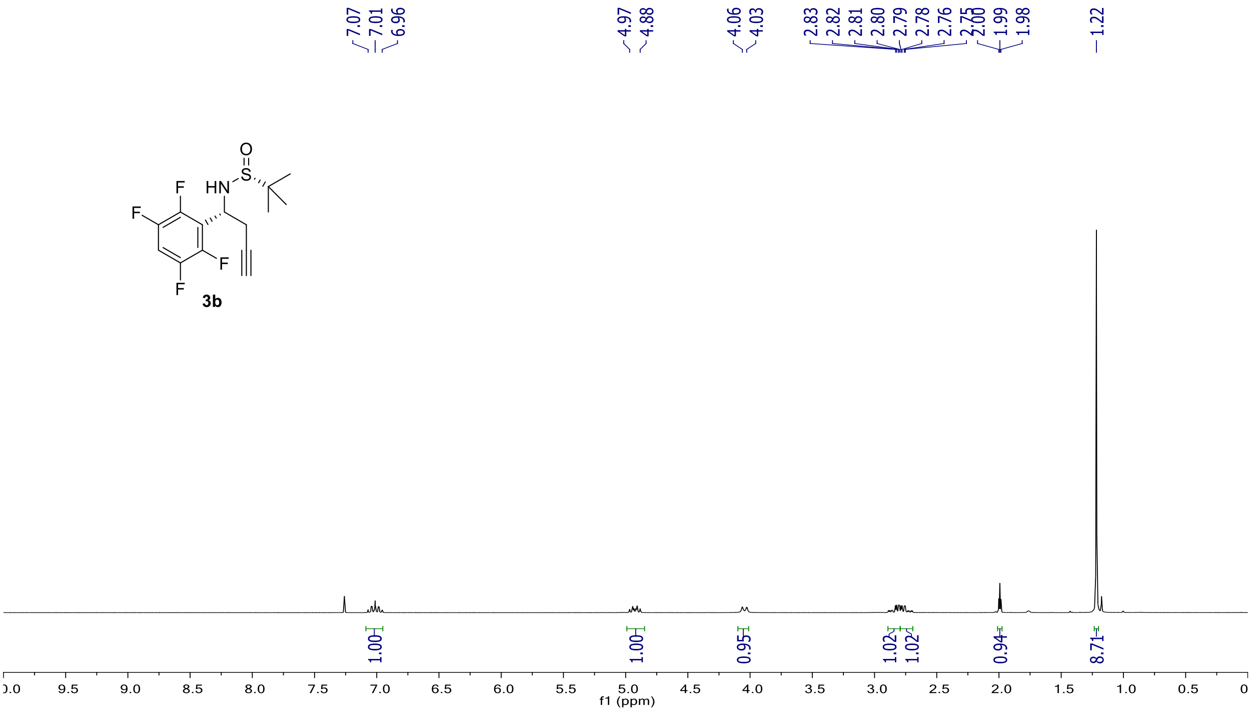

^{19}F NMR spectrum of compound **3b** (282 MHz, $CDCl_3$)

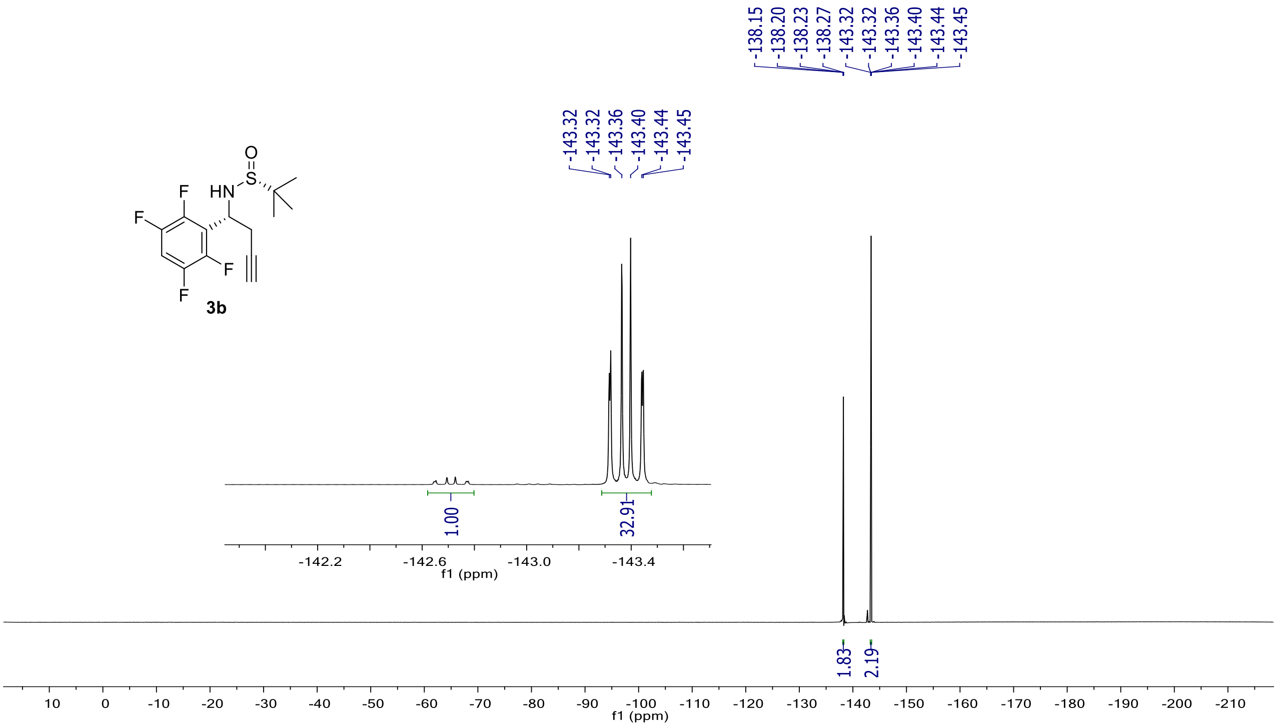

^{13}C NMR spectrum of compound **3b** (300 MHz, $CDCl_3$)

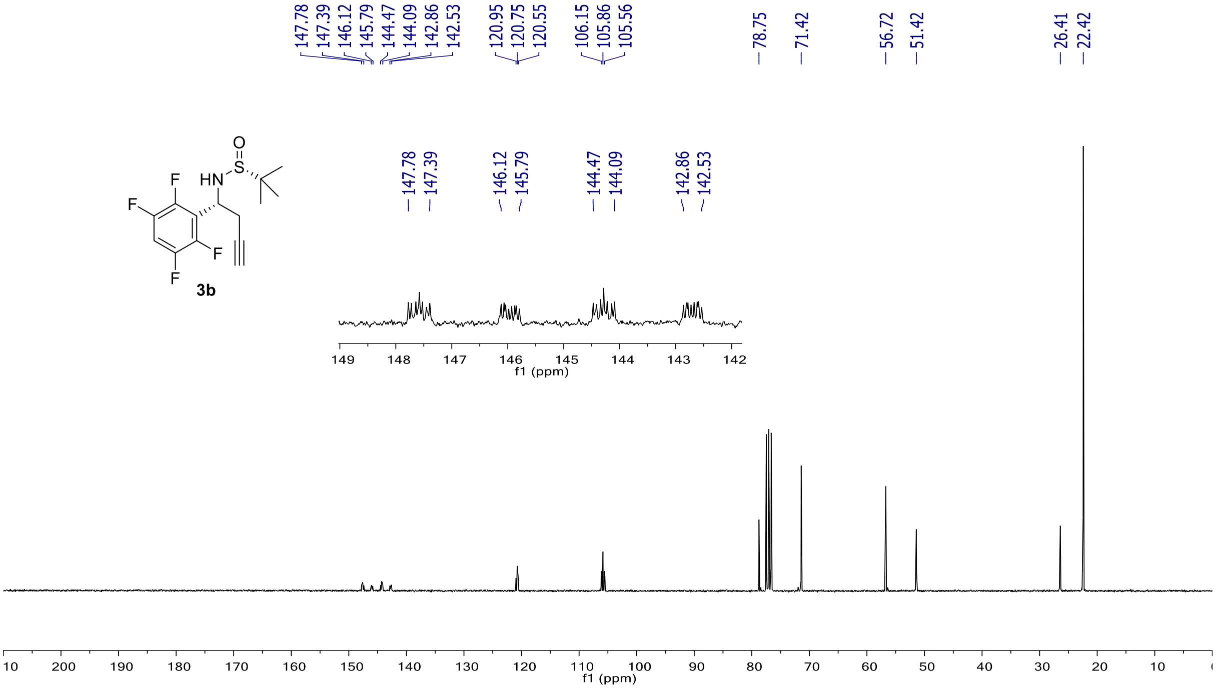

^{1}H NMR spectrum of compound **3’b** (300 MHz, $CDCl_3$)

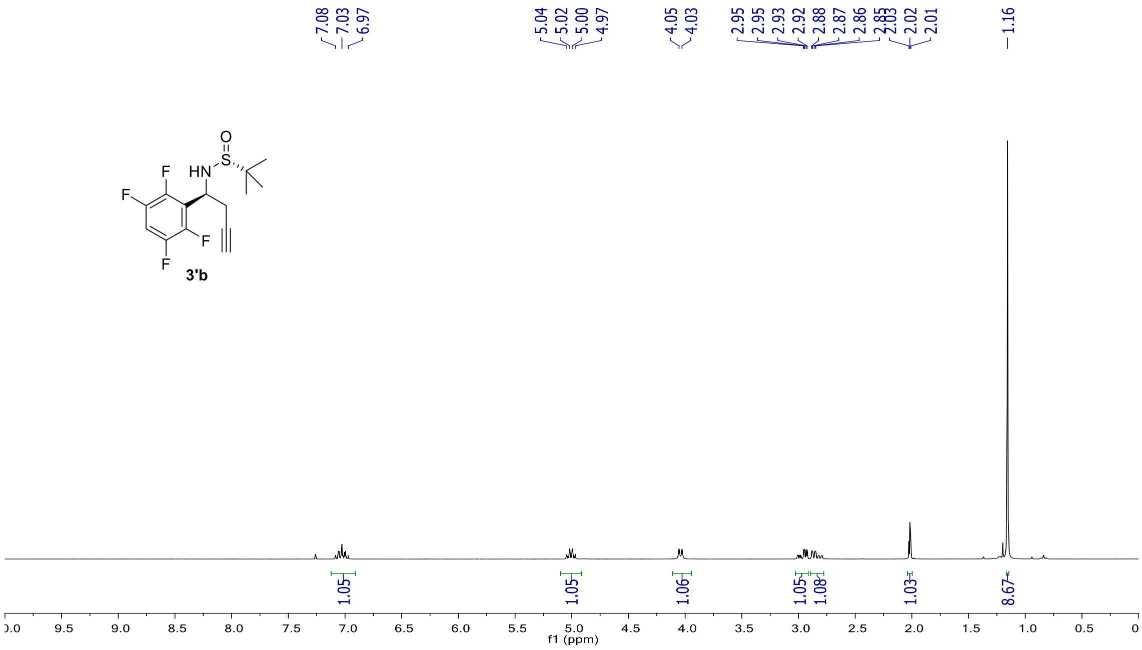

^{19}F NMR spectrum of compound **3’b** (282 MHz, $CDCl_3$)

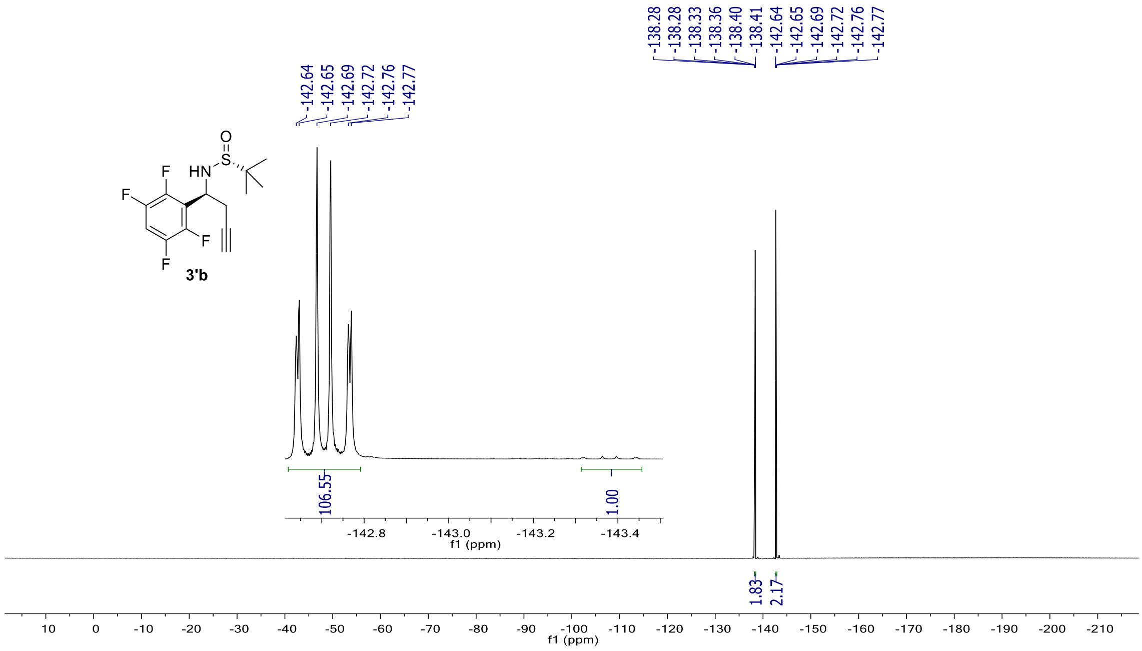

^{13}C NMR spectrum of compound **3’b** (75 MHz, $CDCl_3$)

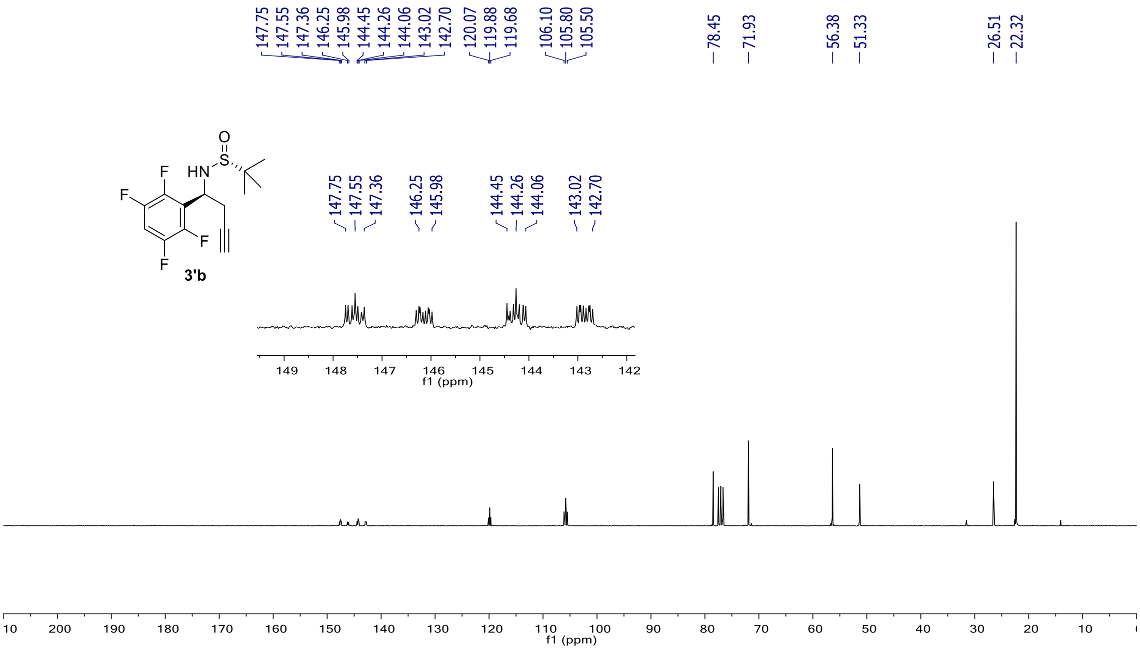

^{1}H NMR spectrum of compound **3’c** (300 MHz, $CDCl_3$)

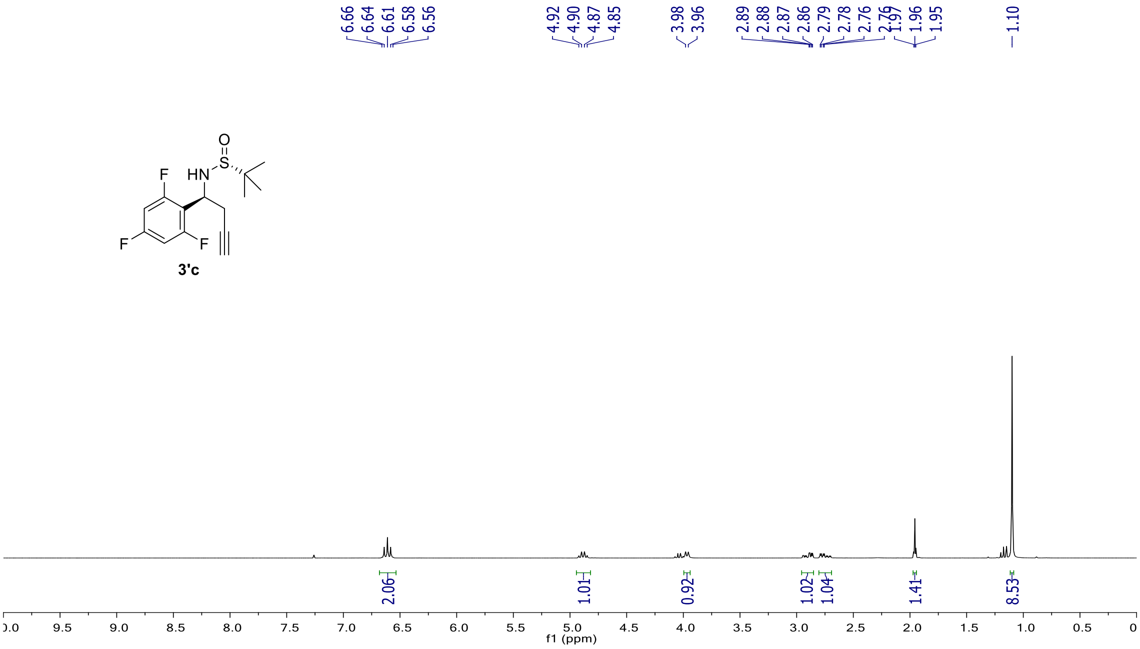

^{19}F NMR spectrum of compound **3'c** (282 MHz, $CDCl_3$)

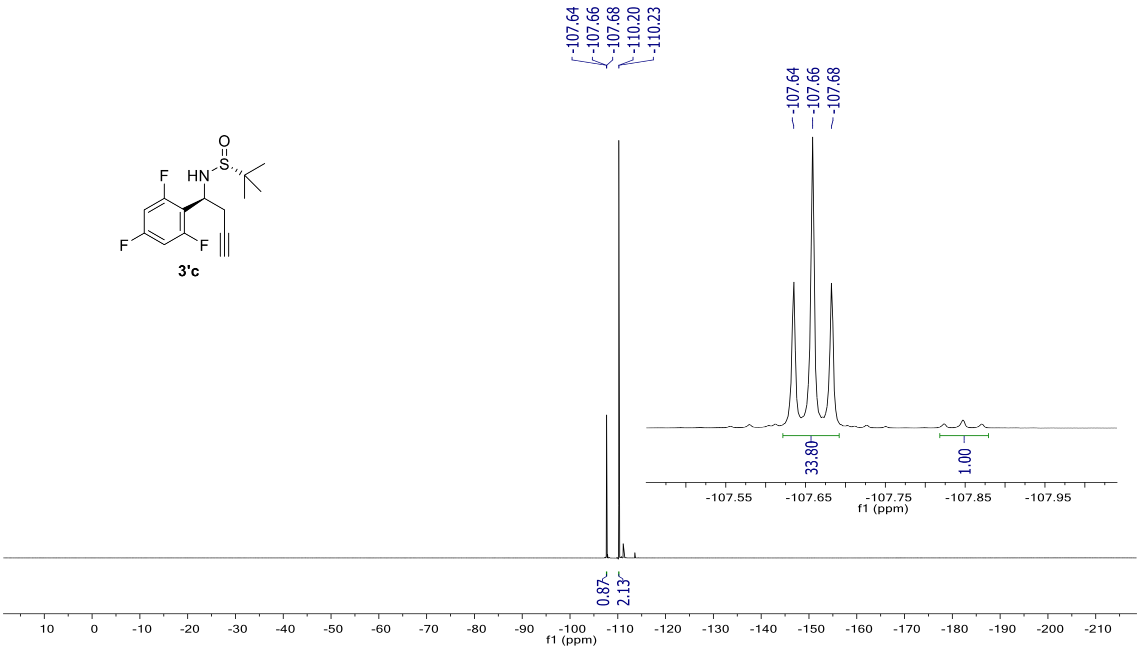

^{13}C NMR spectrum of compound **3'c** (75 MHz, $CDCl_3$)

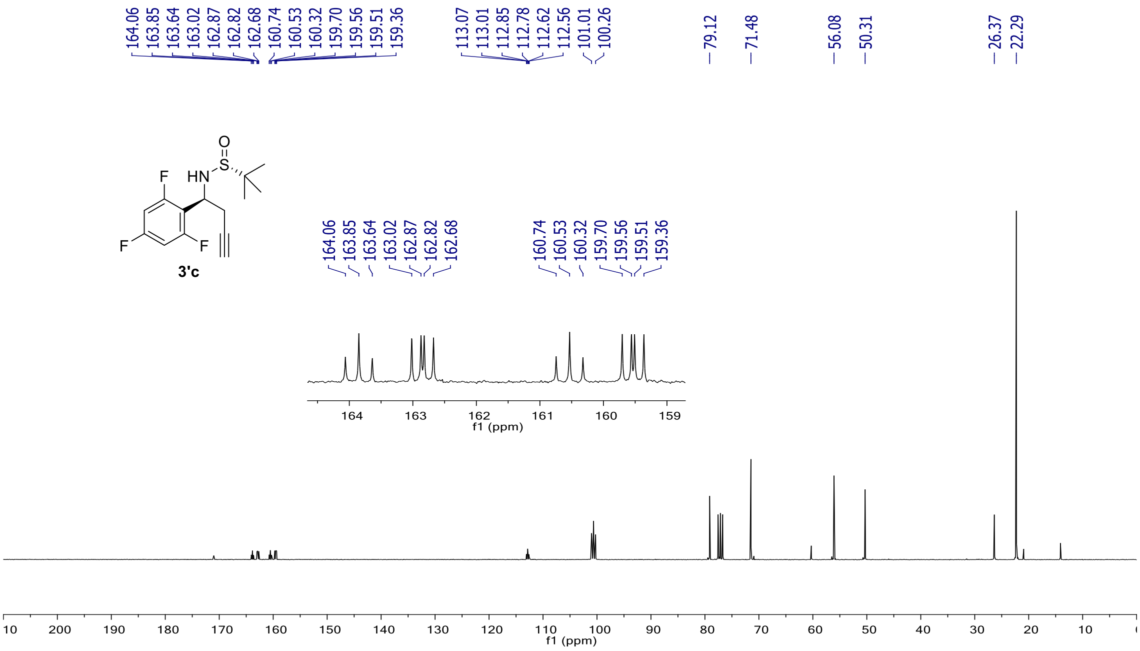

^{1}H NMR spectrum of compound **3’d** (300 MHz, $CDCl_3$)

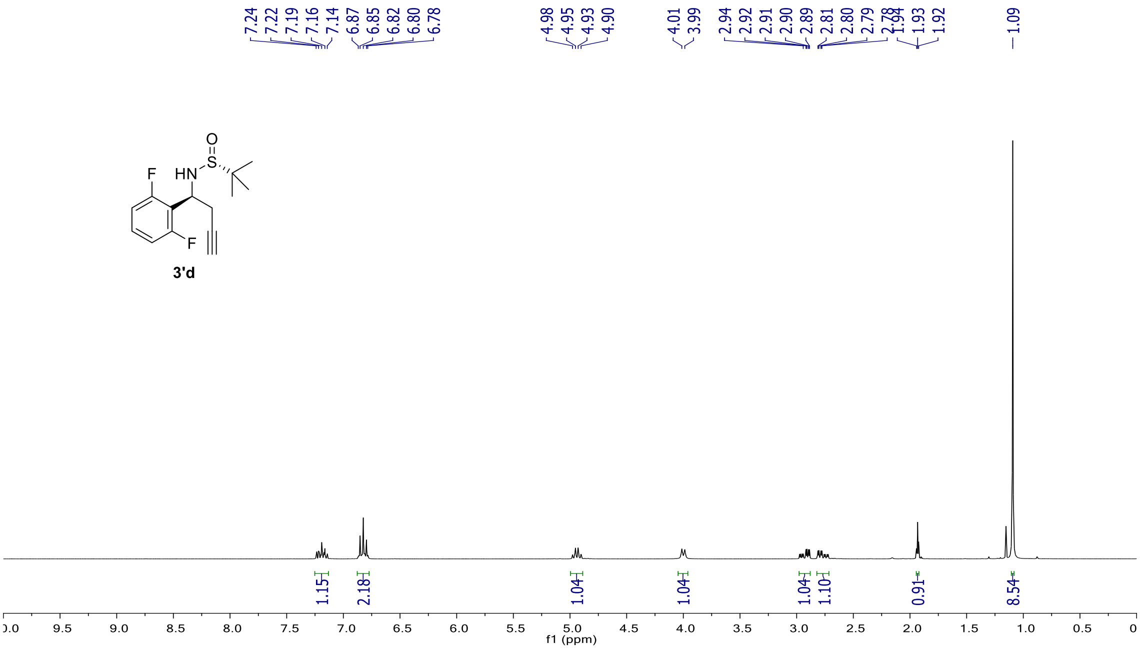

^{19}F NMR spectrum of compound **3’d** (282 MHz, $CDCl_3$)

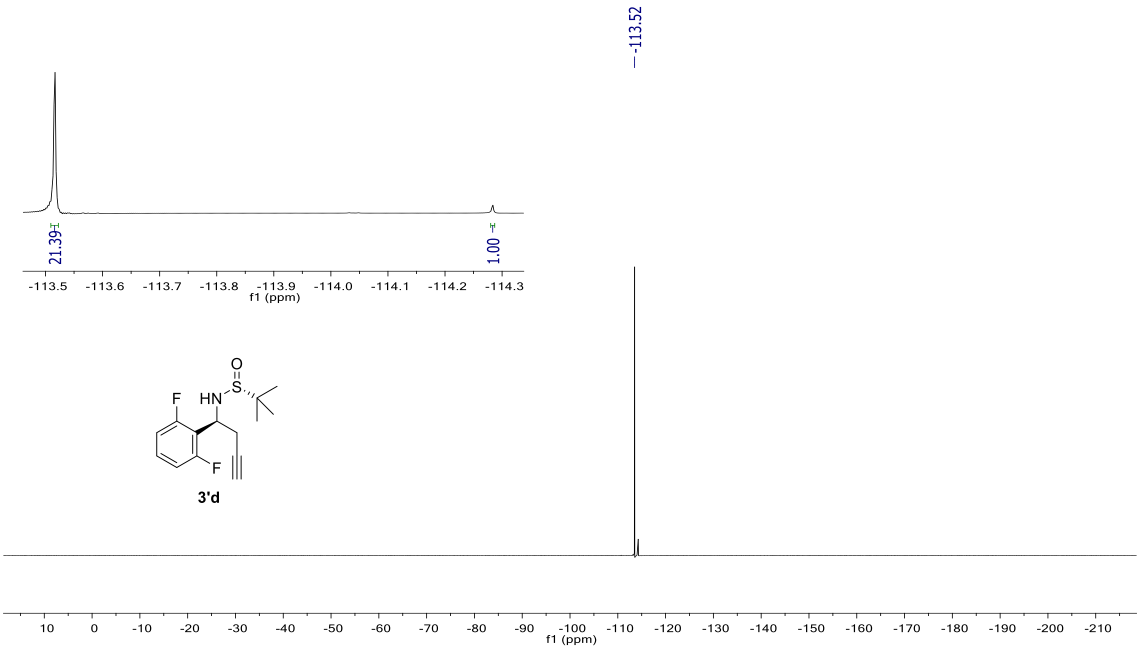

^{13}C NMR spectrum of compound **3’d** (75 MHz, $CDCl_3$)

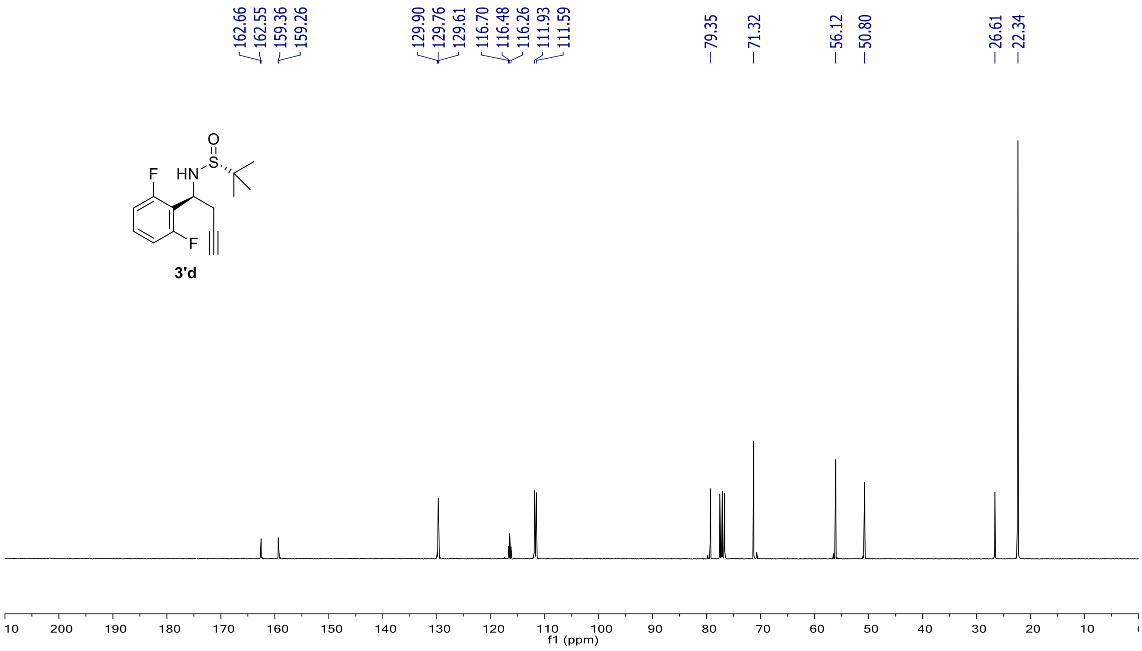

^{1}H NMR spectrum of compound **3’e** (300 MHz, $CDCl_3$)

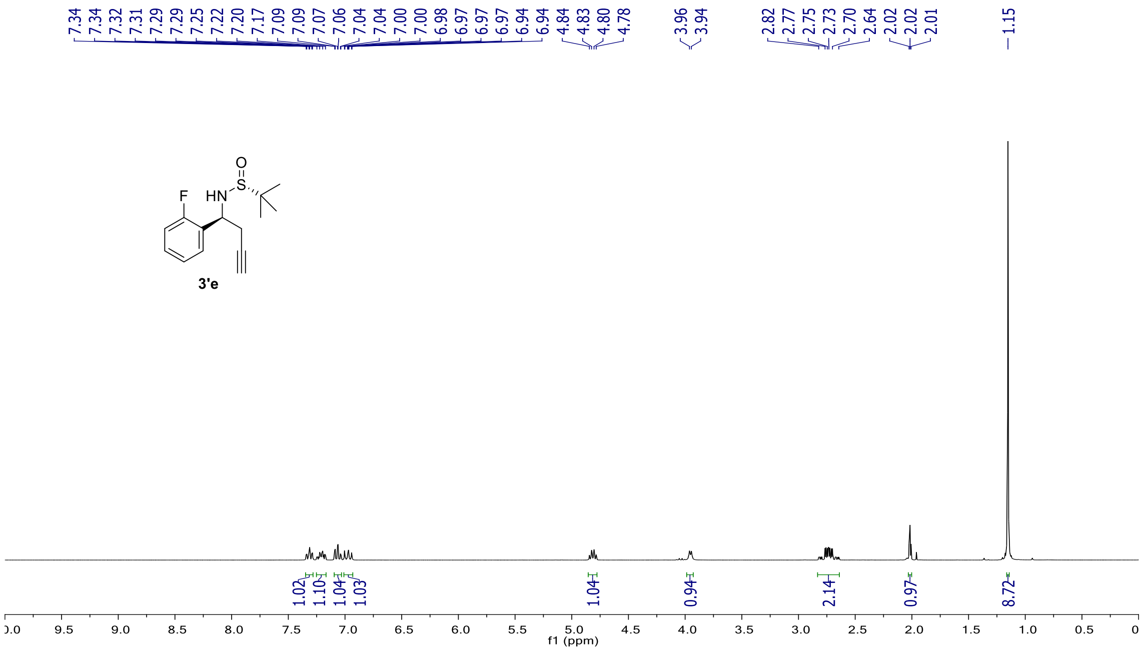

^{19}F NMR spectrum of compound **3’e** (282 MHz, $CDCl_3$)

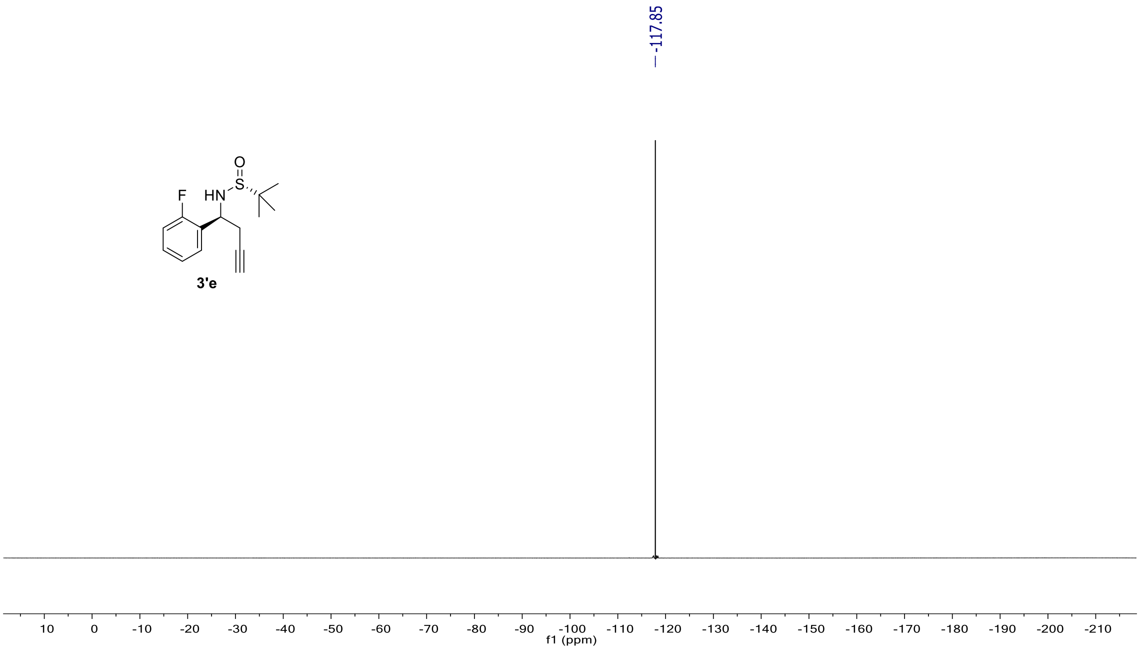

^{13}C NMR spectrum of compound **3’e** (75 MHz, $CDCl_3$)

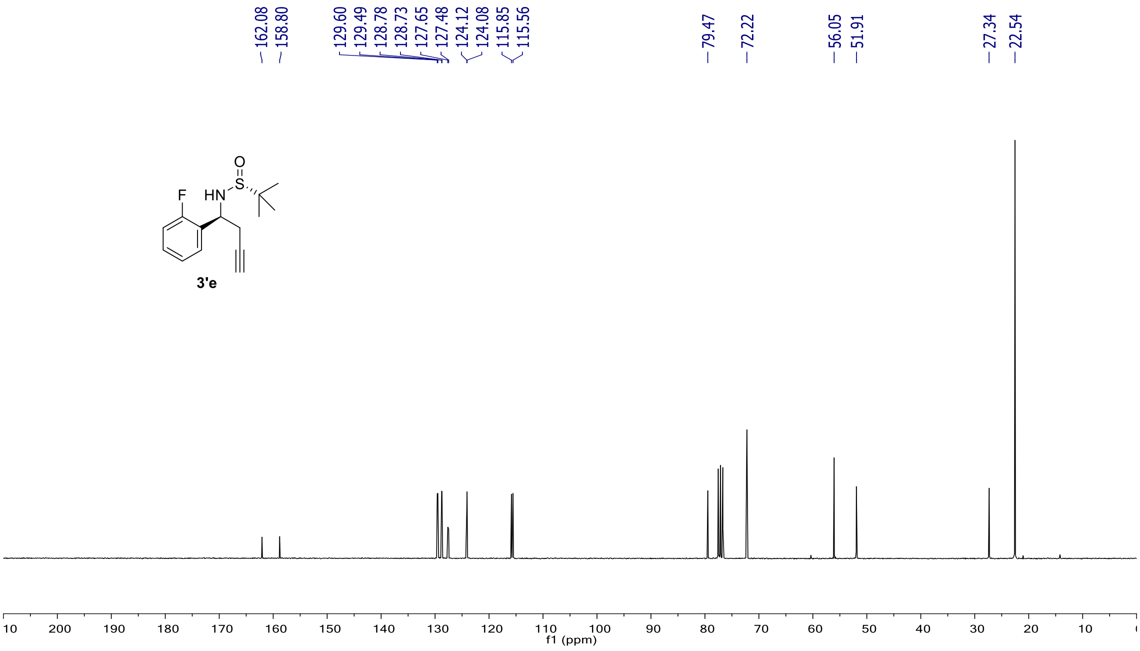

^{1}H NMR spectrum of compound **4ab** (300 MHz, $CDCl_3$)

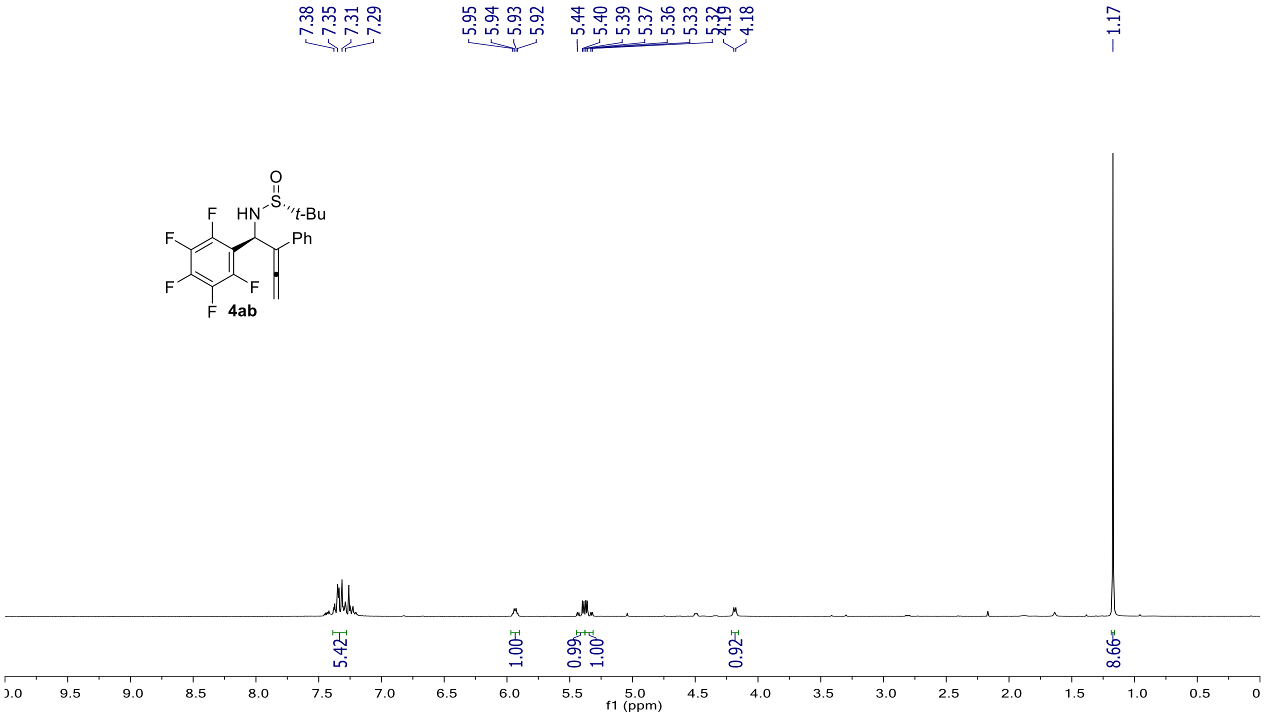

^{1}F NMR spectrum of compound **4ab** (282 MHz, $CDCl_3$)

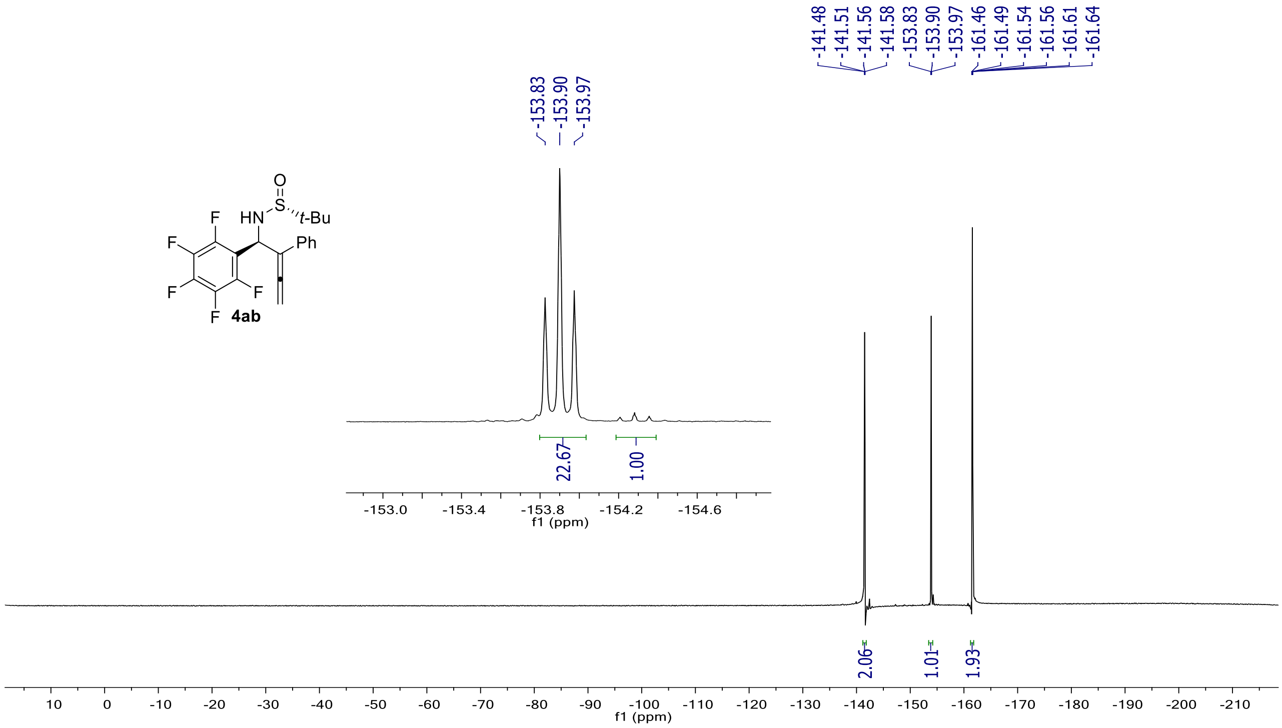

^{13}C NMR spectrum of compound **4ab** (75 MHz, $CDCl_3$)

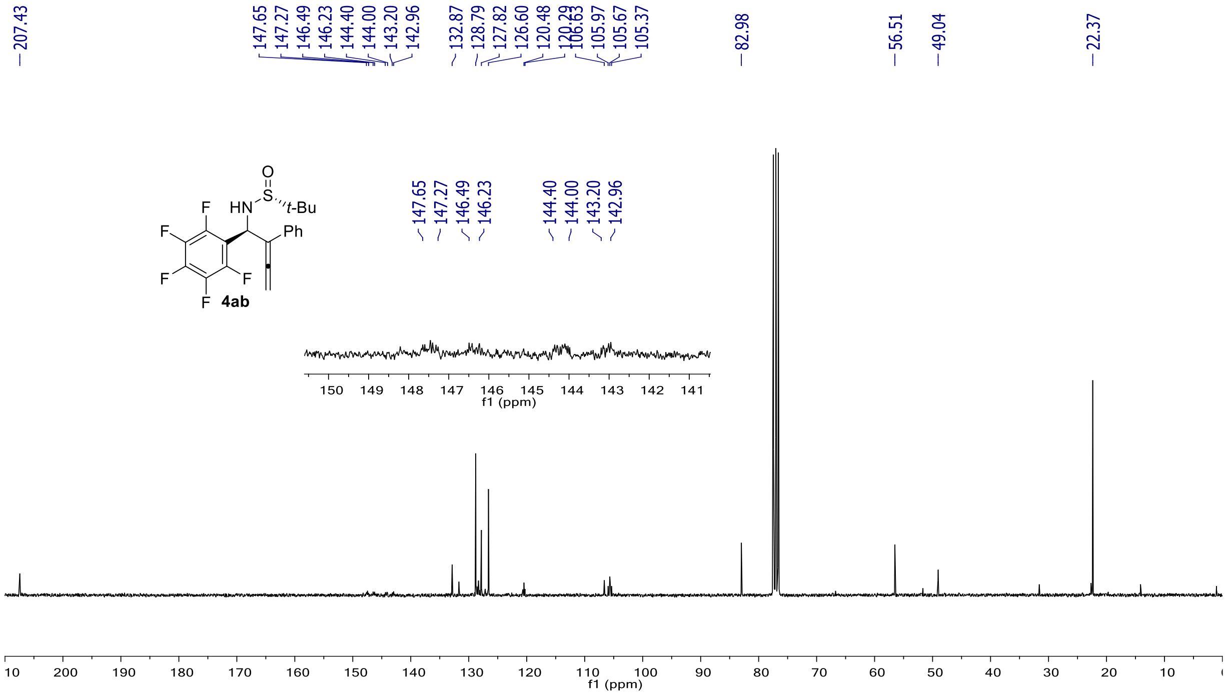

^{1}H NMR spectrum of compound **4ac** (300 MHz, $CDCl_3$)

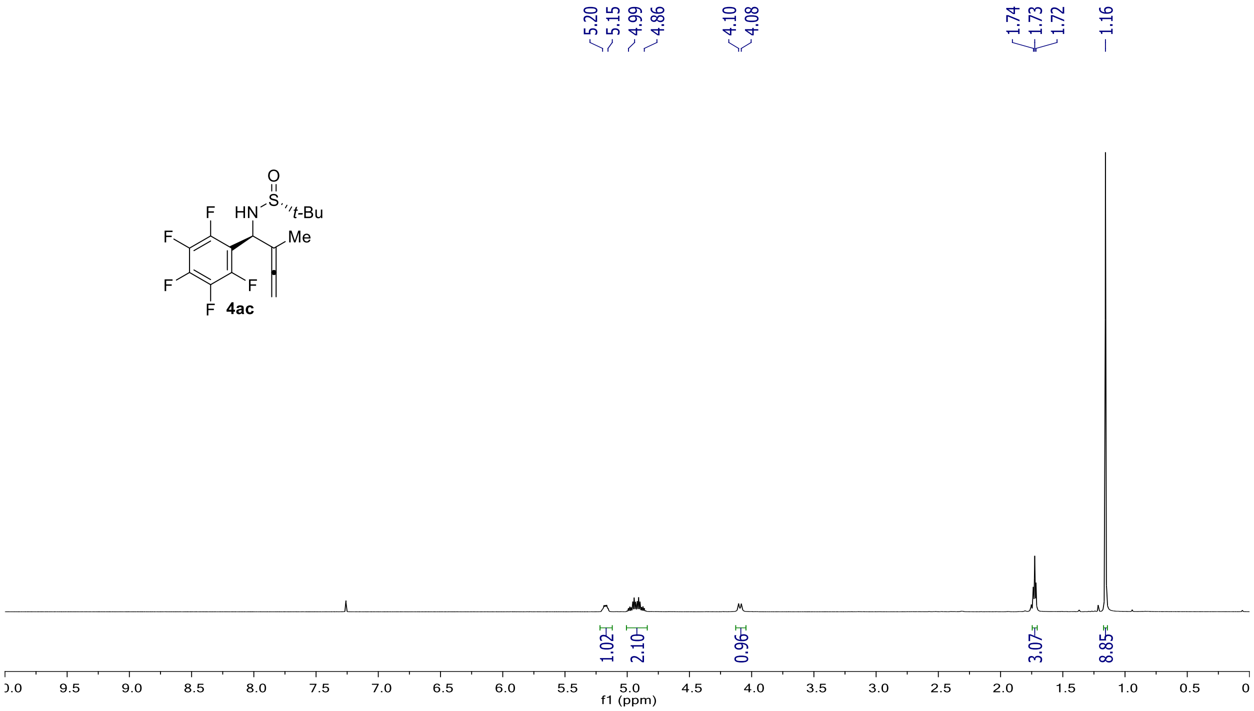

^{19}F NMR spectrum of compound **4ac** (282 MHz, $CDCl_3$)

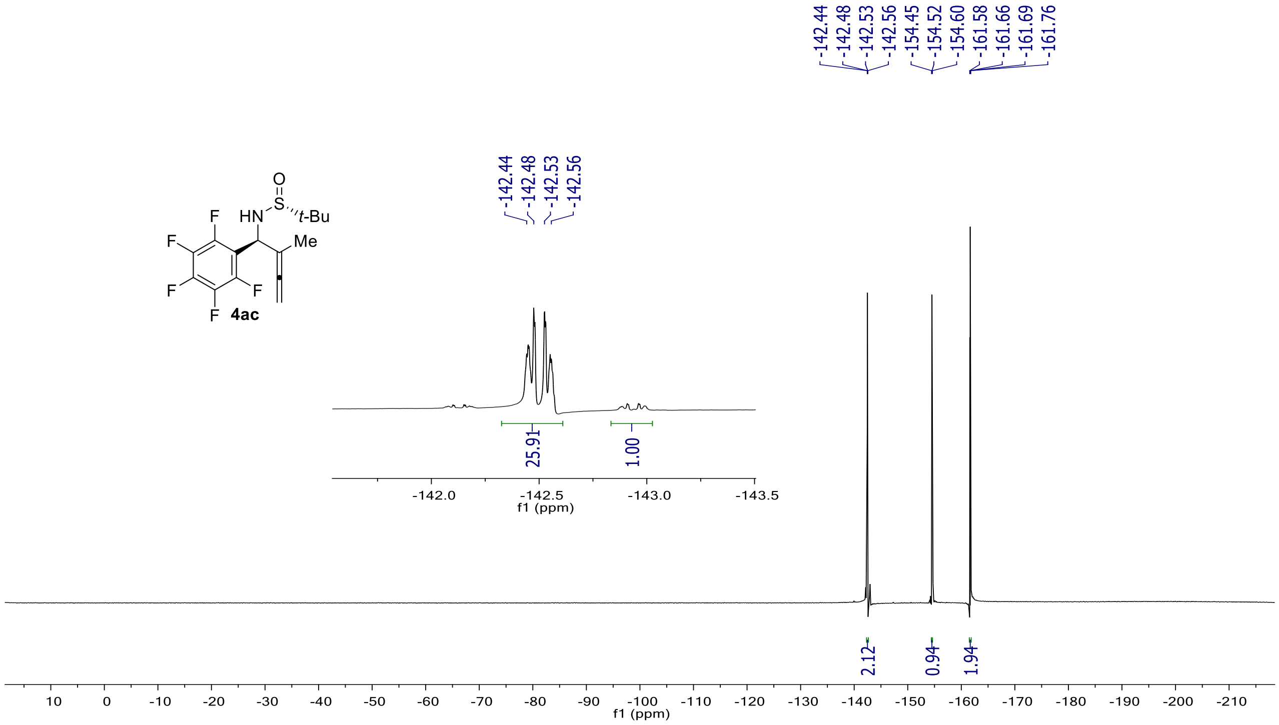

^{13}C NMR spectrum of compound **4ab** (75 MHz, $CDCl_3$)

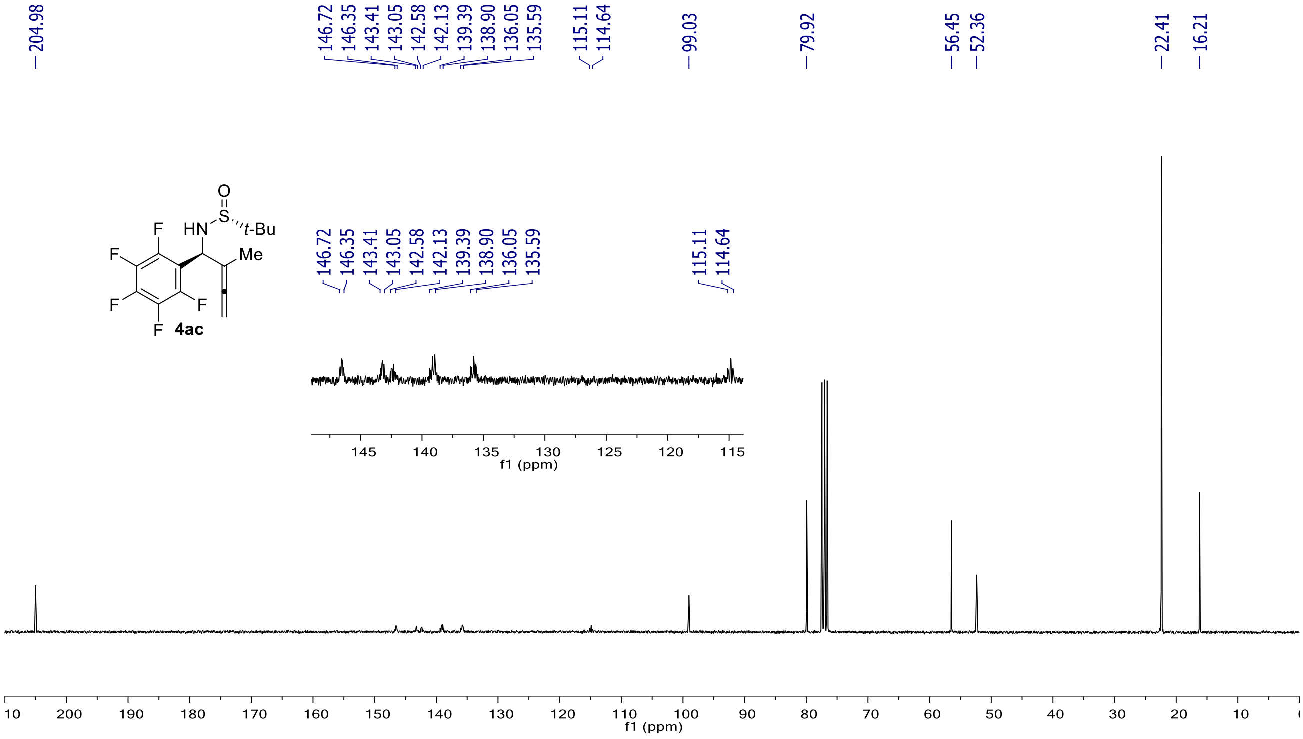

^1H NMR spectrum of compound **4bb** (300 MHz, $CDCl_3$)

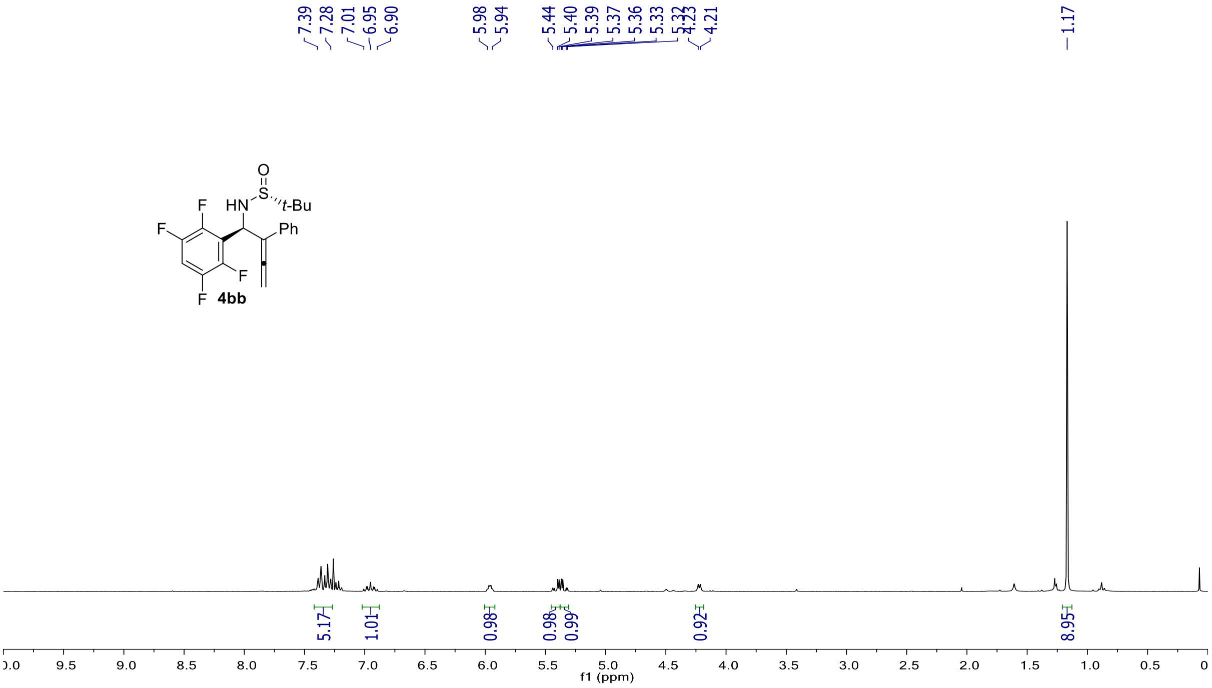

^{19}F NMR spectrum of compound **4bb** (282 MHz, $CDCl_3$)

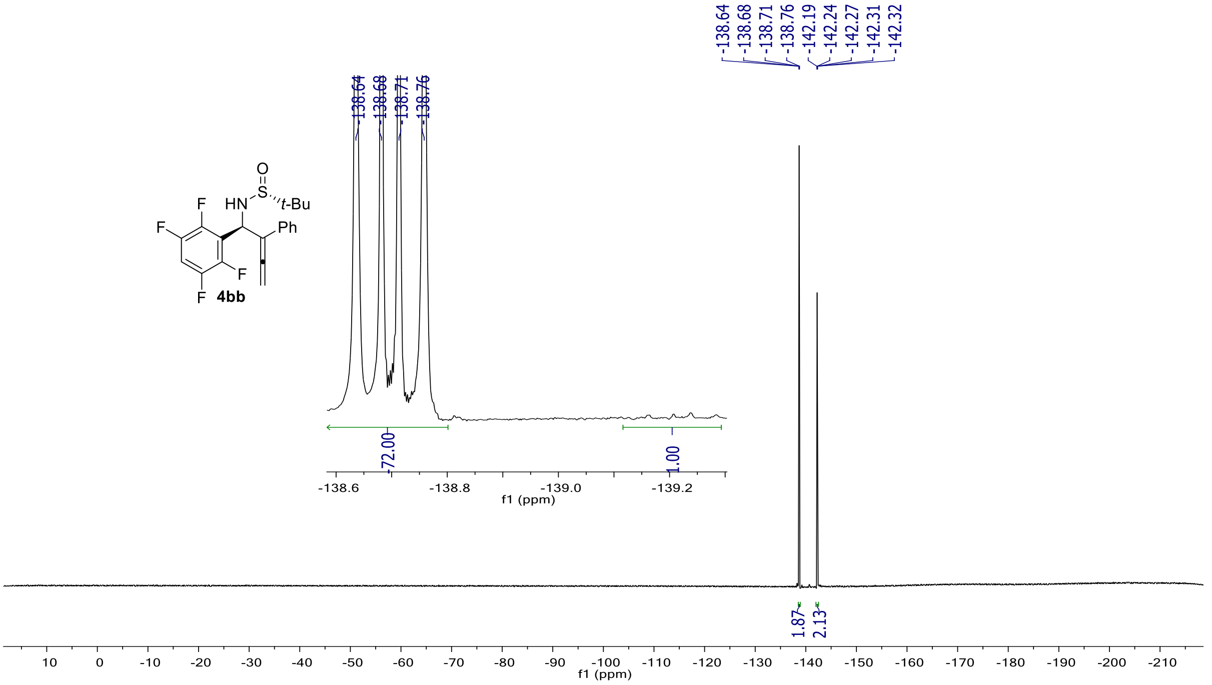

^{13}C NMR spectrum of compound **4bb** (75 MHz, $CDCl_3$)

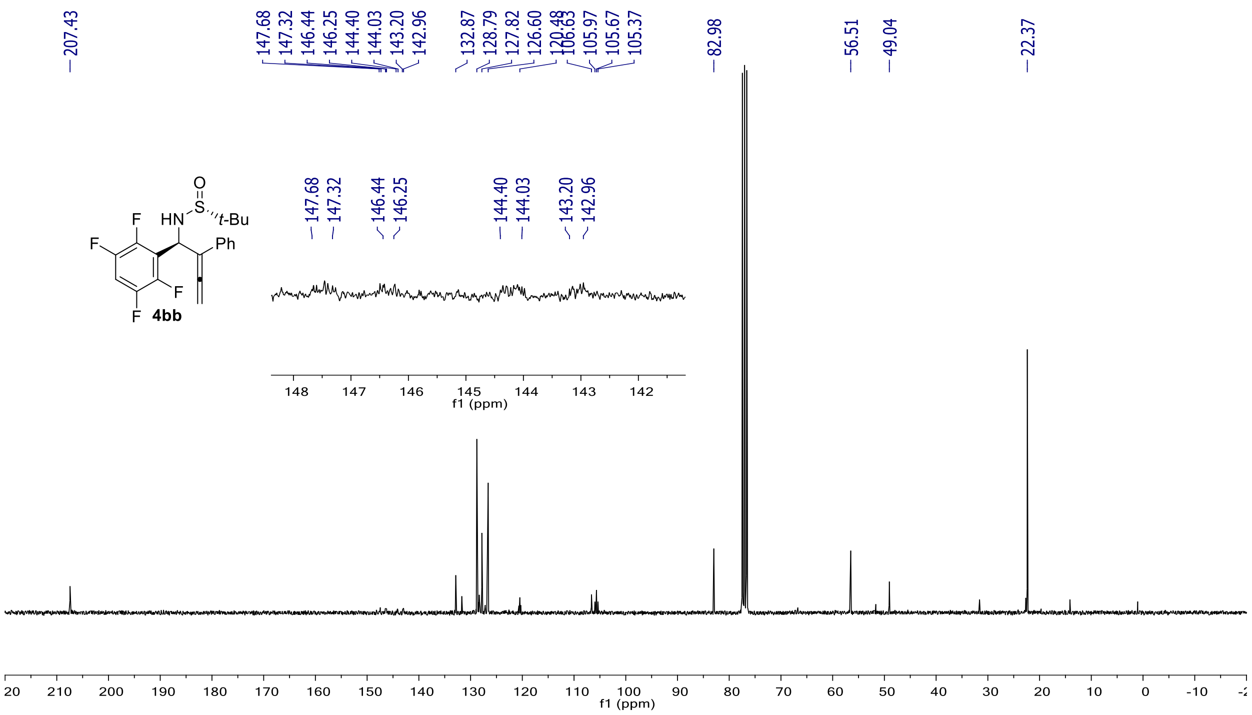

^{1}H NMR spectrum of compound **4bc** (300 MHz, $CDCl_3$)

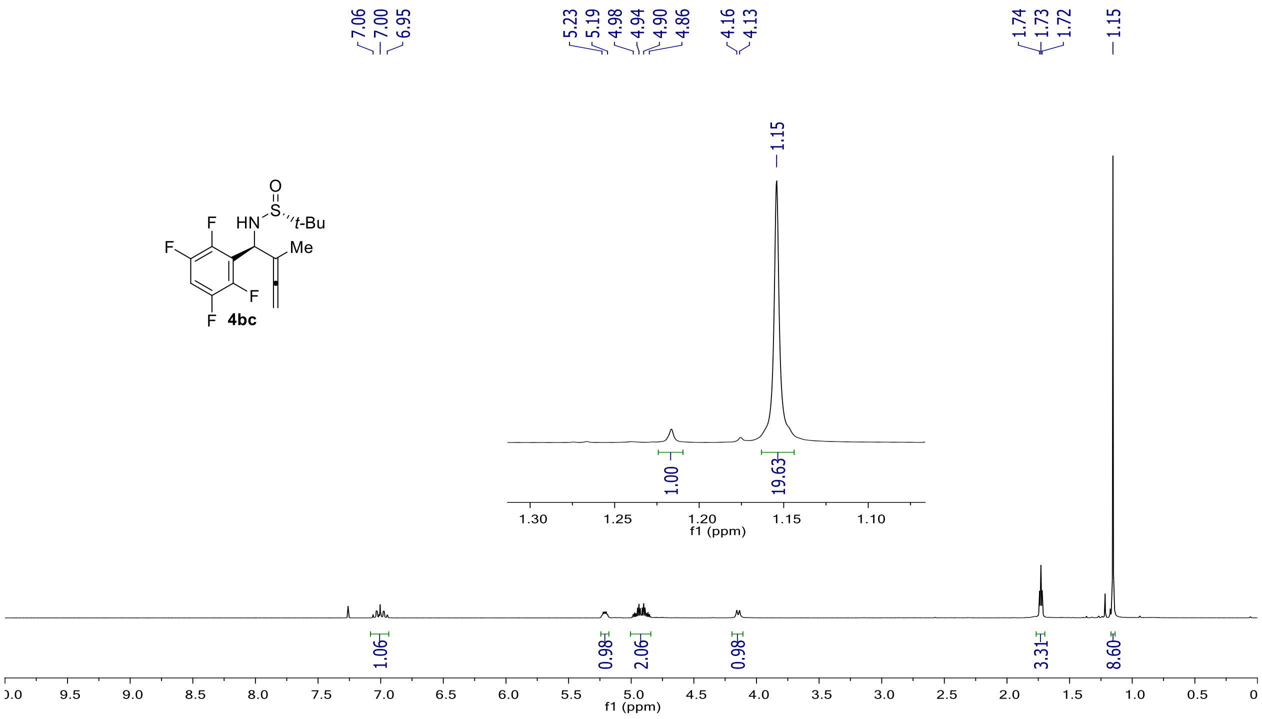

^{19}F NMR spectrum of compound **4bc** (282 MHz, $CDCl_3$)

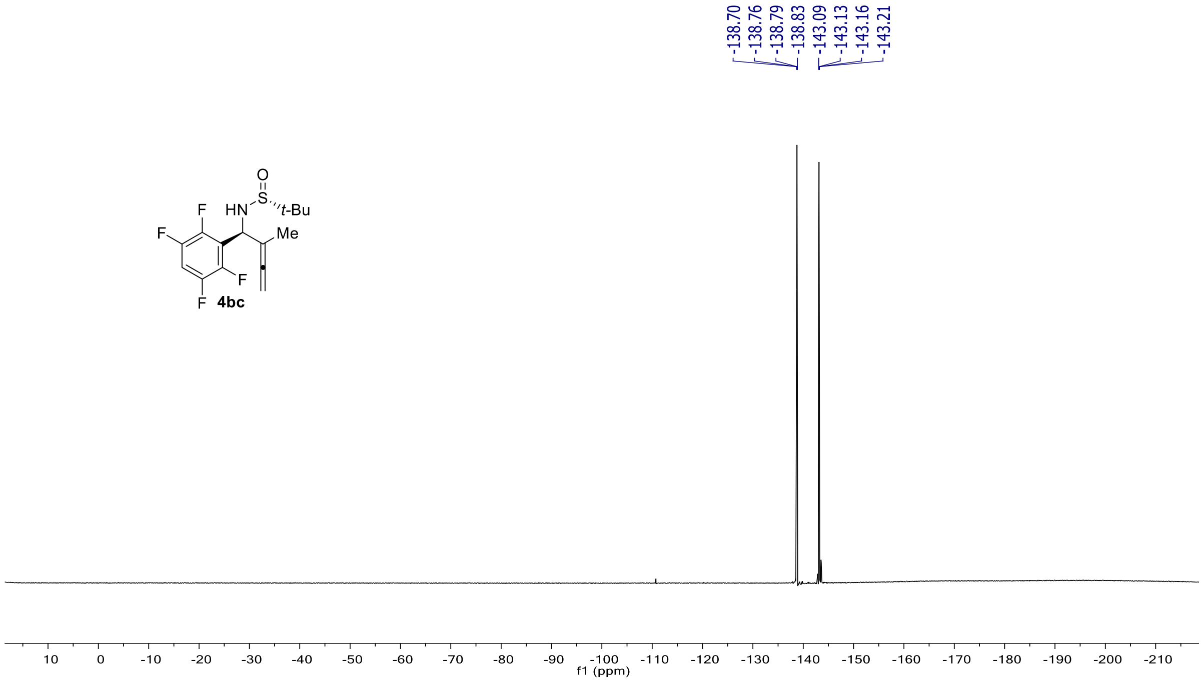

^{13}C NMR spectrum of compound **4bc** (75 MHz, $CDCl_3$)

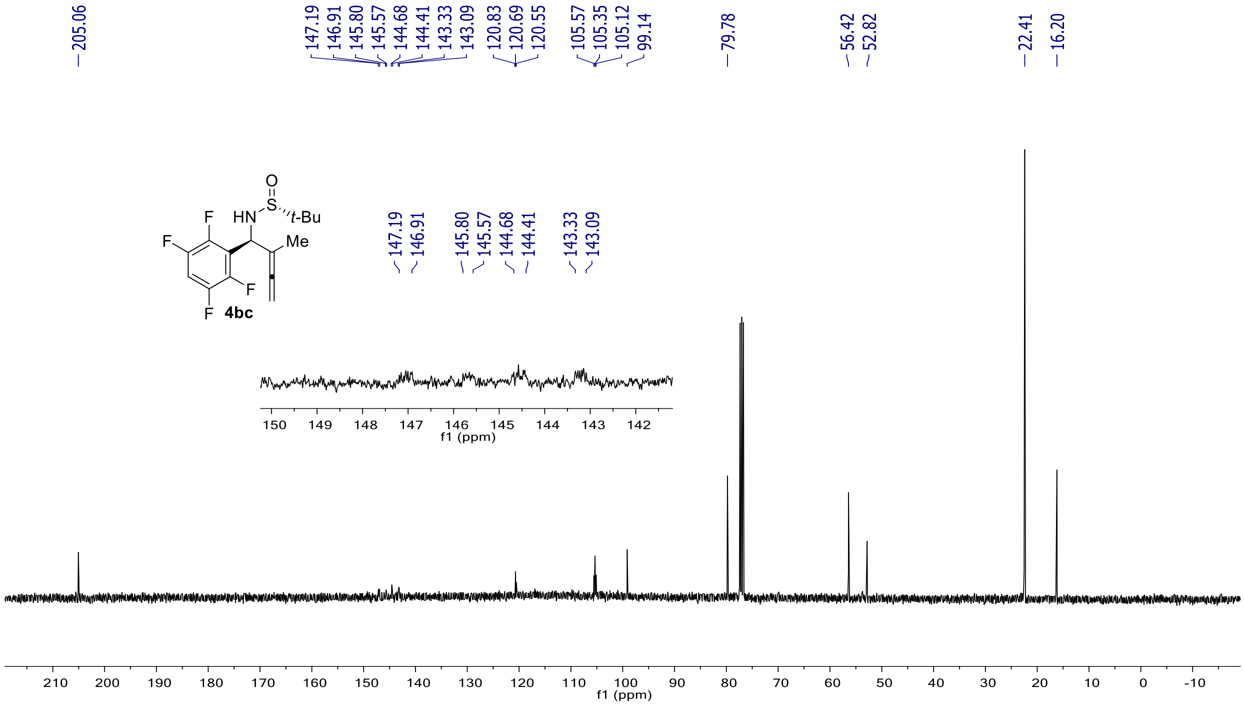

^{1}H NMR spectrum of compound **4cb** (300 MHz, $CDCl_3$)

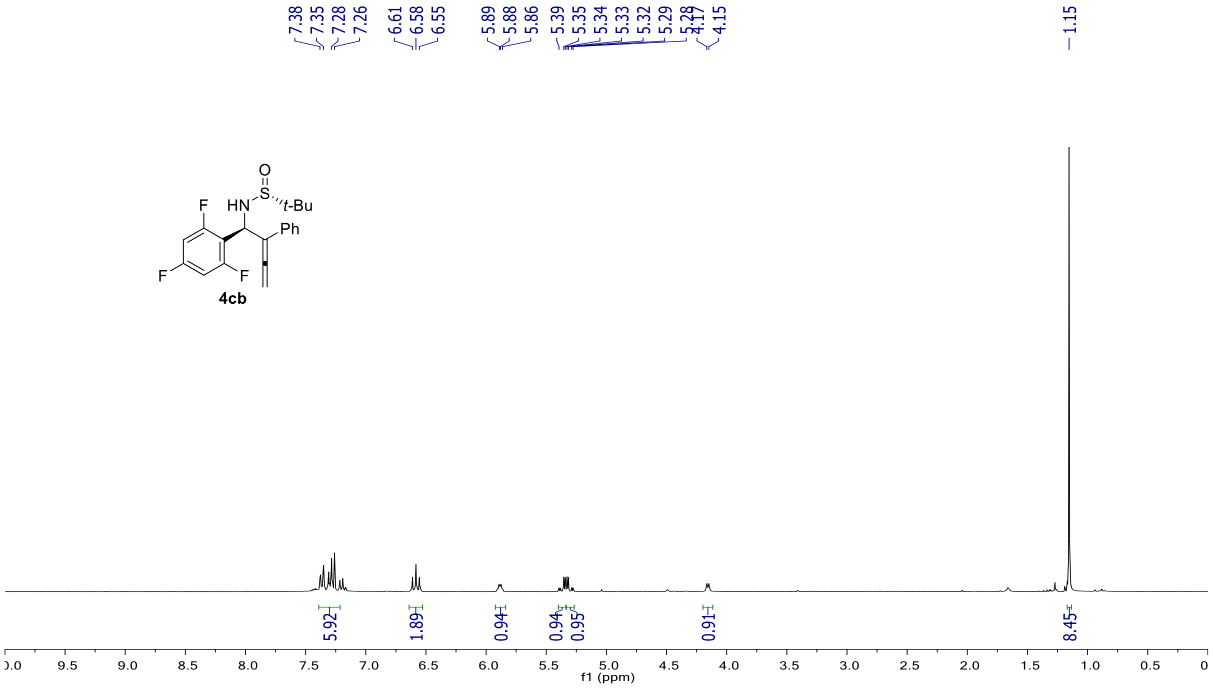

^{19}F NMR spectrum of compound **4cb** (282 MHz, $CDCl_3$)

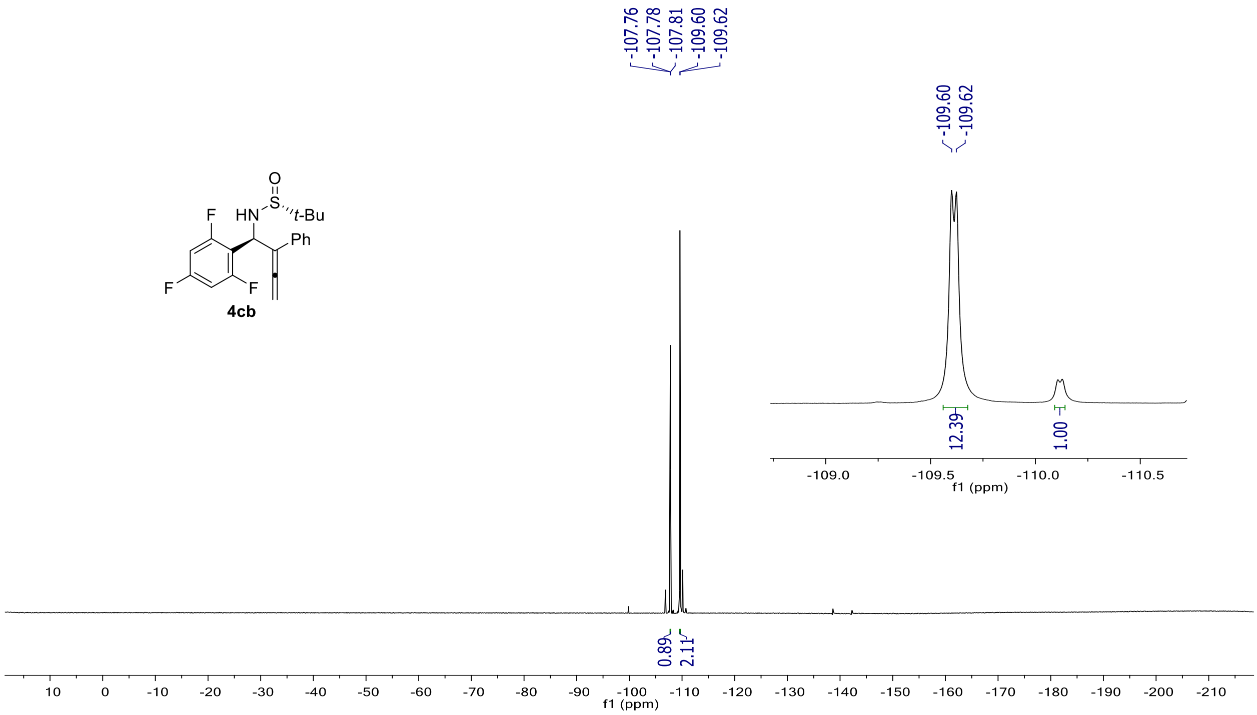

^{13}C NMR spectrum of compound **4cb** (75 MHz, $CDCl_3$)

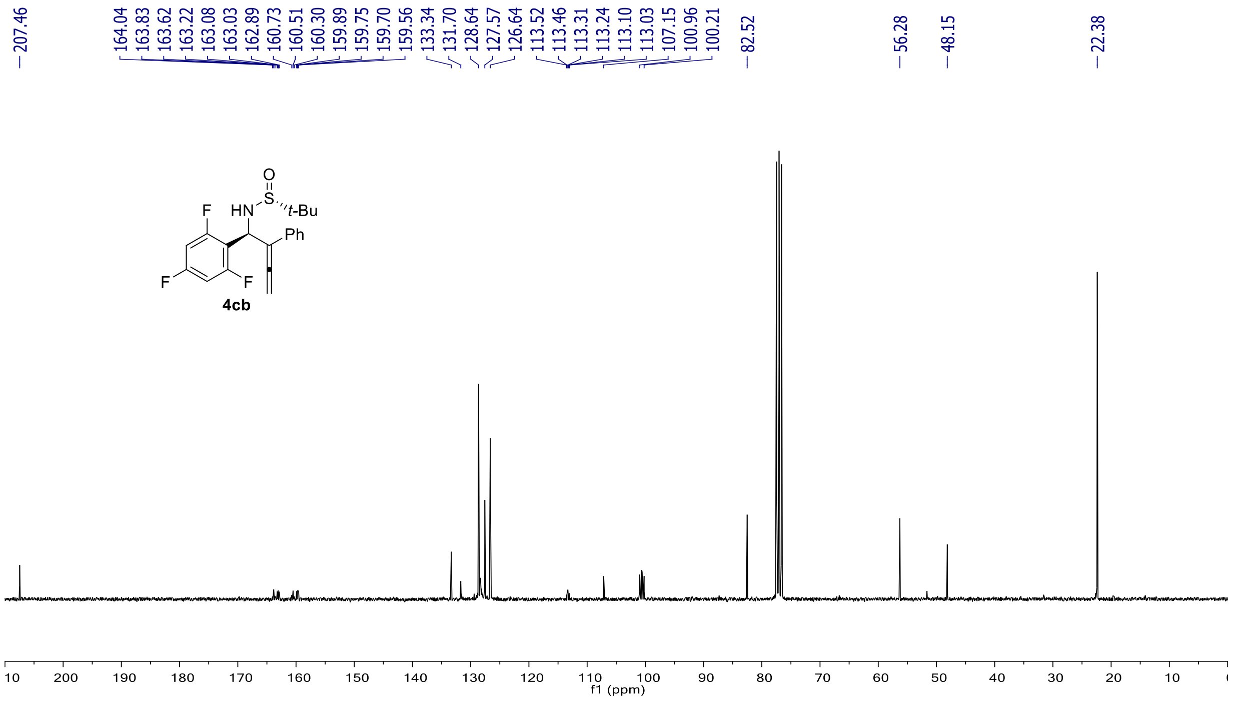

^{1}H NMR spectrum of compound **4cc** (300 MHz, $CDCl_3$)

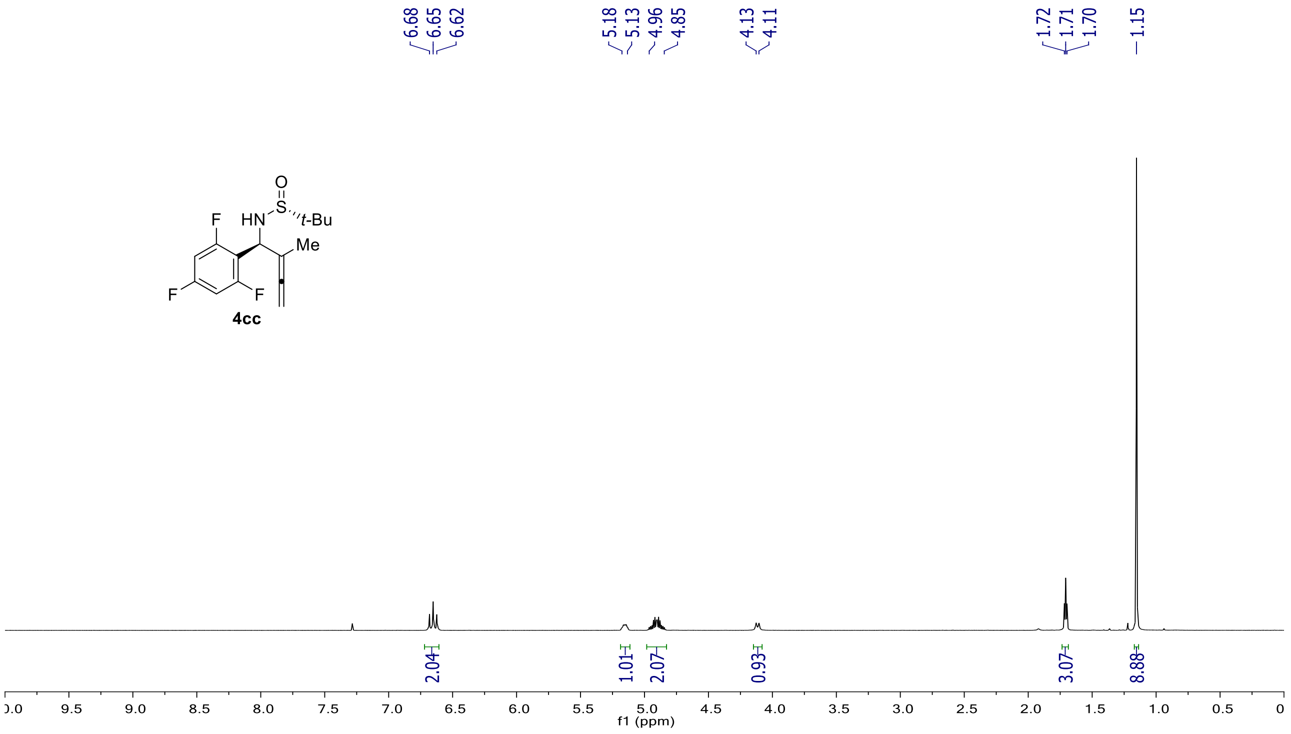

^{19}F NMR spectrum of compound **4cc** (282 MHz, $CDCl_3$)

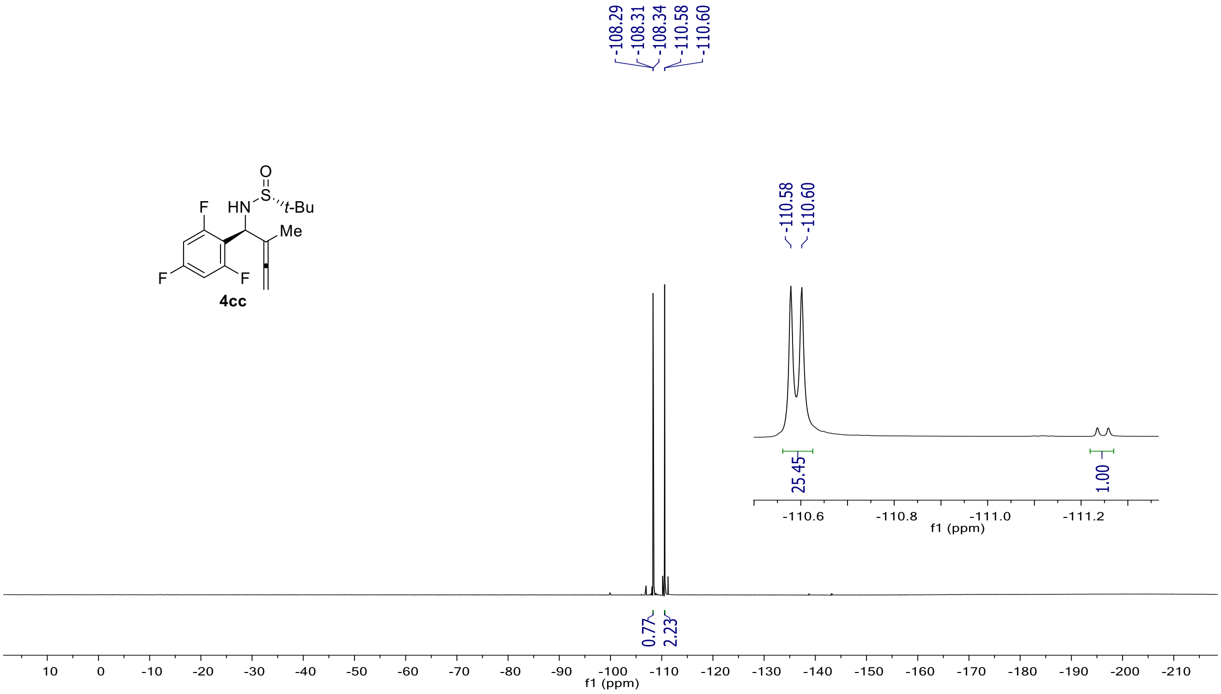

^{13}C NMR spectrum of compound **4cc** (75 MHz, $CDCl_3$)

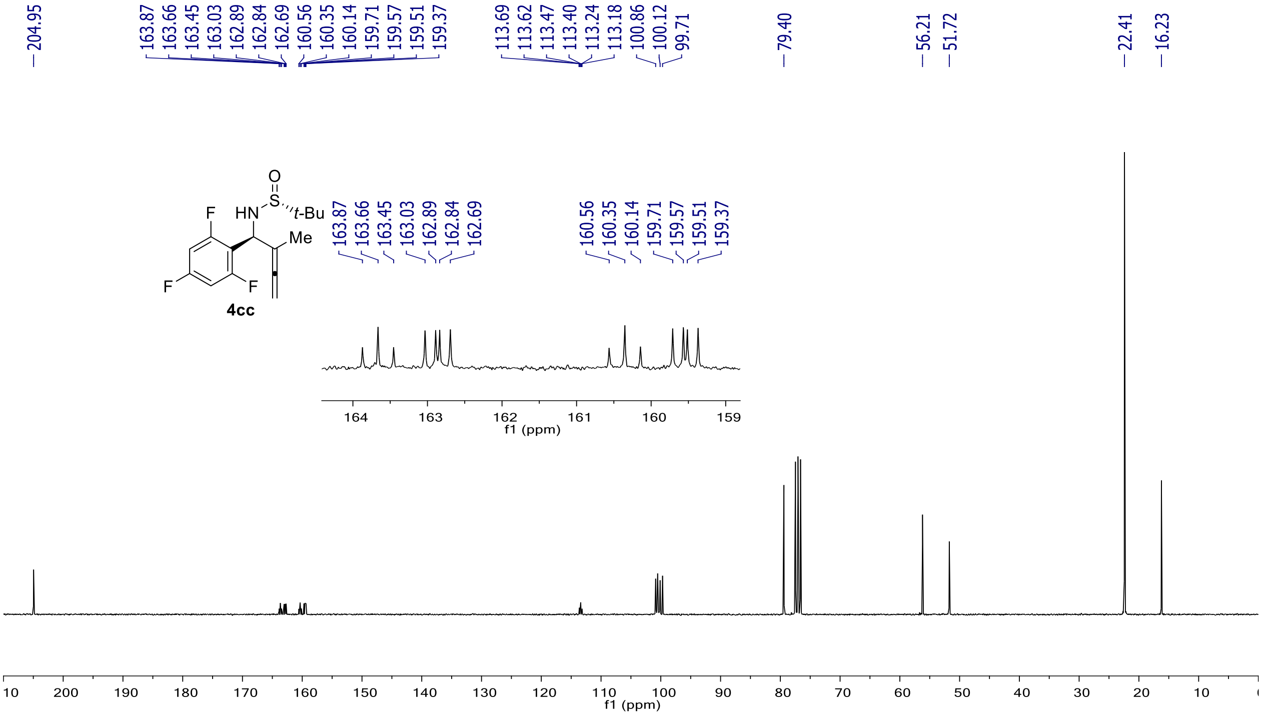

^{1}H NMR spectrum of compound **4db** (300 MHz, $CDCl_3$)

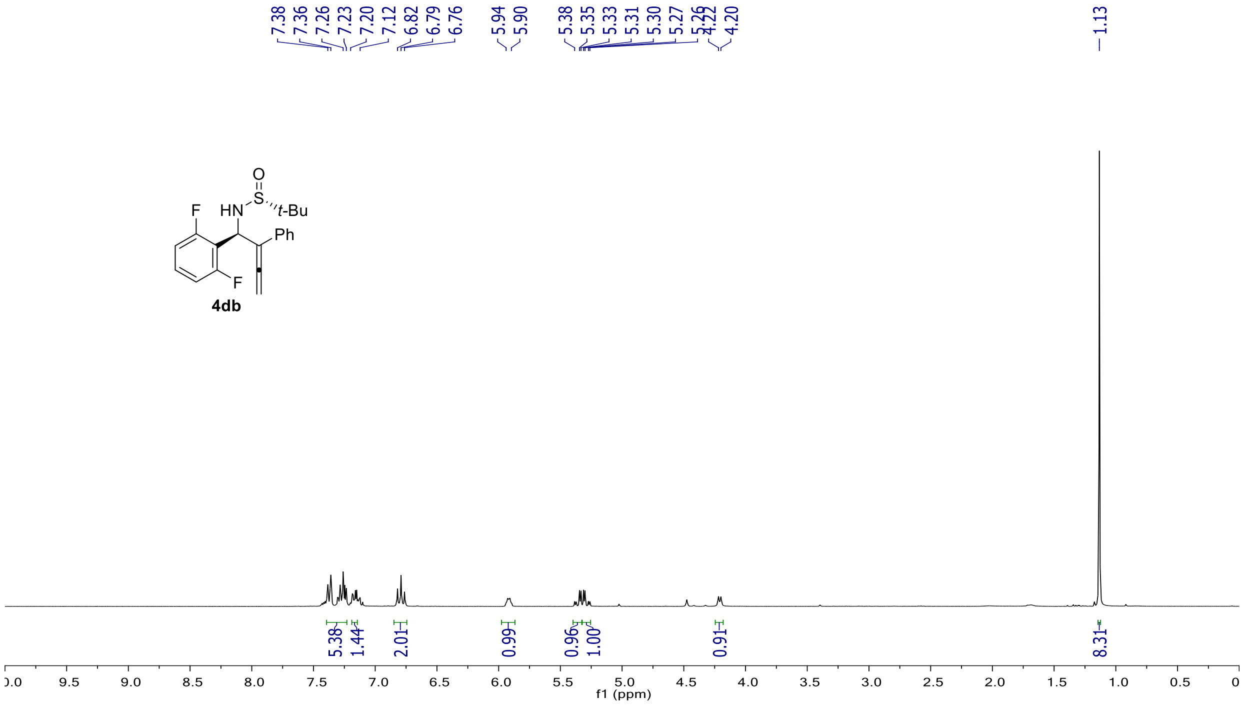

^{19}F NMR spectrum of compound **4db** (282 MHz, $CDCl_3$)

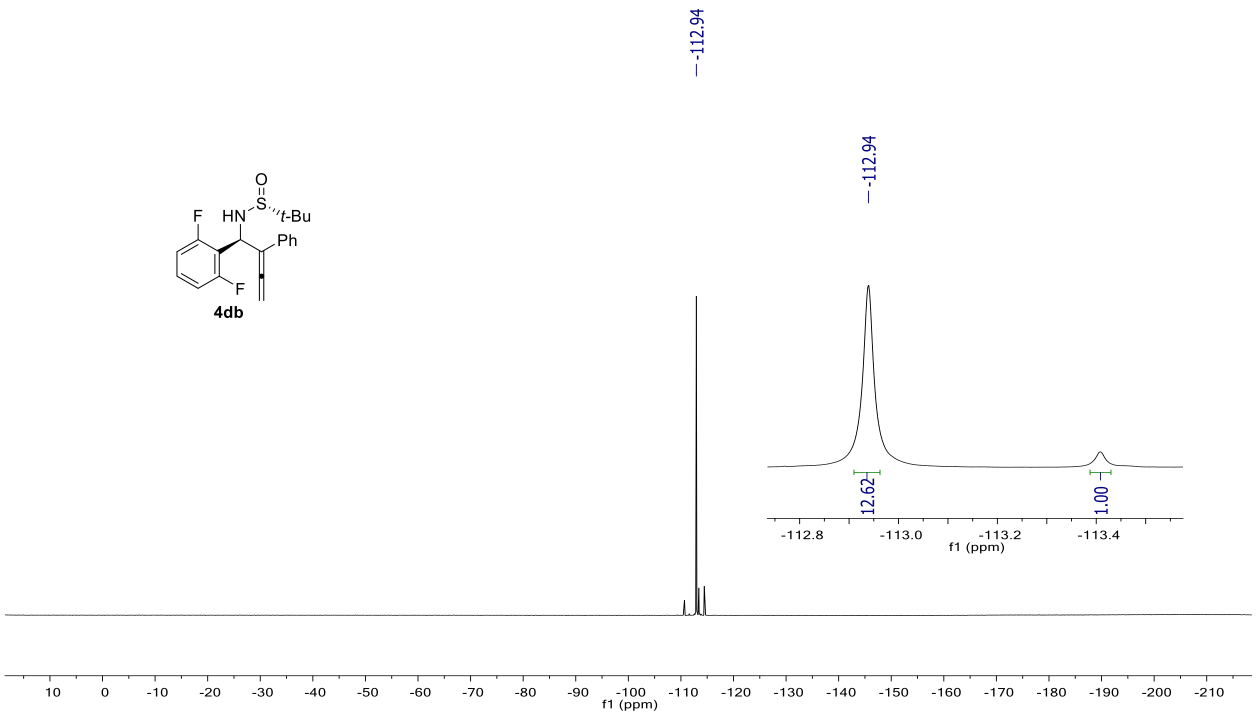

^{13}C NMR spectrum of compound **4db** (75 MHz, $CDCl_3$)

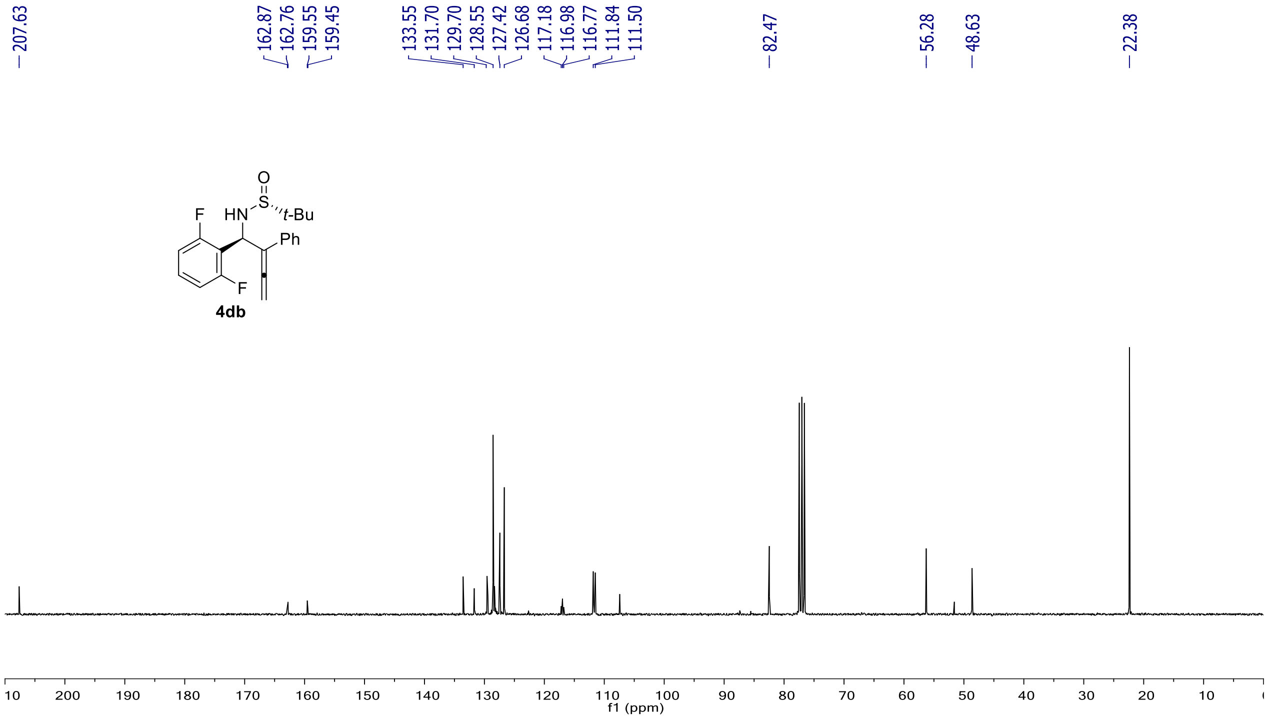

^{1}H NMR spectrum of compound **4dc** (300 MHz, $CDCl_3$)

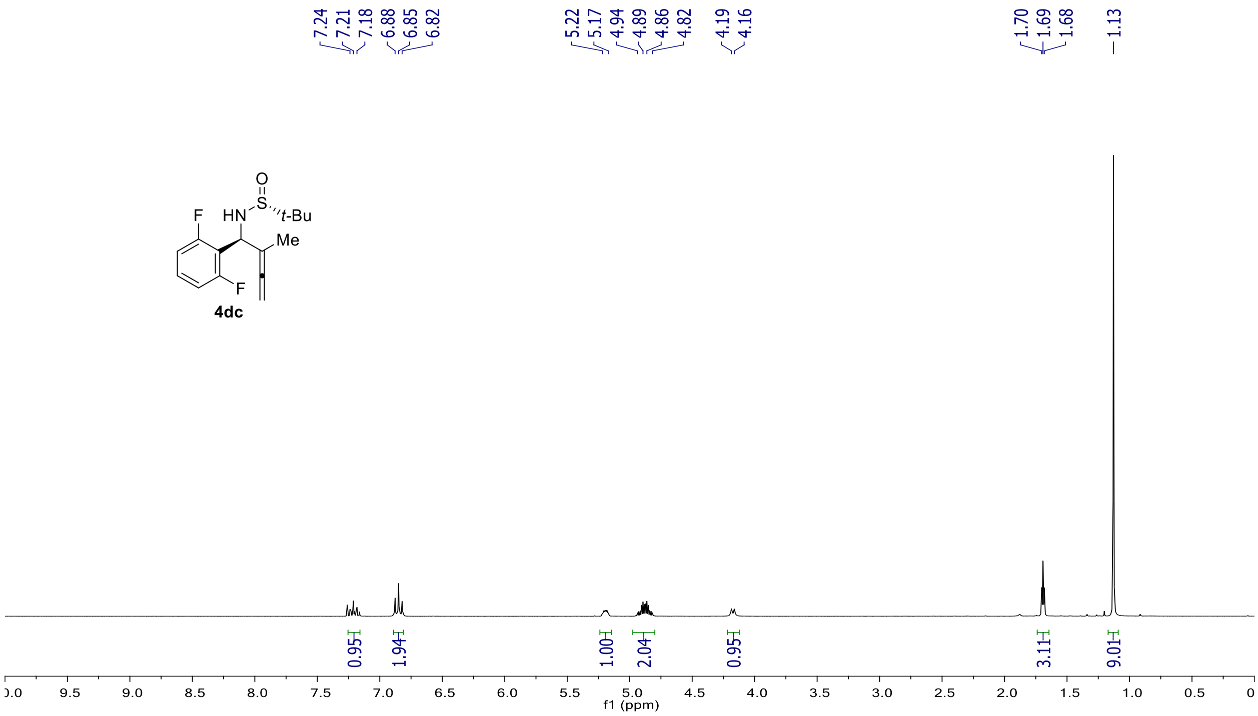

^{19}F NMR spectrum of compound **4dc** (282 MHz, $CDCl_3$)

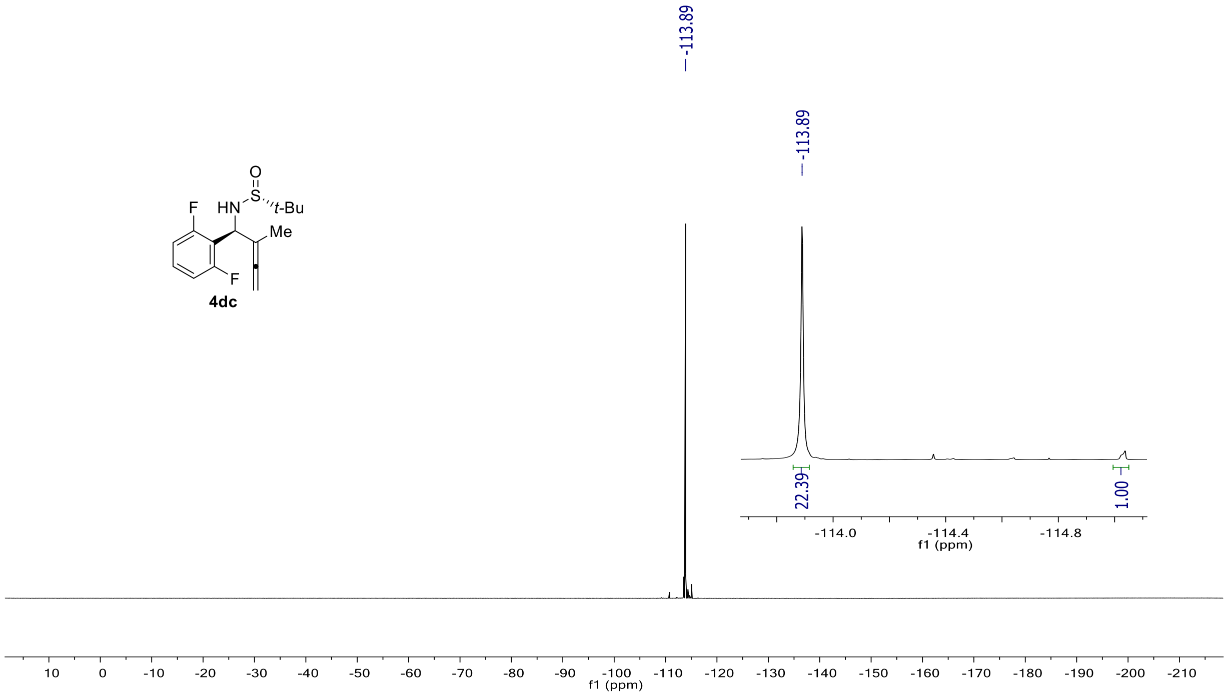

^{13}C NMR spectrum of compound **4dc** (75 MHz, $CDCl_3$)

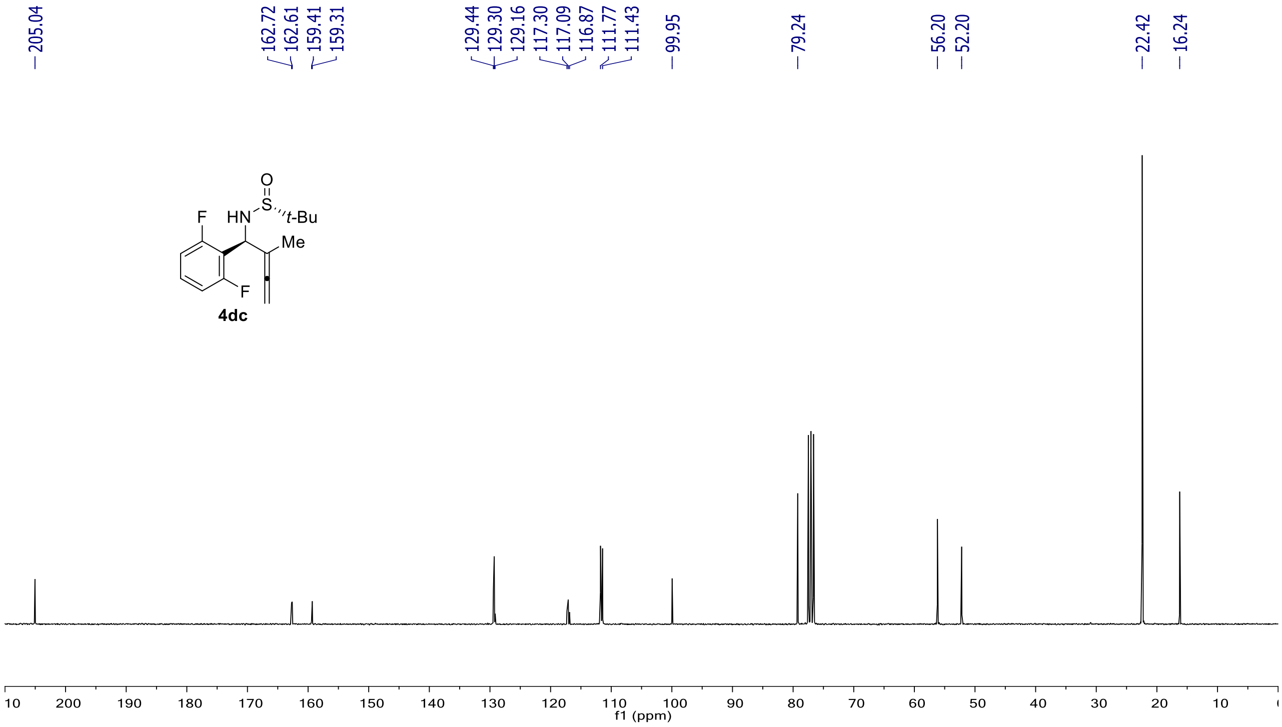

^{1}H NMR spectrum of compound **4eb** (300 MHz, $CDCl_3$)

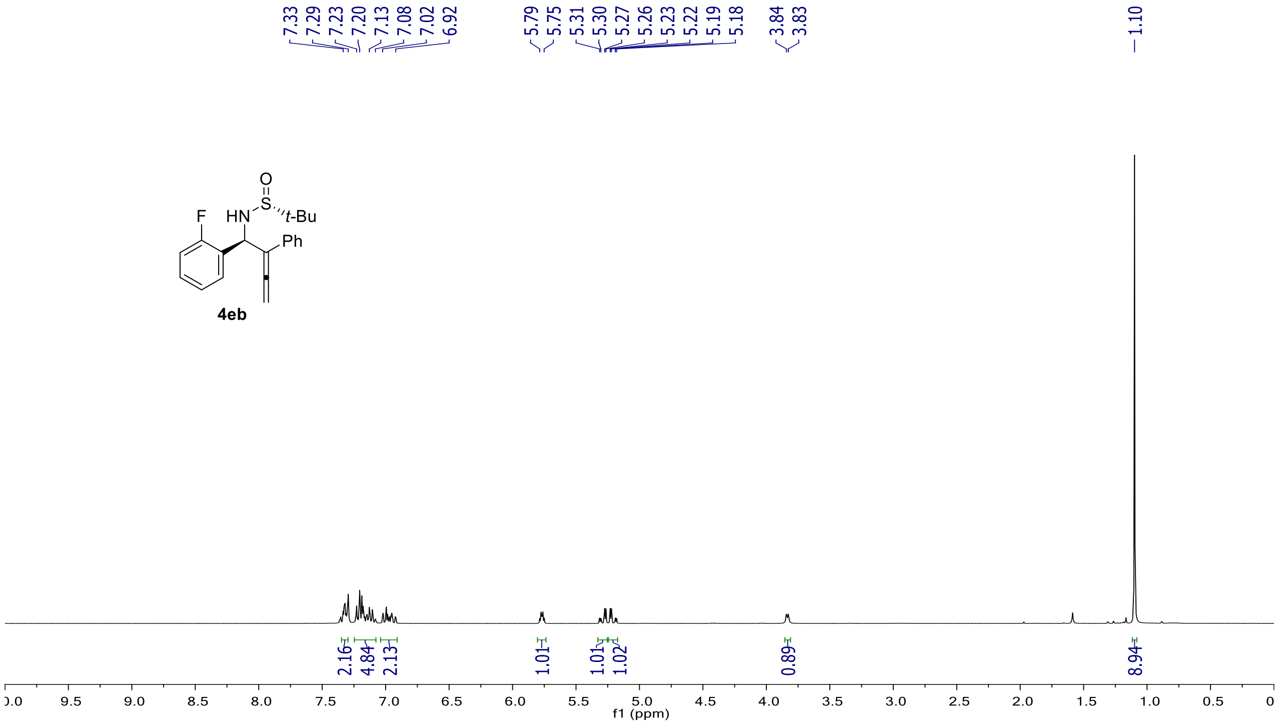

^{19}F NMR spectrum of compound **4eb** (282 MHz, $CDCl_3$)

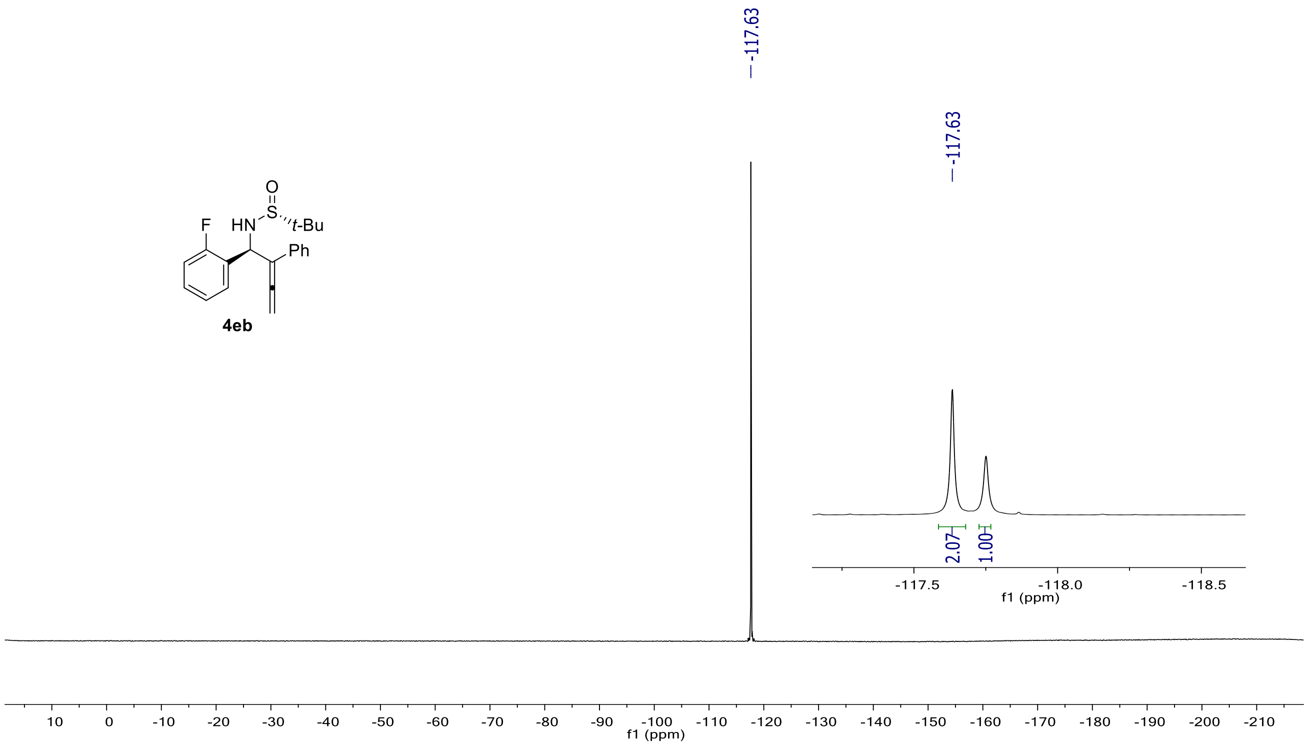

^{13}C NMR spectrum of compound **4eb** (75 MHz, $CDCl_3$)

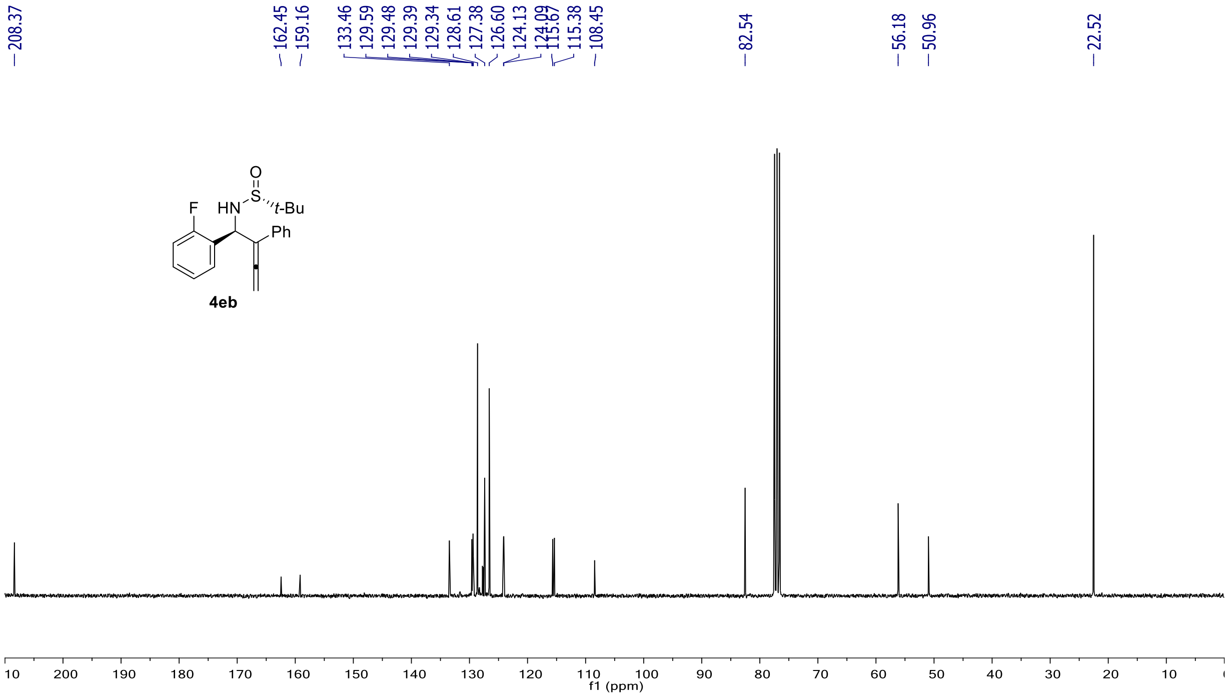

^{1}H NMR spectrum of compound **4ec** (300 MHz, $CDCl_3$)

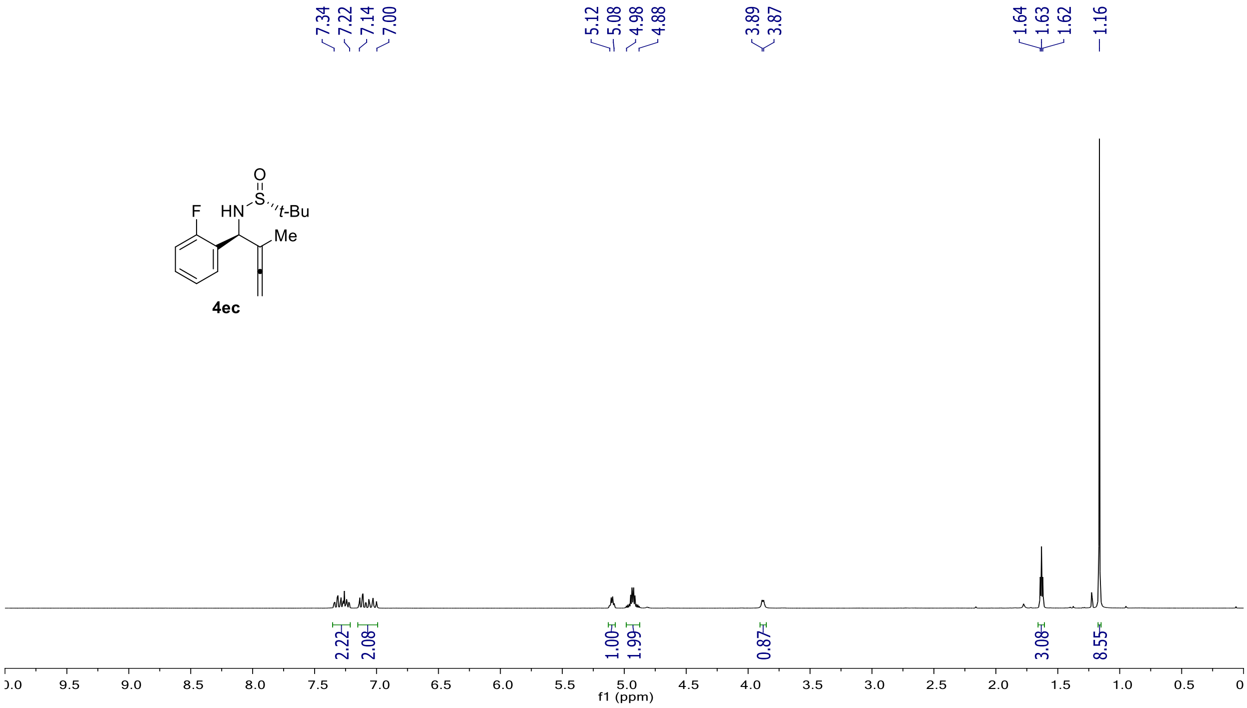

^{19}F NMR spectrum of compound **4ec** (282 MHz, $CDCl_3$)

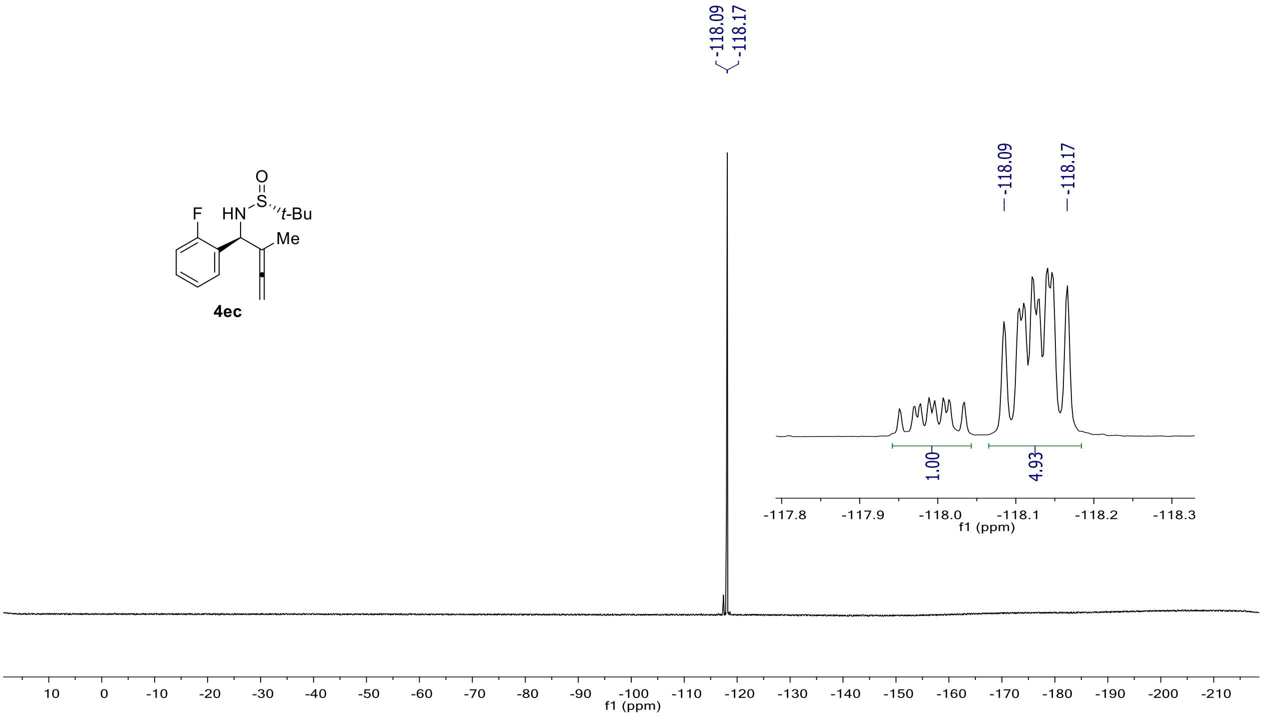

^{13}C NMR spectrum of compound **4ec** (75 MHz, $CDCl_3$)

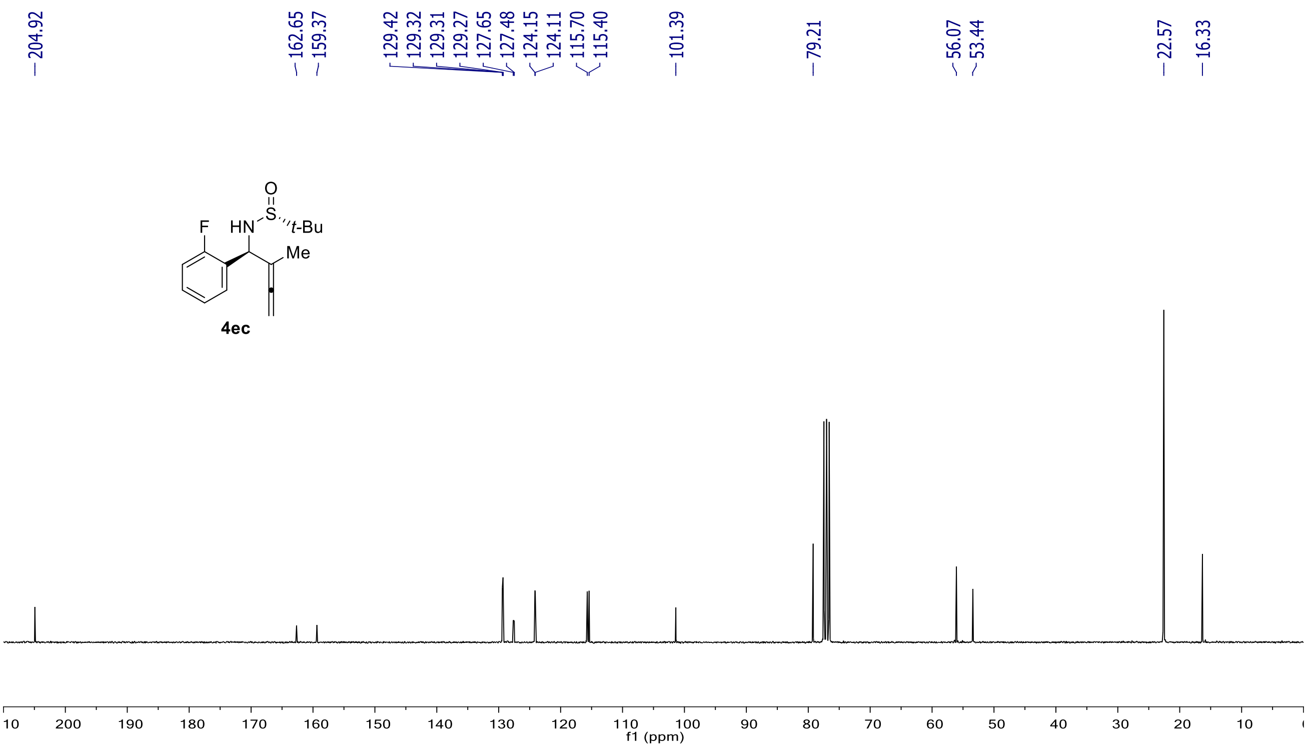

^{1}H NMR spectrum of compound **4fb** (300 MHz, $CDCl_3$)

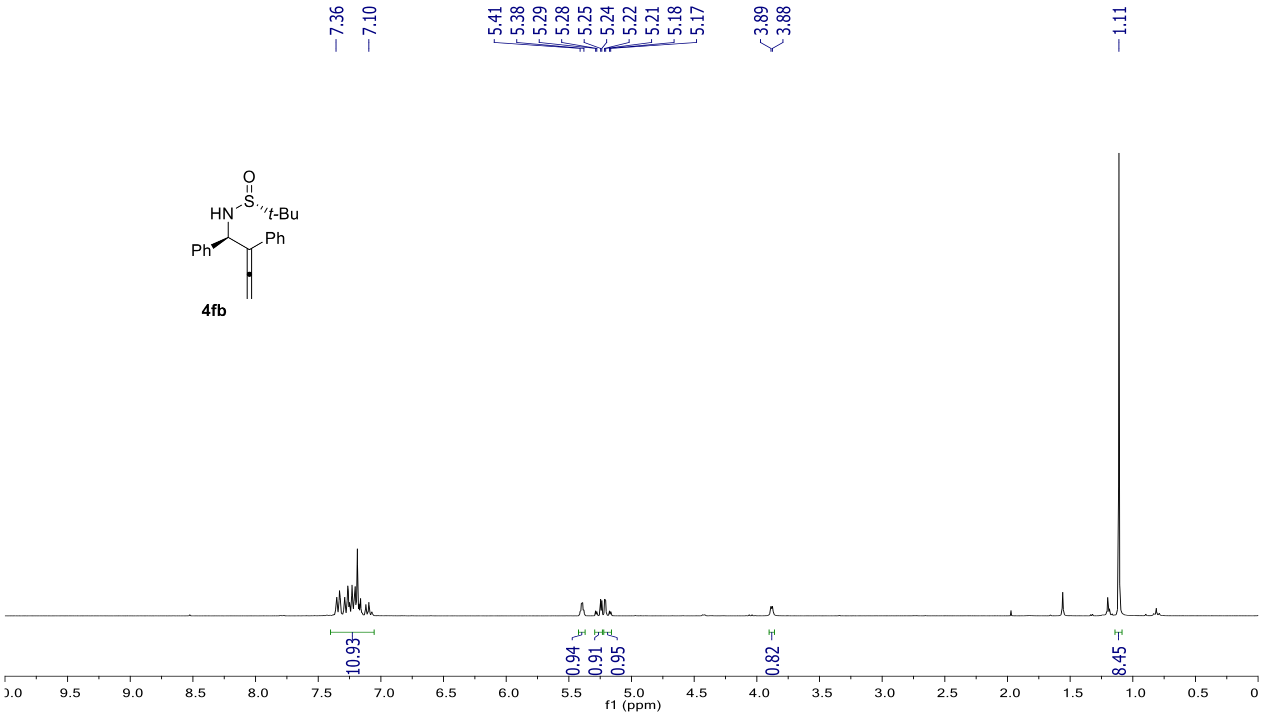

^{19}F NMR spectrum of compound **4fb** (282 MHz, $CDCl_3$)

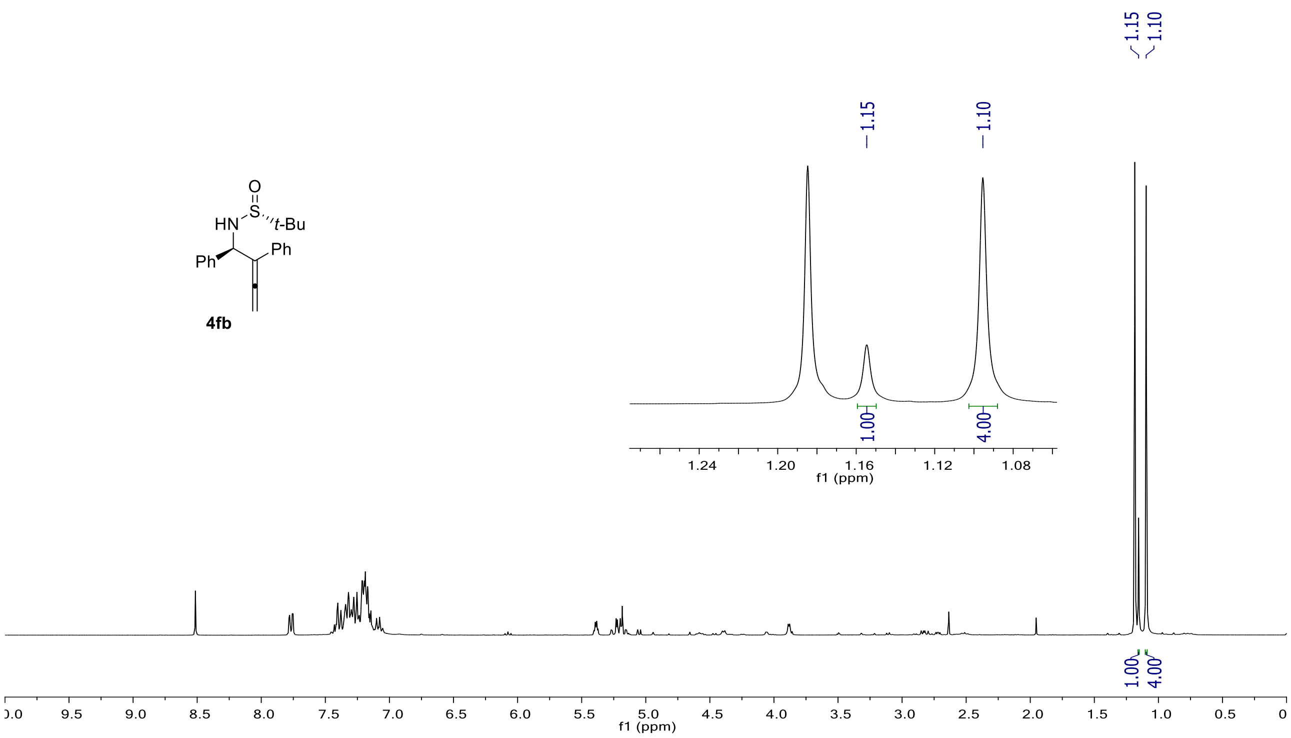

^{13}C NMR spectrum of compound **4fb** (75 MHz, $CDCl_3$)

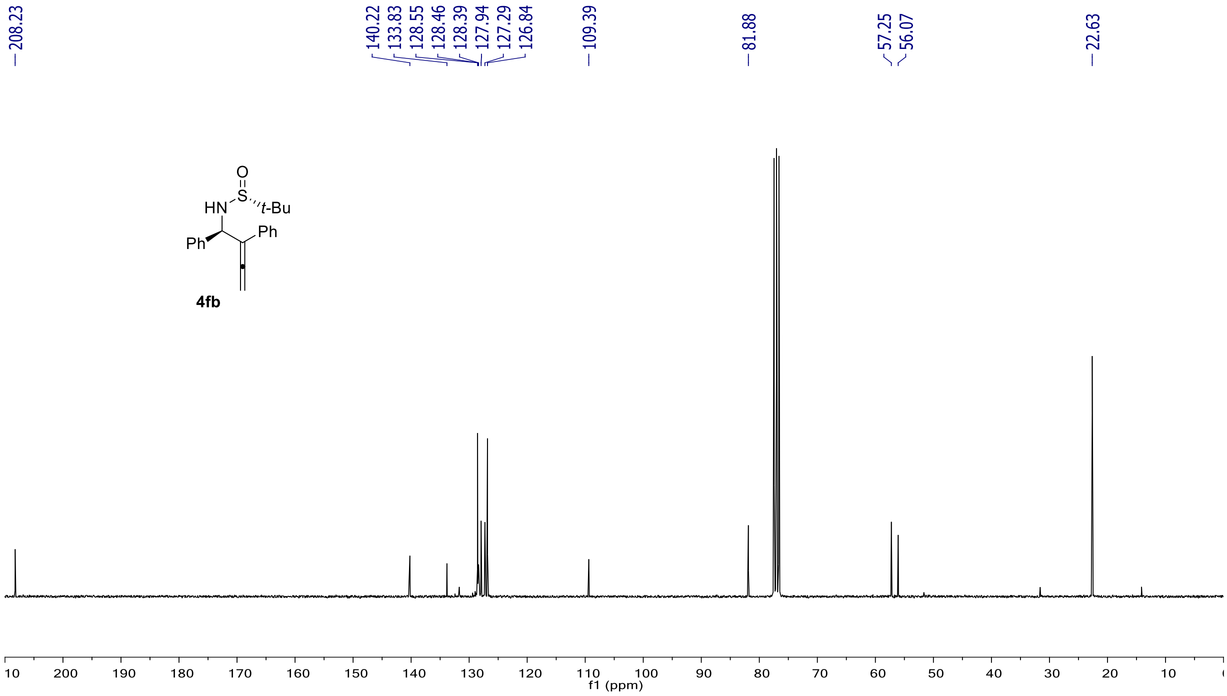

^1H NMR spectrum of compound **4fc** (300 MHz, $CDCl_3$)

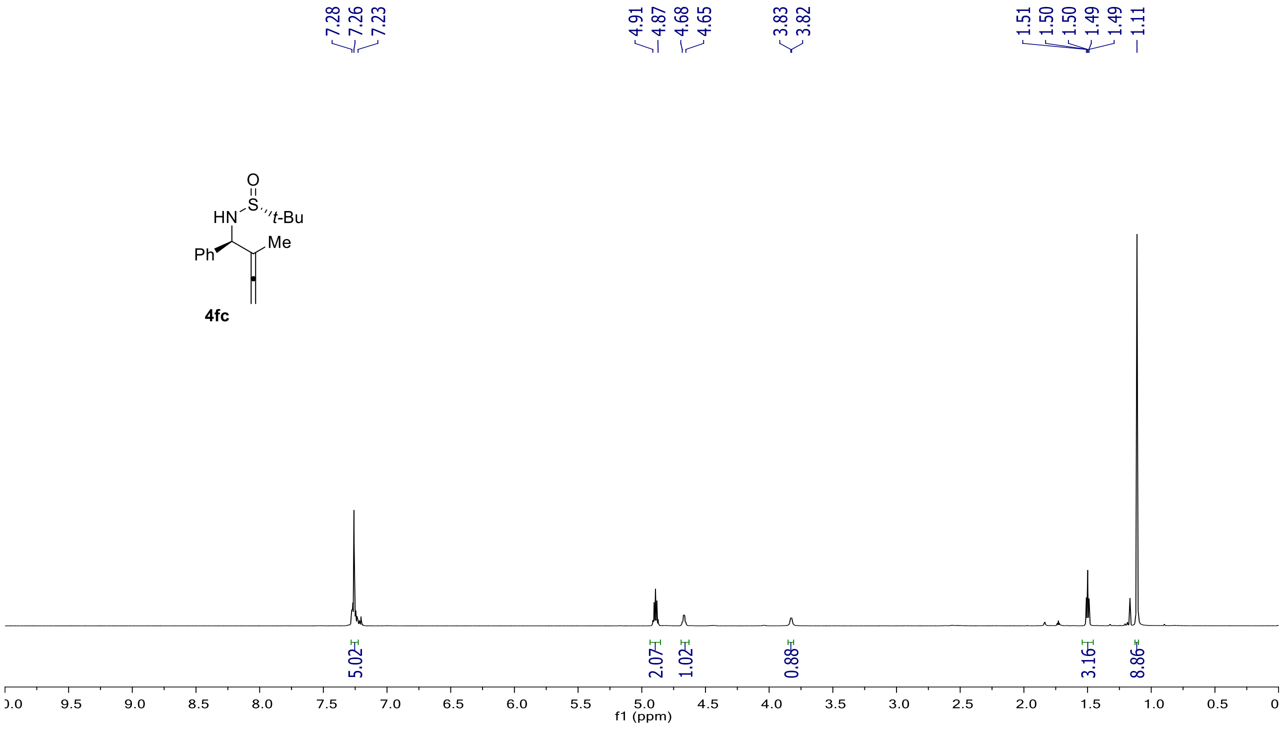

^{19}F NMR spectrum of compound **4fc** (282 MHz, $CDCl_3$)

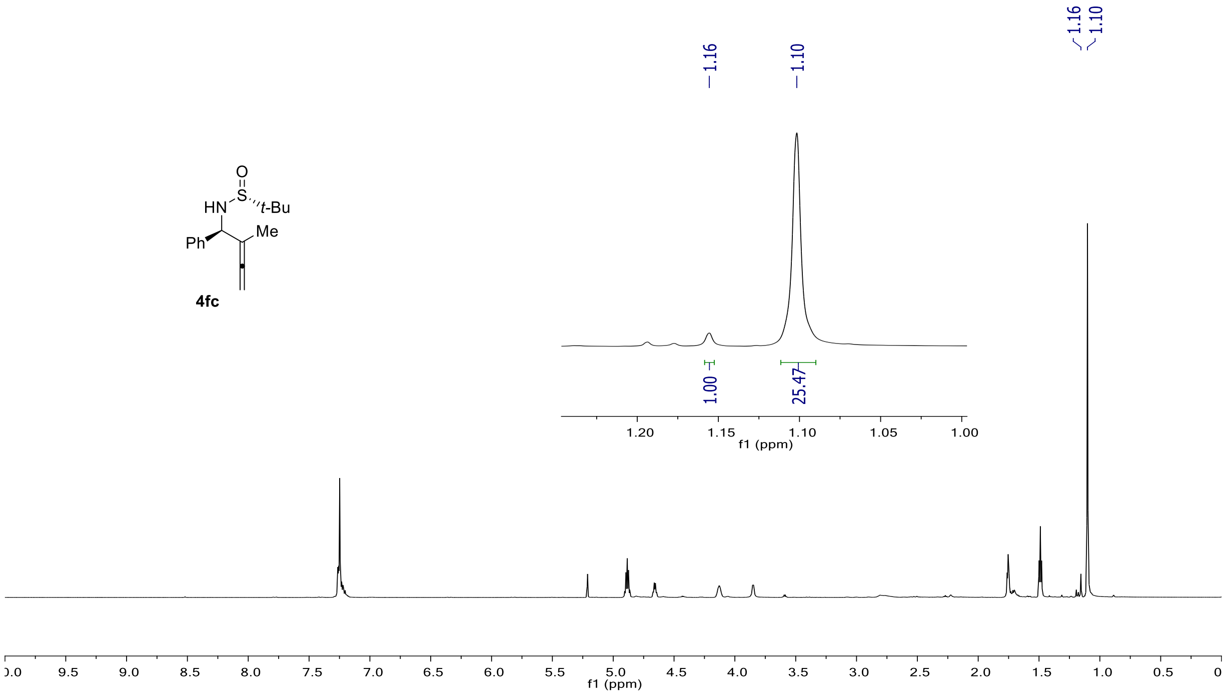

^{13}C NMR spectrum of compound **4fc** (75 MHz, $CDCl_3$)

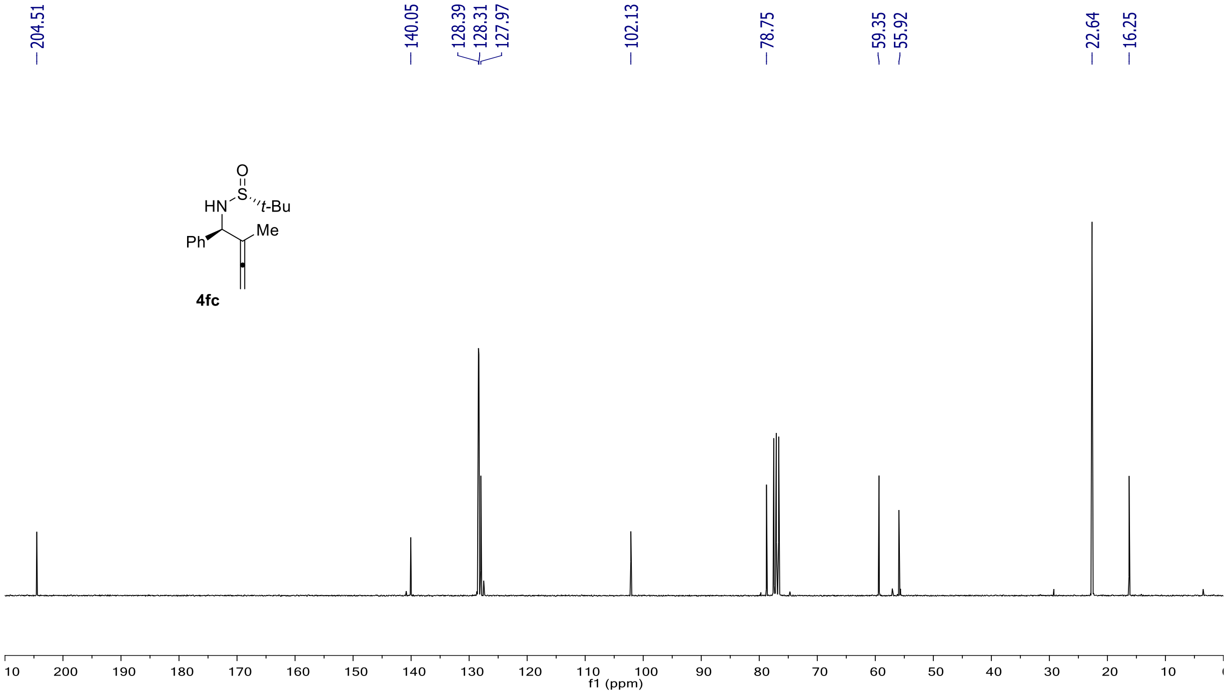

Supplement: Supplementary file 1 — ol1c01076_si_001.pdf [file ol1c01076_si_001.pdf]
